# Design, biological evaluation, and molecular modelling insights of cupressic acid derivatives as promising anti-inflammatory agents

**DOI:** 10.1080/14756366.2023.2187327

**Published:** 2023-03-13

**Authors:** Amal F. Soliman, Diaaeldin M. Elimam, Fardous F. El-Senduny, Manal A. Alossaimi, Mubarak Alamri, Fatma M. Abdel Bar

**Affiliations:** a Department of Pharmacognosy, Faculty of Pharmacy, Mansoura University, Mansoura, Egypt; b Department of Pharmacognosy, Faculty of Pharmacy, Kafrelsheikh University, Kafrelsheikh, Egypt; c Chemistry Department, Biochemistry Division, Faculty of Science, Mansoura University, Mansoura, Egypt; d Department of Pathology & Laboratory Medicine, Sylvester Comprehensive Cancer Center, Miller School of Medicine, Miami, FL, USA; e Department of Pharmaceutical Chemistry, College of Pharmacy, Prince Sattam Bin Abdulaziz University, Al Kharj, Saudi Arabia; f Department of Pharmacognosy, College of Pharmacy, Prince Sattam Bin Abdulaziz University, Al Kharj, Saudi Arabia; g Faculty of Pharmacy, Mansoura University, Mansoura, Egypt

**Keywords:** Cupressic acid derivatives, labdane diterpenoids, RAW264.7 cell line, *COX-2* gene expression, anti-inflammatory leads

## Abstract

The major labdanes in the oleogum resin of *Araucaria heterophylla* (Salisb.) Franco, 13-epi-cupressic acid (**1**) and acetyl-13-epi-cupressic acid (**2**) were used to prepare seven new (**3**–**9**), along with one known (**10**) derivatives. RAW264.7 cells were used to evaluate the anti-inflammatory activity of the derivatives (**1**–**10**) *via* measuring the level of *COX-2* expression and IL-6. Pre-treated RAW264.7 cells with **1**–**10** (except for derivative **7**) at 25 µM for 24h exhibited downregulation of *COX-2* expression in response to LPS stimulation. Moreover, pre-treatment with compounds **1**, **2**, or **3** significantly attenuated the LPS-stimulated IL-6 level in RAW264.7 cells (*p* < 0.05). A docking study was conducted against phospholipase A2 (PLA2), a crucial enzyme in initiating the inflammatory cascade. The significant structural features of compounds (**1**–**10**) as PLA2 inhibitors included the carbonyl group at C-4 (free or substituted) and the hydrophobic diterpenoid skeleton. This study suggested 13-epi-cupressic acid as a scaffold for new anti-inflammatory agents.

## Introduction

Since ancient times, plant-derived products have been used to reduce inflammation or inflammation-associated conditions ^1^. Araucaria trees are coniferous trees grown as ornamental plants, which upon injury, exudate an oleogum resin that protects against invading pathogens^2^. Few studies reported the biological activities of the oleogum resin of *Araucaria heterophylla*; mainly as an antiulcerogenic, cytotoxic, and antibacterial agent^3^. The oleogum resins, containing mainly labdane diterpenes, have been employed topically to treat bruises and dermal inflammation^4^. Diterpenes are natural products known to exert potent anti-inflammatory activities through various mechanisms^4^. Labdane-diterpenes were reported to exhibit anti-inflammatory activities in several animal and cell-based bioassays^3^^,^^4^. In addition, the analgesic and anti-inflammatory effects of semisynthetic terpene derivatives were previously described^4^. Cupressic acid derivatives are the major labdane diterpenes of the oleogum resin of *Araucaria heterophylla*. Previous studies by our research group reported the antiprotozoal activity of resin-isolated cupressic acids and the anti-inflammatory activities of their microbially transformed metabolites^2^^,^^5^.

**Figure 1.**
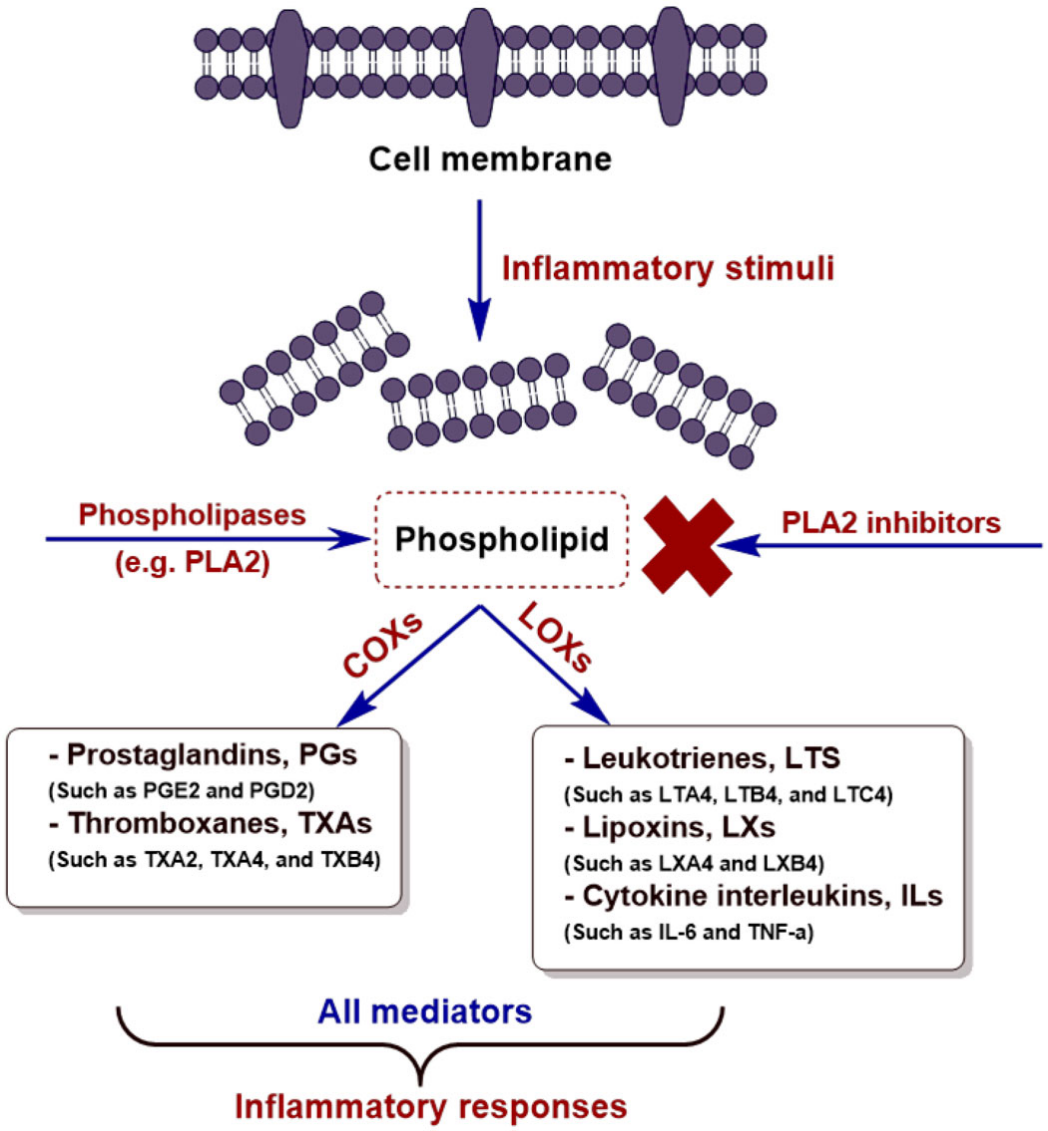
Inflammatory cascade includes the role of phospholipase A2 (PLA2) and targeting its inhibition by PLA2 inhibitors.

Physiologically, inflammatory reactions are normal immune responses against potential or already existing tissue injury. However, pathologically it may lead to, or append, multiple complications in many diseases, such as cardiovascular, pulmonary, and rheumatic diseases; in addition to malignancies, and skin disorders^6^. Phospholipase A2 (PLA2) plays a crucial role in initiating the inflammatory cascade in mammalian cells^7^. Inflammatory stimuli disrupt the cell membrane, and PLA2 starts its action on the phospholipid bilayer and releases arachidonic acid (AA), a polyunsaturated fatty acid^8^^,^^9^. At this stage, overexpression of cyclooxygenases (*COXs*) and lipoxygenases (*LOXs*) arises due to high concentrations of their AA substrate. Successively, this leads to the formation of various inflammatory mediators, including cytokine interleukins, ILs (such as IL-6 and tumour necrosis factor-alpha, TNF-*α*), prostaglandins, PGs (such as PGE2 and PGD2), thromboxanes, TXAs (such as TXA2, TXA4, and TXB4), leukotrienes, LTs (such as LTA4, LTB4, and LTC4), and lipoxins, LXs (such as LXA4 and LXB4)^8–10^. Therefore, targeting PLA2 may lead to the discovery of new PLA2 inhibitors, which can mitigate inflammation by down-inhibiting these inflammatory reactions (Figure 1).

**Figure 2.**
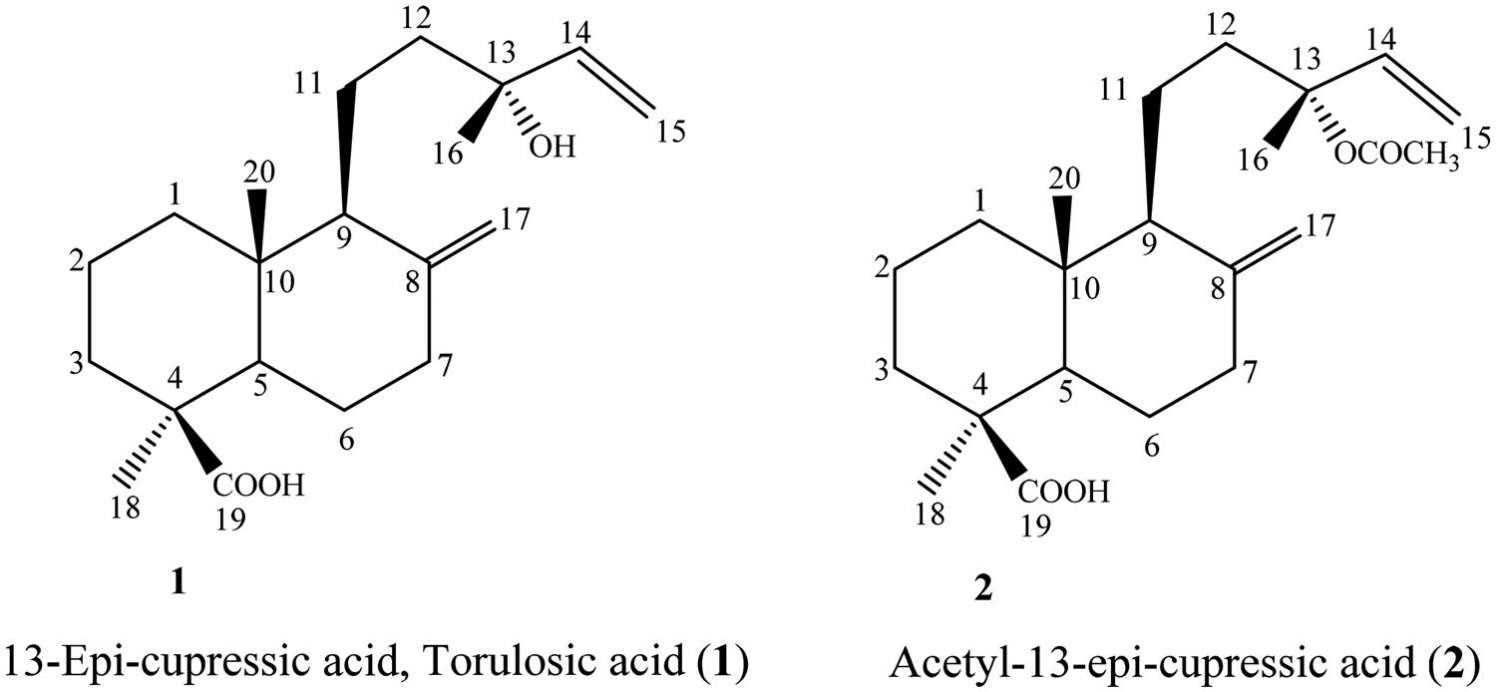
The chemical structure of isolated diterpenes from *Araucaria heterophylla* (Salisb.) Franco.

Little has been done on the semi-synthesis and biological evaluation of labdane terpenes obtained from *Araucaria heterophylla*. Cupressic acid diterpenes (Figure 2), possess a carboxylic acid moiety and a hydroxyl functional group, both of which are amenable to chemical modification and functionalization. In this work, we implemented the approach of rational, yet simple, chemical derivatization to semi-synthesize more potent and/or selective anti-inflammatory lead compounds. Additionally, a docking study has been conducted to explore the possible inhibition of PLA2 by the investigated compounds.

## Materials and methods

### General

The ^1^H and ^13^C Nuclear Magnetic Resonance (NMR) spectra were measured in CD_3_OD, with tetramethyl-silane (TMS) as the internal standard, on BRUKER Ascend ^TM^ (400 MHz) spectrometer (Bremen, Germany) operating at (400 MHz) for ^1^H and 100 MHz for ^13^C NMR. Chemical shifts (*δ*) were reported relative to TMS, as part per millions (ppm); coupling constants (*J*) in (Hz), while multiplicities of signals were expressed as: (*s* = singlet, *brs* = broad singlet, *d*= doublet, *dd* = doublet of doublet, *m* = multiplet). Processing of NMR data was done using MestReNova, v. 12.0.1-20560 (Mestrelab Research S.L., Santiago de Compostela, Spain). IR spectra were reported using a Thermo Scientific Nicolet TM iSTM10 FT-IR spectrophotometer (WI, USA). For reaction monitoring, thin layer chromatography (TLC), with precoated Silica gel aluminium plates 60 GF_254_ (Merck, Nagel, Germany) was used. Any other used solvents or reagents were of commercial grade.

### Plant materials

The resin exudates from the trunk of *Araucaria heterophylla* (Salisb.) Franco trees (Araucariaceae) were collected from the Gardens of Mansoura University campus, Mansoura, Egypt, in February 2021. The plant identity was confirmed by staff members of the Horticulture Department, Faculty of Agriculture, Mansoura University, Egypt.

### Procedures for preparation of proposed derivatives (1–10)

**Figure 3.**
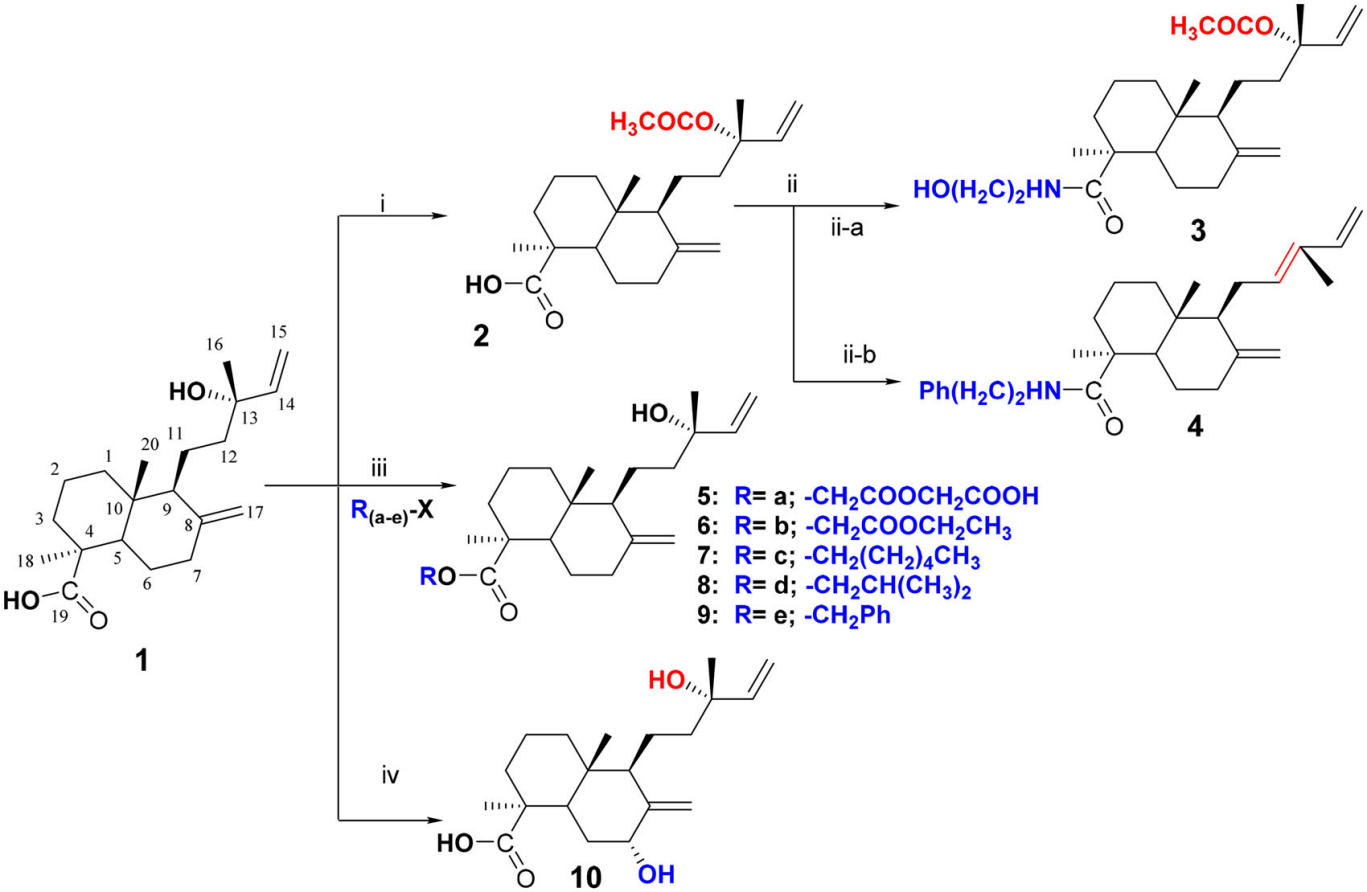
Summarised schemes (i-v) of the general preparation procedures of cupressic acid derivatives (**1**–**10**). (i) 1.5 equiv. of acetic anhydride and 2 equiv. of pyridine at room temperature for 24h; (ii) in DCM, reflux for 3h with 1.5 equiv. thionyl chloride (SOCl_2_) or for 6h with an excess of SOCl_2_; (ii-a) in DCM, reflux with 2 equiv. triethylamine (TEA) and 1.5 equiv. ethanolamine; (ii-b) in DCM, reflux with 2 equiv. 2-phenyl ethylamine at 45 °C for 48h; (iii) reflux in dry acetone, excess potassium carbonate, and a catalytic amount of DMAP; (iv) Stirring with excess selenium dioxide solution (SeO_2_) in anhydrous dioxane.

The resinous exudates (100 g) from the trunks of *Araucaria heterophylla* (Salisb.) Franco trees were extracted using petroleum ether (Pet. ether) – dichloromethane (DCM), 1:1 and dried to produce a terpenoid-rich fraction (50 g). The starting diterpenes, including the known molecules; acetyl-13-epi-cupressic acid (**2**) and 13-epi-cupressic acid, Torulosic acid (**1**)^3^, were purified from the terpenoid fraction (50 g) using liquid chromatography on normal silica. Compound **2** was eluted at 4% ethyl acetate (EtOAc)/Pet. ether (3 g, 6% yield), while compound **1** was eluted at 6% EtOAc/Pet. ether (6 g, 12% yield). Due to the limited amount available of compound **2**, it was also readily obtained through acetylation of compound **1** with 1.5 equivalent of acetic anhydride and 2 equivalent of pyridine at room temperature for 24h (Figure 3, Scheme i) with a yield of about 40%. Then 100 mg of compound **2**, dissolved in 1 ml DCM, was refluxed for 3h with 1.5 equivalent of thionyl chloride (SOCl_2_) or for 6h with an excess of SOCl_2_ (Figure 3, Scheme ii). Excess SOCl_2_ was vacuo-evaporated, and the brownish-yellow residue of the acyl chloride product (unstable to moisture), was immediately dissolved in DCM (5 ml), and refluxed with 2 equivalent of triethylamine (TEA) and 1.5 equivalent of ethanolamine in case of compound **3** (or 2-phenyl ethylamine in case of **4**) at 45 °C for 48h (Yu et al., 2006). After cooling, excess dil. HCl was added and partitioned with DCM to produce compound **3** (8.5 mg, 8.5% yield) or compound **4** (20 mg, 20%yield), (Figure 3, Scheme ii-a, b). Different alkyl halides (R_(a-e)_-X) were separately refluxed with 100 mg of compound **1** dissolved in dry acetone; in the presence of excess potassium carbonate and a catalytic amount of DMAP, to generate compounds (**5–9**), (9.2, 23, 28.3, 15.5, and 31.4 mg, respectively, 9.2, 23, 28.3, 15.5, and 31.4% yield, respectively), (Figure 3, Scheme iii). Stirring 100 mg of compound **1** with excess selenium dioxide solution (SeO_2_) in anhydrous dioxane followed by aqueous partitioning against chloroform generated compound **10** (50.8 mg, 50.8%yield), (Figure 3, Scheme iv).

### Purification of the prepared derivatives (1–10)

Derivatives **5–10** are purified over normal silica gel columns (Silica gel 60, mesh 70–230; Merck, Darmstadt, Germany) using ethyl acetate-*n*-hexane as a solvent system. They were eluted in pure form using 15, 4, 2, 3, 3, and 30% ethyl acetate in *n*-hexane, respectively. Compounds **3** and **4** were purified by preparative TLC using 100% dichloromethane as a mobile phase.

### Molecular docking

The *in vitro* assays as well as *in silico* target prediction studies implied that the mode of actions of prepared derivatives (**1**–**10**) might be mimicking that of corticosteroids, which encouraged us to run molecular docking studies to support the findings.

### Protein structures

The human phospholipase A2 complexed with a substrate analog (Substrate name, BR4: 6-phenyl-4(*R*)-(7-phenyl-heptanoylamino)-hexanoic acid), determined by X-ray crystallography (PDB code: 1KQU, with a resolution of 2.10 Å) was used in the molecular docking study of the PLA2 virtual inhibitory activities of the prepared series (https://www.rcsb.org/structure/1KQU)^11^.

### Protein preparation

The PDB protein crystal was processed by protonation, bond corrections, and fixing the potential using the MOE software. Also, the bound H_2_O molecules were removed. The active binding pocket was specified using the site-finder tool in the MOE software and it was selected by isolating atoms and backbone “dummies” at alpha centres on the basis of the 3D coordinates of the inhibitor’s nearby residues.

### Preparation of the studied ligands

The structures of the studied analogs were sketched using the ChemDraw Professional 15.0.0.0.106 © 1998–2015 (PerkinElmer Informatics Inc., MA, USA). This was followed by protonation, assigning partial charges by the current forcefield tool, and minimising energies by the general energy minimisation tool.

### Docking experiment

Molecular docking was performed by the Triangle Matcher for Placement and Rigid Receptor for Refinement. London ΔG and GBVI/WSA ΔG were selected as the scoring functions. The binding free energy of the studied analogs was calculated as kcal/mol. The highest number of negative scores obtained was chosen as the highest ligand-enzyme interaction score for the studied structures.

### Physicochemical and ADME (absorption, distribution, metabolism, and excretion) predictions

The physicochemical and ADME properties, in addition to drug-likeness, and toxicity were *in silico* investigated for the synthesised analogs using the SwissADME online tool (Swiss Institute of Bioinformatics; http://www.sib.swiss)^12^.

### Determination of cell viability by WST-1 reagent

Briefly, RAW264.7 cells (5 × 10^4^ cells/mL) were seeded in a 96-well plate, and serial dilutions of each compound (100, 50, 25, 12.5, 6.25, 3.125 µg/mL) were added in triplicates then incubated for 24h at 37 °C and 5% CO_2_. The viability was determined by using WST-1 reagent (Roche, CELLPRO-RO, Cat. No. 5015944001, Basel, Switzerland). After 1h of incubation with WST-1, the reaction was stopped by adding 10 µL of 1% SDS, and the absorbance was measured at 420 nm. The cell morphology was observed by an Optika inverted microscope (Ponteranica, Italy).

### Evaluation of the anti-inflammatory activity of the tested compounds

RAW264.7 cells were treated either with 5 µg/mL of LPS (*Escherichia coli* (O111:B4), L2630, Sigma-Aldrich) or pre-treated with test compounds at 25 µg/mL for 3h then treated with 5 µg/mL of LPS for 24h. After that, the culture media was collected for determination of the IL-6 level by an ELISA kit (FineTest™, Q-M0121-B, Wuhan, China), and the cells were collected for total RNA extraction by TRIzol reagent (Invitrogen™, Cat. No. 15596026, Carlsbad, CA, USA). Cells pre-treated with 1 µM of dexamethasone (as an anti-inflammatory drug), then treated with LPS, were used as the positive control^13^.

RNA concentration and purity were evaluated by a NanoDrop (Thermo Scientific™, Waltham, MA, USA). RNA (4 µg) was reverse transcribed to cDNA by using Top script™ RT DryMix (dN18/dN6 plus, Cat. No. RT220, Enzynomics, Daejeon, Republic of Korea) kit. The level of *COX-2* and *β-*actin was detected by SYBR Kit (SensiFAST™, Meridian Bioscience, Cincinnati, OH, USA) by using qPCR (StepOne™ Real-Time PCR, Applied Biosystems™, Waltham, MA, USA). The expression level was first normalised to the level of *β*-actin and then calculated as 2^−(ΔΔCt)^. Primers (Eurofins Scientific©, Alachua, FL, USA) are listed in Table 1.

**Table 1.** Primer sequence and their reference in NCBI.

Gene	Primer sequence	Reference sequence
*COX-2*	F: 5′-CCACTTCAAGGGAGTCTGGA	NM_011198.4
	R: 5′-AGTCATCTGCTACGGGAGGA-3′	
*β-Actin*	F:5-GGG AAA TCG TGC GTG ACA T-3	NM_007393.5
	R:5-GCG GCA GTG GCC ATC TC-3	

### Statistical analysis

The statistical analysis was performed by comparison of means obtained from triplicate treatments with the One-Way Analysis of variance “ANOVA” method on GraphPad Prism Version 8.0 (GraphPad Software, Inc., San Diego, CA). Differences between groups were significant at “*p* values” of <0.05.

## Results and discussion

### Preparation of the proposed derivatives (1–10)

The starting material (13-epi-cupressic acid, Torulosic acid, **1**) was isolated from the oleogum resin of *Araucaria heterophylla* (Salisb.) Franco and subjected to acetylation reaction to yield acetyl-13-epi-cupressic acid, **2** (Scheme i, Figure 3). A reactive acyl chloride derivative was produced from the protected acetyl compound (**2**) and reacted with a nucleophile (ethanolamine) to produce the amide derivative **3** (Scheme ii-a, Figure 3). Interestingly, upon repeating the same reaction under more drastic conditions, oxidative deacetylation took place to form communic acyl chloride which upon reacting with a nucleophile (phenylethylamine) produced the carbamoyl derivative, **4** (Scheme ii-b, Figure 3). The ester derivatives (**5**–**9**) were produced through the coupling of the potassium salt of compound **1** with different alkyl halides (Scheme iii, Figure 3). Allylic hydroxylation of the bicyclic diterpene (**10**) was achieved through the oxidation of compound **1** with SeO_2_ (Scheme iv, Figure 3).

### Structural identification of the prepared derivatives (1–10)

Spectroscopic (FT-IR, NMR & MS) and elemental analyses were employed to confirm the structures of the prepared compounds, **1–10** (Figure 3). Free OH/NH groups showed broad absorption peaks at *ν* (3600–3200 cm^−1^). Upon inspection of NMR spectra, convergent values - with slight variations – were observed among all analysed compounds (**1**–**10**), as expected, due to the common bicyclic system with its exocyclic double bond and carbonyl group in addition to the chain-terminal double bond. The ^1^H NMR spectra (Table S2) displayed a signal of double doublet olefinic proton at *δ*_H_ 6.33–5.83 (coupling constants *J* = 17.4–16.0 *Hz*, 12.0–10.8 *Hz*, H-14) and two doublet protons at *δ*_H_ 5.25–4.95, coupled with the adjacent methine proton (*J* = 17.4–10.8 *Hz*, H-15); both indicating the chain-terminal double bond. The exocyclic double bond was commonly annotated by 2 singlet protons (broad singlets) at *δ*_H_ 4.93–4.16 (H-17); while the three methyl groups were presented by 3 distinct upfield singlet signals at *δ*_H_ 1.75–1.09 (H-16/18) or *δ*_H_ 0.63–0.45 (H-20). ^13^C NMR spectra (DEPT/APT experiments) displayed extremely downfield quaternary carbon signals at *δ*_C_ 183.2–171.9 (C-19) assigned to the carbonyl carbon next to the bicyclic system (Table S1). The exocyclic double bond showed a downfield quaternary carbon signal at *δ*_C_ 150.9–147.7 (C-8) and up-field secondary carbon at *δ*_C_ 107.7–106.6 (C-17), while the chain terminal double bond showed a downfield primary carbon signal at *δ*_C_147.9–141.5 (C-14) and upper-field secondary carbon at *δ*_C_ 113.5–109.8 (C-15). Generally, the upfield tertiary carbon signals, including two at *δ*_C_ 30.4–22.9 (C-16/C-18) and one at *δ*_C_ 13.0–12.4 (C-20), represented the common 3 methyl groups. Other eleven upfield aliphatic carbon signals (Table S1) indicated the rest of the typical backbone of labdane-diterpene in all derivatives. Compounds **2** and **3** showed extra methyl signals corresponding to the methyl groups of the introduced acyl moiety, in each case. This includes the resonances at *δ*_H_ 1.96 (–COCH_3_, in **2** and **3**). Also, the ^13^C NMR spectra of **2** and **3** showed an extra downfield quaternary signal at *δ*_C_ 169.8–169.3 (–COCH_3_) and an upfield methyl signal at *δ*_C_ 22.1–20.9 (–COCH_3_) corresponding to the acetyl group on the side chain-hydroxyl group at C-13.

### Characterisation of compounds 3 and 4

The FT-IR*ν*_max_(neat) spectra of amide derivatives (**3** and **4**) showed absorption bands for (NH) and (C=O) functional groups in the regions υ (3400–3000 cm^−1^) and (1750–1710 cm^−1^), respectively. The ^1^H NMR spectrum of compound (**3**) showed the presence of a methyl singlet at 1.96 of the acetyl group at C-13 (Table S2 and Figure S6). Whereas no acetyl group was detected in the case of **4**, it rather showed a downfield triplet at *δ*_H_ 5.36 (H-12) and a relatively upfield multiplet at *δ*_H_ 2.33 (H-11), which indicated the introduction of an additional double bond between C-12:13 creating a terminal conjugated diene (Table S2 and Figure S13). Due to the newly introduced conjugated double bond, the ^13^C NMR and APT spectra of compound **4** (Figures S14, S15) showed two extra downfield carbon signals, including a methine at *δ*_C_ 134.0 (C-12) and quaternary at *δ*_C_ 133.5 (C-13). Further evidence for the loss of the esterified OH group was indicated by the absence of a quaternary signal at *δ*_C_ 83–85 (C-13) compared to the related derivatives (**2** and **3** and the presence of upfield methyl carbon signals at *δ*_C_ 11.9 (C-16). Regarding the introduced substitutions at C-19, the ^13^C NMR and APT spectra of compound **3** showed two characteristic methylene signals at *δ*_C_ 42.8 (N–CH_2_) and 61.5 (O–CH_2_), for C-1″ and C-2″ of the ethanolamine moiety, respectively. Similarly, the ^13^C NMR and APT spectra of compound **4** showed characteristic resonances at *δ*_C_ 138.9 (C-1″; qC), 128.6 (CH; C2″, 3″, 5″, and 6″), and 126.4 (CH; C-4′), 40.6 (C-1′; N–CH_2_), and 35.3 (C-2′; Ph–CH_2_) for the introduced phenylethylamine moiety. Thus, the structures of **3** and **4** were confirmed as the new structures; 13-acetyl, 19-(2-hydroxyethyl)carbamoyl-13-epi-cupressic acid and 19-(2-phenylethyl)carbamoyl-communic acid, respectively.

### Characterisation of compounds 5–9

The FT-IR*ν*_max_(neat) spectra of the ester derivatives (**5–9**) showed characteristic peaks for (C=O) and (C–O) groups at υ (1730–1700 cm^−1^) and (1250–1055 cm^−1^), respectively. The ^1^H NMR spectra of compounds **6**–**8** showed extra methyl signals corresponding to the methyl groups of the introduced acyl moiety, in each case. This includes the resonances at *δ*_H_ 1.20 (H-4′ in **6**), 0.81 (H-6′ in **7**), and 0.97, 0.99 (H-3′,4′ in **8**). Moreover, compounds (**5–9**) showed ^1^H and ^13^C NMR signals corresponding to the ester moieties at C-19, each was consistent with those of the reagent used for its preparation (Table S2). The ^13^C NMR spectra of compounds **5** and **6** showed two downfield signals at *δ*_C_ 167.6 and 167.8 due to the ester carbonyl carbons (C-2′) of the esterifying 2-ethoxy-2-oxoethyl group in **5** (or ethyl 2-hydroxyacetate moiety in **6**) on the C-19 carboxylic group, respectively. Moreover, compounds (**5–9**) showed upfield aliphatic signals that indicate the sequel of the ester moieties on the carboxylic acid group as indicated above. Compound **5** showed two aliphatic methylene groups as indicated by the two doublets (*J* = 16 Hz) at *δ*_H_ 4.54 and 4.68 (H-1′; *δ*_C_ 60.8) and the doublet at *δ*_H_ 4.61 (*J* = 2 Hz; *δ*_C_ 60.0, H-3′) for the 2-ethoxy-2-oxoethyl group. Similarly, **6** showed two doublets (*J* = 15.7 Hz) at *δ*_H_ 4.65 and 4.52 (H-1′; *δ*_C_ 61.2), a doublet of doublet signal (*J* = 7.2, 14.3 Hz) at *δ*_H_ 4.25 (H-2′; *δ*_C_ 60.4) for the two methylene groups of ethyl 2-hydroxyacetate. However, compound **7** showed a proton multiplet signal at *δ*_H_ 3.92 (H-1′; *δ*_C_ 64.2), in addition to the resonances at *δ*_H_ 1.65 (H-2′; *δ*_C_ 28.3), 1.28–1.57 (H-3′-5′; *δ*_C_ 25.7, 31.3, 22.5), 0.81 (H-6′; *δ*_C_ 14.0) for the hexyloxyl group. Whereas **8** showed two doublets of doublet signals (*J* = 6.2, 10.6 Hz) at *δ*_H_ 3.78 and 3.89 (H-1′; *δ*_C_ 70.5), a multiplet at *δ*_H_ 1.91 (H-2′; *δ*_C_ 27.6), and two methyl doublet signals (*J* = 2.4 Hz) at *δ*_H_ 0.97 and 0.99 (H-3′; *δ*_C_ 19.3 and H-4′; *δ*_C_ 19.4, respectively) for the isobutyloxyl group. Compound **9** showed distinctive aromatic protons at *δ*_H_ 7.34–7.37 (Figure S35) and their corresponding ^13^C NMR resonances at *δ*_C_ 138.9–126.4 for the inserted phenyl moiety (Figure S36). It is worth noting that the obtained derivative (**5**–**9**) are new structures namely, 19-(2-(2-hydroxyacetoxy)acetic acid)-13-epi-cupressic acid (**5**), 19-(2-ethoxy-2-oxoethyl)-13-epi-cupressic acid (**6**), 19-hexyl-13-epi-cupressic acid (**7**), 19-isobutyl-13-epi-cupressic acid (**8**), and 19-benzyl-13-epi-cupressic acid (**9**).

### Characterisation of compounds 10

The FT-IR*ν*_max_(neat) spectra of compound **10** showed prominent peaks for (O–H) and (C = C) groups at υ (3446 cm^−1^) and (1692 cm^−1^), respectively. The ^1^H NMR spectrum of compound **10** (Figure S39) showed more downfield split (triplet) signal at *δ_H_* 4.29 (H-7) corresponding to the proton geminal to the newly introduced hydroxyl group at C-7 (Table S2 and Figure S39). The ^13^C NMR and APT spectra of compound **10** (Figure S40 and S41) showed a more downfield oxymethine signal at *δ*_C_ 74.8 (C-7) corresponding to the newly introduced hydroxyl group located at the allylic carbon to the exocyclic double bond^14^. Thus, the structure of compound **10** was determined as 7α-hydroxy-13-epi-cupressic acid, which was previously obtained before by microbial transformation of cupressic acid by *Fusarium graminearum*^14^.

### (1S,4aR,5S)-5-((R)-3-Hydroxy-3-methylpent-4-en-1-yl)-1,4a-dimethyl-6-methylenedecahydronaphthalene-1-carboxylic acid (1)

Semisolid resinous mass, IR (KBr, *ν* cm^−1^) 3418 broad (OH), 2930 (COOH), 2850 (–CH), 1696 (C=O), and 1646 (C = C). ^1^H NMR (400 MHz, CDCl_3_) *δ*_H_ 5.95 (dd, *J* = 16.0 Hz, 12.0 Hz, 1H, –C**H**=CH_2_), 5.24 (d, *J* = 12.0 Hz, 1H, –CH = C**H_2_**), 5.08 (d, *J* = 16.0 Hz, 1H, –CH = C**H_2_**), 4.56 (brs, 1H, –C = C**H_2_**), 4.87 (brs, 1H, –C = C**H_2_**), 2.30–0.8 (m, 16H), 1.30 (s, 3H, –C**H_3_**), 1.26 (s, 3H, –C**H_3_**), 0.63 (s, 3H, –C**H_3_**). ^13^C NMR (DEPT 101 MHz, CDCl_3_) *δ* 183.0 (–**C**OOH), 148.0 (–**C**=CH_2_), 145.0 (–**C**H = CH_2_), 111.5 (–CH=**C**H_2_), 106.6 (–C=**C**H_2_), 73.6 (**C–**OH), 56.7, 56.4, 44.2, 41.5, 40.7, 39.2, 38.4, 38.0, 26.0, 29.2 (–**C**H_3_), 27.6 (–**C**H_3_), 19.9, 17.9, 12.7 (–**C**H_3_). MS (ESI), *m/z* for C_20_H_32_O_3_, Calc 320.4730, Found 319 [M − H]^-^; Elemental Analysis: C, 74.96; H, 10.07; O, 14.98, Found C, 74.93; H, 10.10; O, 14.95.

### (1S,4aR,5S)-5-((R)-3-Acetoxy-3-methylpent-4-en-1-yl)-1,4a-dimethyl-6-methylenedecahydronaphthalene-1-carboxylic acid (2)

Semisolid resinous mass **(**yield 40%), IR (KBr, *ν* cm^−1^) 3802 − 2930 (COOH), 2853 (–CH), 1737 (C=OOC), 1694 (C=O), 1645 (C = C), 1250 (C–C–O), and 1180 (O–C–C). ^1^H NMR (400 MHz, CDCl_3_) *δ*_H_ 5.89 (dd, *J* = 16.0 Hz, 12.0 Hz, 1H, –C**H**=CH_2_), 5.04 (d, *J* = 12.0 Hz, 1H, –CH = C**H_2_**), 5.02 (d, *J* = 16.0 Hz, 1H, –CH = C**H_2_**), 4.77 (brs, 1H, –C = C**H_2_**), 4.44 (brs, 1H, –C = C**H_2_**), 2.30–0.8 (m, 16H), 1.96 (s, 3H, –CO–C**H_3_**), 1.45 (s, 3H, –C**H_3_**), 1.16 (s, 3H, –C**H_3_**), 0.53 (s, 3H, –C**H_3_**). ^13^C NMR (DEPT 101 MHz, CDCl_3_) *δ* 183.2 (–**C**OOH), 169.8 (–COOC), 148.0 (–**C**=CH_2_), 141.9 (–**C**H = CH_2_), 113.0 (–CH=**C**H_2_), 106.6 (–C=**C**H_2_), 83.3 (**C–**OH), 56.6, 56.4, 44.2, 44.2, 40.6, 39.2, 38.7, 38.0, 36.9, 36.9, 26.0, 28.9 (–**C**H_3_), 23.5 (–**C**H_3_), 22.1 (–CH_3_), 19.9, 17.6, 12.7 (–**C**H_3_). MS (ESI), *m/z* for C_22_H_34_O_4_, Calc 362.5100, Found 361 [M − H]^-^; Elemental Analysis: C, 72.89; H, 9.45; O, 17.65, Found C, 72.68; H, 9.40; O, 17.60.

### (3R)-5-((1S,5S,8aR)-5-((2-Hydroxyethyl)carbamoyl)-5,8a-dimethyl-2-methylenedecahydronaphthalen-1-yl)-3-methylpent-1-en-3-yl acetate (3)

Semisolid resinous mass **(**yield 8.5%), IR (KBr, *ν* cm^−1^), 3540–3510 (OH), 3430 − 3390 broad (CONH), 2925 (–CH), 1710, 1690 (C=O), 1610, 1514 (C = C), 1235 (C–N). ^1^H NMR (600 MHz, CD_3_OD) *δ*_H_ 7.18 (t, 1H, –CON**H**–), 5.95 (dd, *J* = 16.0 Hz, 12.0 Hz, 1H, –C**H**=CH_2_), 5.14 (d, *J* = 12.0 Hz, 1H, –CH = C**H_2_**), 5.08 (d, *J* = 16.0 Hz, 1H, –CH = C**H_2_**), 4.87 (brs, 1H, –C = C**H_2_**), 4.56 (brs, 1H, –C = C**H_2_**), 3.56 (t, 2H, HO–C**H_2_–**), 3.26 (m, 2H, N–C**H_2_**), 2.30–0.8 (m, 16H), 1.96 (s, 3H, –CO–C**H_3_**), 1.30 (s, 3H, –C**H_3_**), 1.26 (s, 3H, –C**H_3_**), 0.63 (s, 3H, –C**H_3_**). ^13^C NMR (APT 151 MHz, CD_3_OD) *δ* 171.9 (–**C**OOH), 169.3 (–COC), 148.9 (–**C**=CH_2_), 143.2 (–**C**H = CH_2_), 113.5 (–CH=**C**H_2_), 106.8 (–C=**C**H_2_), 84.9 (**C–**OH), 61.5, 58.3, 58.2, 45.3, 42.8, 41.5, 40.5, 40.1, 39.1, 38.2, 37.8, 30.4 (–**C**H_3_), 24.6, 22.9 (–CH_3_), 21.3, 20.9 (–**C**H_3_), 18.9, 12.5 (–**C**H_3_). MS (ESI), *m/z* for C_24_H_39_NO_4_, Calc 405.5790, Found 406 [M + H]^+^; Elemental Analysis: C, 71.07; H, 9.69; N, 3.45; O, 15.78, Found C, 71.10; H, 9.66; N, 3.42; O, 15.82.

### (1S,4aR,5S)-1,4a-Dimethyl-6-methylene-5-((E)-3-methylpenta-2,4-dien-1-yl)-N-phenethyldecahydronaphthalene-1-carboxamide (4)

Semisolid resinous mass **(**yield 20%), IR (KBr, *ν* cm^−1^) 3450 − 3400 broad (CONH), 2932 (–CH), 1715 (C=O), 1640, 1521, 1452 (C = C), 1242 (C–N) and 750–700 (Ar–C). ^1^H NMR (500 MHz, CDCl_3_) *δ*_H_ 7.21–7.04 (m, 5H, Ar–**H**), 7.20 (m, 1H, –CON**H**–), 6.33 (dd, *J* = 17.0 Hz, 12.0 Hz, 1H, –C**H**=CH_2_), 5.36 (t, 1H, –C**H**=C–), 5.20 (d, *J* = 12.0 Hz, 1H, –CH = C**H_2_**), 5.04 (d, *J* = 17.0 Hz, 1H, –CH = C**H_2_**), 4.80 (brs, 1H, –C = C**H_2_**), 4.43 (brs, 1H, –C = C**H_2_**), 3.48 (m, 2H, N–C**H_2_**), 2.80 (t, 2H, Ph–C**H_2_–**), 2.31 (m, 2H, =CH–C**H_2_–**), 2.30–0.8 (m, 16H), 1.96 (s, 3H, –CO–C**H_3_**), 1.75 (s, 3H, –C**H_3_**), 1.09 (s, 3H, –C**H_3_**), 0.53 (s, 3H, –C**H_3_**). ^13^C NMR (DEPT 125 MHz, CDCl_3_) *δ* 176.5 (–**C**OOH), 147.7 (–**C**=CH_2_), 141.5 (–**C**H = CH_2_), 138.9 (–**C = C, Ar**), 134.0 (–C = CH), 133.5 (–**C**=CH), 128.6–126.4 (–CH = CH–, Ar), 109.8 (–CH=**C**H_2_), 107.7 (–C=**C**H_2_), 56.5, 56.3, 44.2, 43.1, 40.6, 39.6, 39.5, 38.7, 38.4, 36.9, 35.3, 30.2 (–**C**H_3_), 26.4, 23.3, 20.2, 13.0 (–**C**H_3_), 11.9 (–**C**H_3_). MS (ESI), *m/z* for C_28_H_39_NO, Calc 405.6260, Found 406 [M + H]^+^; Elemental Analysis: C, 82.91; H, 9.69; N, 3.45; O, 3.94, Found: C, 82.94; H, 9.65; N, 3.49; O, 3.91.

### 2-(2-(((1S,4aR,5S)-5-((R)-3-hydroxy-3-methylpent-4-en-1-yl)-1,4a-dimethyl-6-methylenedecahydronaphthalene-1-carbonyl)oxy)acetoxy)acetic acid (5)

Semisolid resinous mass **(**yield 9.2%), IR (KBr, *ν* cm^−1^) 3560 − 3530 (OH), 3445 (COOH), 2958, 2927, 2854 (–CH), 1770, 1747, 1710 (C=O), 1644, 1608 (C = C), and 1290, 1210, 1147, 1095 (O–C). ^1^H NMR (400 MHz, CDCl_3_) *δ*_H_ 5.84 (dd, *J* = 16.0 Hz, 12.0 Hz, 1H, –C**H**=CH_2_), 5.10 (d, *J* = 12.0 Hz, 1H, –CH = C**H_2_**), 4.99 (d, *J* = 16.0 Hz, 1H, –CH = C**H_2_**), 4.77 (brs, 1H, –C = C**H_2_**), 4.68 (d, *geminal J* = 16.0 Hz, 1H, COOC–C**H**_2_–COOC), 4.61 (d, *J* = 2.0 Hz, 2H, OC–C**H_2_**–COOH), 4.54 (d, *geminal J* = 16.0 Hz, 1H, OC–C**H**_2_–CO), 4.45 (brs, 1H, –C = C**H_2_**), 2.30–0.8 (m, 16H), 1.96 (s, 3H, –CO–C**H_3_**), 1.45 (s, 3H, –C**H_3_**), 1.16 (s, 3H, –C**H_3_**), 0.53 (s, 3H, –C**H_3_**). ^13^C NMR (APT 101 MHz, CDCl_3_) *δ* 176.6 (–**C**OOH), 167.6 (–CO), 167.4 (–COOH), 147.9 (–**C**=CH_2_), 145.2 (–**C**H = CH_2_), 111.6 (–CH=**C**H_2_), 106.7 (–C=**C**H_2_), 73.5 (**C–**OH), 60.8 (OC–**C**H_2_–CO), 60.0 (OC–**C**H_2_–CO), 56.5, 56.3, 44.5, 44.2, 41.4, 40.5, 39.1, 38.7, 38.4, 36.9, 28.9 (–**C**H_3_), 27.6 (–**C**H_3_), 26.0, 19.9, 17.9, 12.8 (–**C**H_3_). MS (ESI), *m/z* for C_24_H_36_O_7_, Calc 436.5450, Found 435 [M − H]^-^; Elemental Analysis: C, 66.03; H, 8.31; O, 25.65, Found 66.10; H, 8.35; O, 25.62.

### 2-Ethoxy-2-oxoethyl (1S,4aR,5S)-5-((R)-3-hydroxy-3-methylpent-4-en-1-yl)-1,4a-dimethyl-6-methylenedecahydronaphthalene-1-carboxylate (6)

Semisolid resinous mass **(**yield 23%), IR (KBr, *ν* cm^−1^) 3550 − 3520 (OH), 2958, 2927, 2854 (–CH), 1764 (C=O), 1737 (C=O), 1643, 1603 (C = C), and 1285, 1208, 1144, 1093 (O–C). ^1^H NMR (400 MHz, CDCl_3_) *δ*_H_ 5.90 (dd, *J* = 17.4 Hz, 10.8 Hz, 1H, –C**H**=CH_2_), 5.25 (dd, *J* = 10.8 Hz, 1.2 Hz, 1H, –CH = C**H_2_**), 5.10 (dd, *J* = 17.4 Hz, 1.2 Hz, 1H, –CH = C**H_2_**), 4.93 (brs, 1H, –C = C**H_2_**), 4.65 (d, *geminal J* = 15.7 Hz, 1H, COOC–C**H**_2_–COOC), 4.63 (brs, 1H, –C = C**H_2_**), 4.52 (d, *geminal J* = 15.7 Hz, 1H, COOC–C**H**_2_–COOC), 4.25 (dd, *J* = 14.3 Hz, 7.2 Hz, 2H, –C**H_2_–**CH_3_), 2.30–0.8 (m, 16H), 1.20 (m, 3H, –CO–CH_2_–C**H_3_**), 1.19 (s, 3H, –C**H_3_**), 1.17 (s, 3H, –C**H_3_**), 0.60 (s, 3H, –C**H_3_**). ^13^C NMR (APT 101 MHz, CDCl_3_) *δ* 176.5 (–**C**OOH), 167.8 (–CO), 148.2 (–**C**=CH_2_), 147.9 (–**C**H = CH_2_), 111.5 (–CH=**C**H_2_), 106.8 (–C=**C**H_2_), 73.4 (**C–**OH), 61.2 (OC–**C**H_2_–CO), 60.4 (OC–**C**H_2_–CO), 56.6, 56.5, 44.5, 41.5, 40.4, 39.2, 38.8, 38.4, 27.9, 27.2 (–**C**H_3_), 22.8 (–**C**H_3_), 20.0, 18.0, 14.13 (–**C**H_3_), 12.9 (–CH3). MS (ESI), *m/z* for C_24_H_38_O_5_, Calc 406.5630, Found 408 [M + H]^+^; Elemental Analysis: C, 70.90; H, 9.42; O, 19.68, Found C, 70.93; H, 9.40; O, 19.65.

### Hexyl (1S,4aR,5S)-5-((R)-3-hydroxy-3-methylpent-4-en-1-yl)-1,4a-dimethyl-6-methylenedecahydronaphthalene-1-carboxylate (7)

Semisolid resinous mass **(**yield 28.3%), IR (KBr, *ν* cm^−1^) 3530 − 3500, broad (OH), 2958, 2933, 2852 (–CH), 1721 (C=O), 1644, 1464 (C = C), and 1156 (O–C). ^1^H NMR (400 MHz, CDCl_3_) *δ*_H_ 5.83 (dd, *J* = 17.4 Hz, 10.8 Hz, 1H, –C**H**=CH_2_), 5.10 (dd, *J* = 10.8 Hz, 1.8 Hz, 1H, –CH = C**H_2_**), 4.95 (dd, *J* = 17.4 Hz, 1.8 Hz, 1H, –CH = C**H_2_**), 4.76 (brs, 1H, –C = C**H_2_**), 4.45 (brs, 1H, –C = C**H_2_**), 3.92 (m, 2H, CO–C**H_2_–**CH_2_), 2.30–0.8 (m, 24H), 1.23 (s, 3H, –C**H_3_**), 1.17 (s, 3H, –C**H_3_**), 0.81 (t, 3H, –CH_2_–C**H_3_**), 0.45 (s, 3H, –C**H_3_**). ^13^C NMR (APT 150 MHz, CDCl_3_) *δ* 177.3 (–**C**OOH), 147.8 (–**C**=CH_2_), 145.2 (–**C**H = CH_2_), 111.5 (–CH=**C**H_2_), 106.6 (–C=**C**H_2_), 73.4 (**C–**OH), 64.2, 56.5, 56.4, 44.3, 41.4, 40.5, 39.2, 38.8, 38.2, 31.3, 28.9 (–**C**H_3_), 28.3, 27.5 (–**C**H_3_), 26.2, 25.7, 22.5, 19.9, 17.9, 14.0 (–**C**H_3_), 12.7 (–**C**H_3_). MS (ESI), *m/z* for C_26_H_44_O_3_, Calc 404.6350, Found 402.6 [M − 2H]; Elemental Analysis: C, 77.18; H, 10.96; O, 11.86, Found C, 77.34; H, 10.36; O, 11.05.

### Isobutyl (1S,4aR,5S)-5-((R)-3-hydroxy-3-methylpent-4-en-1-yl)-1,4a-dimethyl-6-methylenedecahydronaphthalene-1-carboxylate (8)

Semisolid resinous mass **(**yield 15.5%), IR (KBr, *ν* cm^−1^) 3530 − 3500, broad (OH), 2960, 2928, 2853 (–CH), 1723 (C=O), 1464 (C = C), and 1154 (O–C). ^1^H NMR (400 MHz, CDCl_3_) *δ*_H_ 5.95 (dd, *J* = 16.0 Hz, 12.0 Hz, 1H, –C**H**=CH_2_), 5.24 (d, *J* = 12.0 Hz, 1H, –CH = C**H_2_**), 5.08 (dd, *J* = 16.0 Hz, 1H, –CH = C**H_2_**), 4.87 (brs, 1H, –C = C**H_2_**), 4.56 (brs, 1H, –C = C**H_2_**), 3.78 (dd, *J* = 10.6 Hz, 6.2 Hz, 2H, CO–C**H_2_–**CH–), 2.30–0.8 (m, 17H), 1.30 (s, 3H, –C**H_3_**), 1.26 (s, 3H, –C**H_3_**), 0.99 (d, *J* = 2.3, 3H, –C**H_3_**), 0.97 (d, *J* = 2.3, 3H, –C**H_3_**), 0.63 (s, 3H, –C**H_3_**). ^13^C NMR (APT 101 MHz, CDCl_3_) *δ* 177.2 (–**C**OOH), 148.0 (–**C**=CH_2_), 145.3 (–**C**H = CH_2_), 111.5 (–CH=**C**H_2_), 106.7 (–C=**C**H_2_), 73.4 (**C–**OH), 70.5, 56.8, 56.6, 44.4, 41.5, 40.5, 39.4, 39.2, 39.1, 38.5, 29.5 (–**C**H_3_), 27.6 (–**C**H_3_), 26.3, 20.7, 19.4 (–**C**H_3_), 19.3 (–**C**H_3_), 18.0, 12.7 (–**C**H_3_). MS (ESI), *m/z* for C_24_H_40_O_3_, Calc 376.5810, Found 376.2 [M]; Elemental Analysis C, 76.55; H, 10.71; O, 12.75, Found C, 76.61; H, 10.81; O, 12.61.

### Benzyl (1S,4aR,5S)-5-((R)-3-hydroxy-3-methylpent-4-en-1-yl)-1,4a-dimethyl-6-methylenedecahydronaphthalene-1-carboxylate (9)

Semisolid resinous mass **(**yield 31.4%), IR (KBr, *ν* cm^−1^) 3532– 3502, broad (OH), 2961, 2936, 2847 (–CH), 1722 (C=O), 1643, 1455 (C = C), 1155 (O–C), and 750–690 (Ar–C). ^1^H NMR (400 MHz, CDCl_3_) *δ*_H_ 7.37–7.34 (m, 5H, Ar–H), 5.95 (dd, *J* = 16.0 Hz, 12.0 Hz, 1H, –C**H**=CH_2_), 5.24 (d, *J* = 12.0 Hz, 1H, –CH = C**H_2_**), 5.08 (dd, *J* = 16.0 Hz, 1H, –CH = C**H_2_**), 4.87 (brs, 1H, –C = C**H_2_**), 4.56 (brs, 1H, –C = C**H_2_**), 4.67 (s, 2H, CO–C**H_2_–**Ph), 2.30–0.8 (m, 17H), 1.30 (s, 3H, –C**H_3_**), 1.26 (s, 3H, –C**H_3_**), 0.99 (d, *J* = 2.3, 3H, –C**H_3_**), 0.97 (d, *J* = 2.3, 3H, –C**H_3_**), 0.63 (s, 3H, –C**H_3_**). ^13^C NMR (APT 101 MHz, CDCl_3_) *δ* 177.1 (–**C**OOH), 148.0 (–**C**=CH_2_), 145.2 (–**C**H = CH_2_), 136.0 (Ar–**C**H_2_–), 128.5–126.9 (–CH = CH–, Ar), 111.6 (–CH=**C**H_2_), 106.7 (–C=**C**H_2_), 73.5 (**C–**OH), 66.0, 56.5, 44.4, 41.4, 40.5, 39.1, 38.7, 38.2, 29.7 (–**C**H_3_), 27.5 (–**C**H_3_), 26.7, 20.0, 18.0, 12.7 (–**C**H_3_). MS (ESI), *m/z* for C_27_H_38_O_3_, Calc 410.5980, Found 410 [M]; Elemental Analysis: C, 78.98; H, 9.33; O, 11.69 Found C, 78.87; H, 9.29; O, 11.75.

### (1S,4aR,5R,7R,8aR)-7-hydroxy-5-((R)-3-hydroxy-3-methylpent-4-en-1-yl)-1,4a-dimethyl-6-methylenedecahydronaphthalene-1-carboxylic acid (10)

Semisolid resinous mass **(**yield 50.8%), IR (KBr, *ν* cm^−1^) 3418 broad (OH), 2930 (COOH), 2850 (–CH), 1696 (C=O), and 1646 (C = C). ^1^H NMR (400 MHz, CDCl_3_) *δ*_H_ 5.90 (dd, *J* = 10.8 Hz, 17.4 Hz, 1H, –C**H**=CH_2_), 5.18 (dd, *J* = 10.8 Hz, 1.8 Hz,1H, –CH = C**H_2_**), 5.01 (d, *J* = 17.4 Hz, 1.28 Hz, 1H, –CH = C**H_2_**), 4.53 (brs, 1H, –C = C**H_2_**), 4.29 (t, 1H, HO–C**H**–), 4.16 (brs, 1H, –C = C**H_2_**), 2.30–0.8 (m, 14H), 1.23 (s, 3H, –C**H_3_**), 1.17 (s, 3H, –C**H_3_**), 0.60 (s, 3H, –C**H_3_**). ^13^C NMR (APT 151 MHz, CDCl_3_) *δ* 181.5 (–**C**OOH), 150.9 (–**C**=CH_2_), 146.6 (–**C**H = CH_2_), 111.9 (–CH=**C**H_2_), 109.7 (–C=**C**H_2_), 74.8 (**C–**OH), 74.2 (**C–**OH), 51.6, 49.7, 44.8, 42.1, 41.6, 40.3, 39.4, 33.9, 29.3 (–**C**H_3_), 27.5 (–**C**H_3_), 20.2, 17.6, 12.4 (–**C**H_3_). MS (ESI), *m/z* for C_20_H_32_O_4_, Calc 336.4720, Found 335.2 [M − H]^-^; Elemental Analysis: C, 71.39; H, 9.59; O, 19.02, Found C, 71.44; H, 9.48; O, 19.20.

### Biological assay

#### Effect of cytotoxicity of the tested compounds against RAW264.7 macrophage cells

RAW264.7 is a known model for the *in vitro* investigation of anti-inflammatory activity^15^. To inspect the safety of the investigated derivatives (**1**–**10**) on RAW264.7 macrophage cells, a cytotoxicity study was performed. WST-1 reagent was used to detect the viability of the treated cells. The derivatives were toxic to RAW264.7 at 100 and 50 µg/mL, while more viable cells were observed at 25 µM. Therefore, all the experiments were performed at a safe dose to the cells (i.e., 25 µM).

#### Modulation of COX-2 expression in RAW264.7 cells

**Figure 4.**
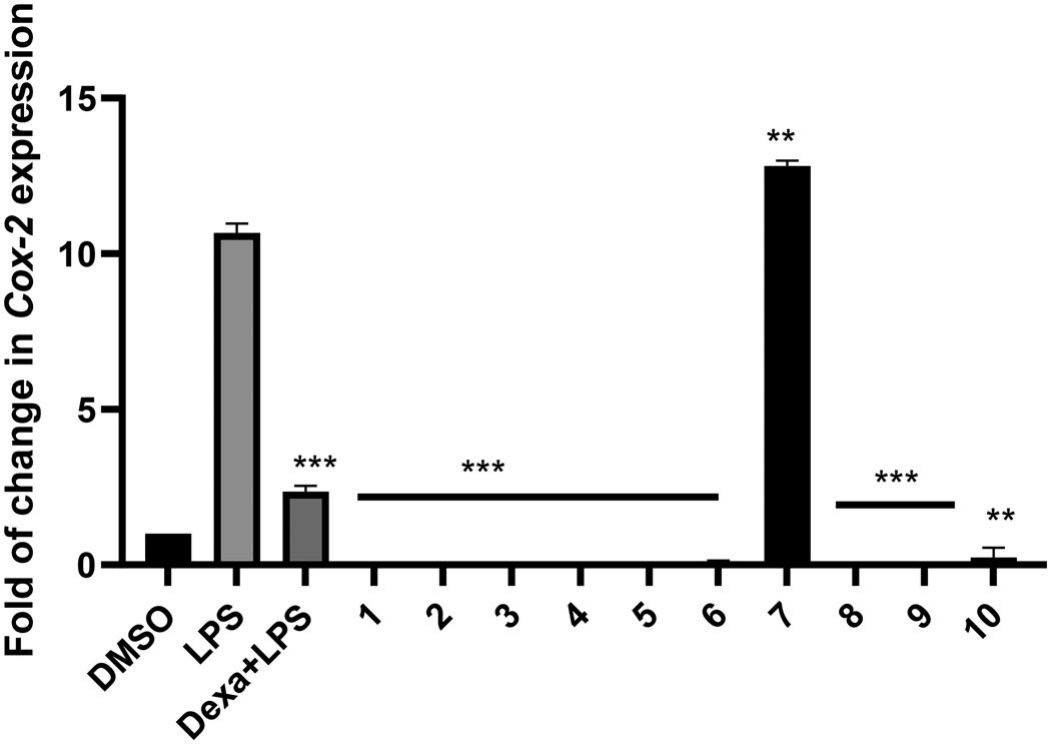
Modulation of *COX-2* expression in RAW264.7 cells pre-treated with tested compounds. The fold of change of *COX-2* gene was calculated after normalisation to β-actin. The statistical analysis was performed by One-Way ANOVA by using GraphPad Prism Version 8.0. *** means *p* values =0.0009 while ** means *p* values = 0.003.

Based on the cytotoxicity assay which showed that the investigated compounds were safe to RAW264.7 cells at 25 µg/mL, the anti-inflammatory activity was evaluated. LPS is an external cell stimulus for immune cells via binding with Toll-Like receptor 4 ^17^. Stimulation with LPS produces inflammatory cytokines, such as IL-6 and COX-2 enzyme. In this study, LPS treatment led to a change in the macrophage morphology (elongated and spindle shape) while the pre-treatment of macrophage cells either with compounds **1, 2, 3, 4, 5, 6, 8, 9,** or **10** or Dexamethasone inhibited the response to LPS and decreased the inflammation (Figure 4). The RAW264.7 cells treated with LPS alone expressed a high level of the inflammatory gene *COX-2*, while pre-treatment of the cells for 3h with the tested compounds (except compound **7**) showed downregulation of *COX-2* expression in response to LPS. The results were comparable to the standard anti-inflammatory drug, Dexamethasone (Figure 4). In contrast, compound **7** which is characterised by the presence of a hydrophobic hexyl chain at C-19 of torulosic acid, stimulated COX-2 expression. The obtained results were in full agreement with the previously published reports which demonstrated significant anti-inflammatory activity of different diterpene skeletons on LPS-induced RAW264.7 cells by various mechanisms, including the downregulation of *COX-2* gene expression^15–17^. The first labdane-type diterpene to be discovered as an anti-inflammatory agent was *cis*-communic acid isolated from the leaves of *Cryptomeria japonica* (Taxodiaceae). It was identified as the main biomolecule responsible for the activity of a topical traditionally used Japanese preparation for the treatment of skin eruption, swelling, eczema, and injury. *Cis*-communic acid was shown to inhibit carrageenan-induced paw edoema in rats and histamine-induced contraction in guinea pig ileum^18^^,^^19^.

#### Determination of the level of IL-6 by ELISA

**Figure 5.**
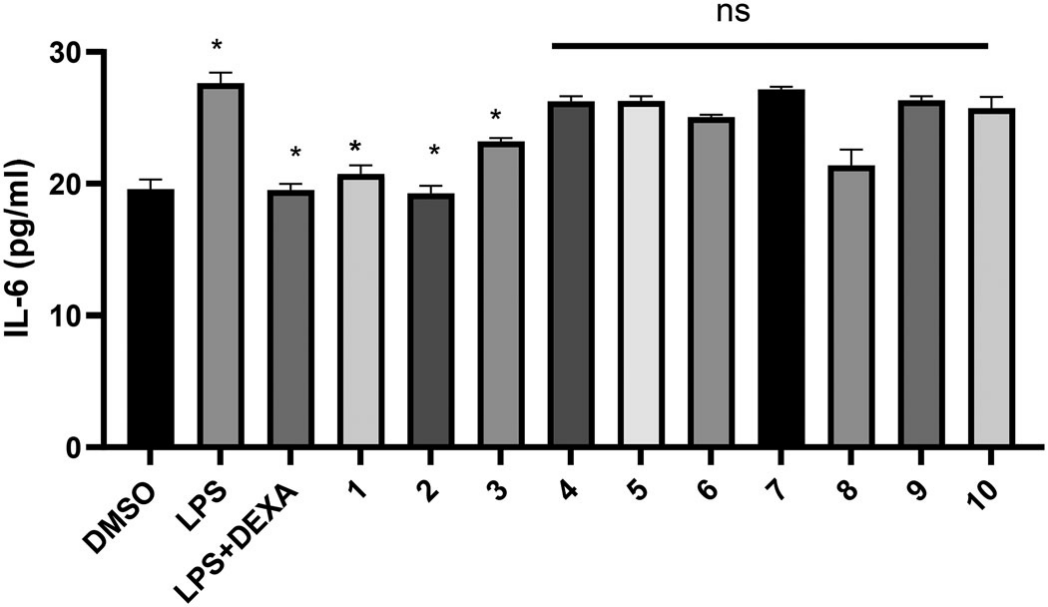
Attenuation of the IL-6 level by the pre-treatment of Raw264.7 cells with the compounds 1, 2 or 3. The level of IL-6 has been statistically calculated by using GraphPad Prism Version 8.0 in comparison to the LPS-treated cells. * Means *p* values <0.05.

Recognition of LPS by macrophage cells leads to the activation of downstream signalling pathways and transcription factors, such as Nuclear Factor-κB (NF-κB)^20^ and Activated Protein-1 (AP-1)^21^. The activated transcription factors induce the expression of different cytokines, including IL-1, IL-6, and TNF-α. The anti-inflammatory activity of the compounds was further confirmed by measuring the level of IL-6. To investigate the effect of derivatives on pro-inflammatory cytokines, the level of IL-6 was measured in the medium of RAW264.7 cells after treatment either with LPS alone or pre-treatment with the studied compounds for 3h, followed by treatment with LPS for 21h (Table 2 and Figure 5). LPS treatment dramatically enhanced the expression of *IL-6*. Analysis of the results revealed that pre-treatment with compounds **1**, **2**, or **3** significantly attenuated the LPS stimulation and reduced the level of IL-6 (*p* < 0.05). It has been documented that IL-6 plays a major role in the induction of septic shock via releasing prostaglandins and leukotrienes and activating signal transducer and activator of transcription 3STAT3^22^. The bioactive derivatives **1**–**3** shared common structural features, including the occurrence of terminal hydroxyl groups in the substituents at C-19 of torulosic acid, in addition to the absence of the hydroxyl group at C-7. This report described for the first time the effect of 13-epi-cupressic acid (Torulosic acid) and its derivatives on the downregulation of inflammatory cytokines expression, such as IL-6. However, several studies found in the literature demonstrated a similar effect exhibited by other diterpene classes^23–25^.

**Table 2.** IL-6 level measured in the medium of LPS-induced RAW264.7 cells.

Treatment	IL-6 (pg/mL)	SD*
DMSO	19.6	0.72
LPS	27.6	0.81
LPS + DEXA	19.5	0.45
**1**	20.73	0.723
**2**	19.3	0.7920
**3**	23.2	0.4425
**4**	26.3	0.369
**5**	26.3	0.35
**6**	25.1	0.19
**7**	27.2	0.20
**8**	21.4	1.20
**9**	26.3	0.30
**10**	25.7	0.86

*SD is the standard deviation from three independent replicates.

Previous reports showed that labdane diterpenoids may exert their anti-inflammatory action through multiple mechanisms mainly found at the levels of gene expression and/or enzyme activity, such as interfering with the NF-κB signalling pathway, NO production, or arachidonic acid (AA) metabolism^26^. Forskolin andrographolide, sclareol, and (+)-polyalthic acid are examples of labdane diterpenoids reported in the literature as potential anti-inflammatory constituents from several successful traditional remedies^26^. Our results demonstrated that most of the prepared derivatives were able to downregulate *COX-2* gene expression and attenuate *IL-6* level in LPS-stimulated RAW264.7 cells. In conclusion, our findings suggested 13-epi-cuppressic acid and its derivatives as promising leads for the development of new anti-inflammatory drugs. However, further *in vivo* experiments are required to study their pharmacokinetic and toxicological properties.

### Docking experiments

Docking experiments were performed using MOE software. The docking results of the prepared derivatives (**1**–**10**) against the active binding pocket of PLA2 are displayed in Table 3. It demonstrated that the greatest docking score was recorded by derivative **10** (−8.97 kcal/mol), which showed greater binding affinity than that of the co-crystallized inhibitor, BR4 (−8.01 kcal/mol). These are followed by the starting compound **1** (−7.87 kcal/mol), and derivatives **2** (−7.86 kcal/mol). Whereas, the lowest binding affinity was recorded for **7** (−6.34 kcal/mol), followed by **9** (−6.72). The binding affinities are in full agreement with the obtained *in vitro* assays as compound **7** was not able to downregulate *COX-2* gene expression. On the other hand, derivatives **1** and **2** were among the most active derivatives in both Leukocyte accumulation and IL-6 assays.

**Table 3.** Docking interactions of the prepared derivatives **(1**–**10)** against phospholipase A2 (PLA2) enzyme.

Derivative code	Phospholipase A2			
	Docking scores (kcal/mol)	RMSD	Types of interactions	
			Metal complex	Amino acid residues
**1**	−7.87	1.01	Ca:301	Gly31, Lys62
**2**	−7.86	1.73	Ca:301	Gly31
**3**	−7.05	2.08	–	Leu2
**4**	−7.00	1.88	Ca:301	Gly29
**5**	−7.25	1.28	Ca:301	Gly29, Gly31, Lys62
**6**	−7.37	1.19	Ca:301	–
**7**	−6.34	1.50	–	–
**8**	−7.36	1.33	Ca:301	Cys44
**9**	−6.72	1.61	Ca:301	Gly31, Lys52
**10**	−8.97	1.61	Ca:301 (x 2)	Gly29, Lys62
BR4^a^	−8.01	1.42	Ca:301	Gly29, Gly31, His47
Dexamethasone	−5.81	1.42	–	–

^a^BR4: co-crystallized ligand; 6-phenyl-4(*R*)-(7-phenyl-heptanoylamino)-hexanoic acid in the complex with the active site of phospholipase A2 (PLA2), PDB code: 1KQU^11^.

PLA2 inhibitors are mainly characterised by their ability to form electrostatic interactions with Ca^+2^ ion, H-bonding with the catalytic amino acid, His47, and various types of hydrophobic binding interactions with the hydrophobic residues lining the active site of the enzyme^27^. The structure of co-crystallized ligand (BR4) was docked within the investigated set of derivatives for validating the docking results, where it showed remarkable binding affinity (−8.01 kcal/mol) and the same interactions as the crystal structure, including Ca:301 (ion contact), Gly29 (H-bond and arene-H), Gly31 (H-bond), and His47 (H-bond) (Figure S47). However, Dexamethasone showed the lowest binding affinity (−5.81 kcal/mol) and no binding interactions (Figure S3).

Visualisation of the docked structures revealed that all compounds showed interactions with the crucial Ca^+2^ metal ion (Ca:301) except for **3** and **7** (Figure S49 and S50). Also, all compounds did not show interaction with His47 residue. However, most of them showed H-bonding interactions with Gly29, Gly31, and Lys52^11^^,^^27^.

**Figure 6.**
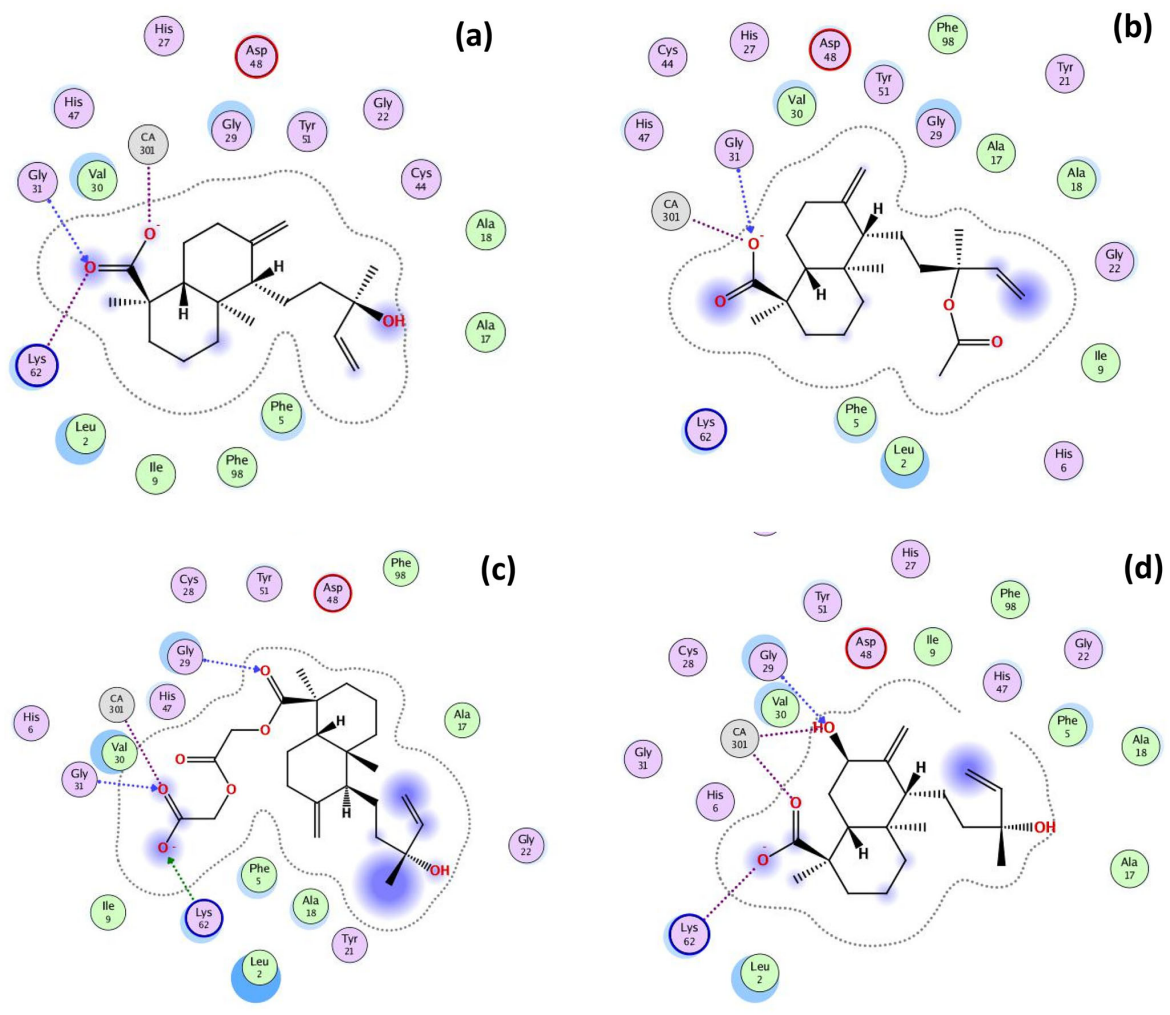
2D binding modes of interaction of; a) compound **1**, (b) compound **2**, (c) compound **5**, and (d) compound **10** with the active site of phospholipase A2 (PDB code: 1KQU).

Compound **1** showed binding interactions with the Ca:301 (metal contact), residues Gly31 (H-bond), and Lys62 (ion contact) through the COOH group at C-4 (Figure 6(a)). Similarly, **2** interacted with Ca:301 and Gly31 via the COOH group at C-4 (Figure 6(b)). The aliphatic ester derivative **5** showed a remarkable capacity to form several interactions mainly via the introduced ester group at C-4, which was able to extend the molecules to interact with Ca:301 (metal complex), Glys31, and Lys62 (H-bonding), in addition to the parent’s carbonyl at C-4 that showed H-bonding with Gly29 (Figure 6(c)). Extended conformation was found to be required for optimal PLA2 inhibitory activity for filling the hydrophobic active site of the enzyme^11^. Remarkably, derivative **10** showed the top score (−8.97 kcal/mol) due to various interactions with the active site of PLA2. These interactions include two Ca:301 metal contacts with both the COOH group at C-4 and the OH group at C-7. Moreover, it exhibited H-bonding interaction with Gly29 through the OH group at C-7 (Figure 6(d)).

The aromatic ester derivative **9** similarly showed interactions with the parent’s carbonyl at C-4 with both Ca:301 (metal contact) and Gly31 (H-bond). But it was able to form an arene-H interaction with Lys52 through the introduced benzyl moiety. The aliphatic ester derivative **6** only showed a metal ion interaction with Ca:301 through its OH group at C-13. Compound **3** also showed one interaction with the hydrophobic amino acid Leu2 through H-bonding with the introduced ethanolamide moiety. However, the less polar hexyl derivative **7** did not show any interactions, which can be further explained by its lowest binding affinity (−6.34 kcal/mol). This result agreed with the obtained *in vitro* anti-inflammatory activity for this derivative. The isobutyl ester **8** showed H-bonding with Cyst44 via the OH group at C-13, and the ester group at C-4 formed a metal complex with Ca:301. Finally, derivative **4** showed a metal contact with Ca:301 and H-bonding with Gly29 residue through the parent’s carbonyl group.

**Figure 7.**
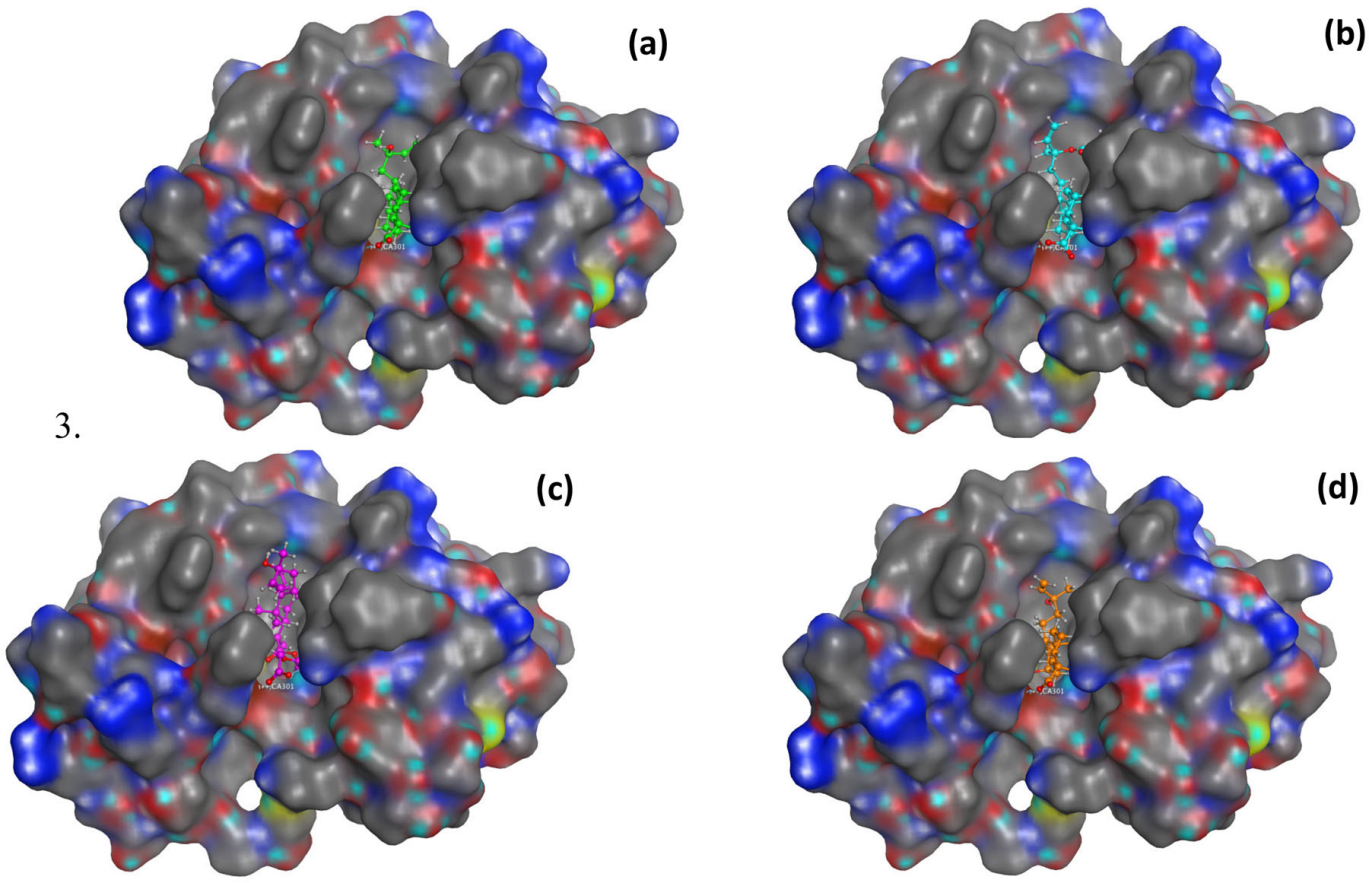
Molecular surface interactions of the docked structures of; a) compound **1**, (b) compound **2**, (c) compound **5**, and (d) compound **10** with the active site of phospholipase A2 (PDB code: 1KQU).

It could be concluded that the most important structural features required for the activity of 13-epi-cupressic acid derivatives as PLA2 inhibitors include the presence of carbonyl group at C-4 which is crucial for binding to Ca^+2^ metal ion as was observed in most compounds (Table 3). Additionally, the hydrophobic diterpenoid skeleton is essential for filling the hydrophobic part of the active site (Figure 7). Also, the presence of aliphatic substitution at COOH (C-19) is more favourable than aromatic ones, although free unsubstituted COOH showed better binding affinities and *in vitro* anti-inflammatory activity as observed in **1**, **2**, and **10**.

### Physicochemical and ADME predictions

The physicochemical, ADME, and drug-likeness properties of the prepared derivatives (**1**–**10**) were *in silico* investigated using the SwissADME online software (Swiss Institute of Bioinformatics; http://www.sib.swiss)^12^. The results (Table S3) showed conformity with the Lipinski’s rules. However, one violation was recorded for compounds **4**, and **7**–**9** due to their elevated lipophilicity (MLOGP: Moriguchi octanol-water partition coefficient >4.15),^28^. Likewise, none of the prepared derivatives showed PAINS alerts (Pan-assay interference compounds). Additionally, all derivatives showed high GIT absorption as indicated by the predicted values’ range of 0.55-0.85, indicating oral bioavailability. Also, no possible inhibitions were recorded for any of the investigated compounds against CYP450-1A2 indicating that they are safe and less likely to affect the metabolism of drugs^29^. The blood-brain barrier (BBB) represents an obstacle in the delivery of drugs to the brain, derivatives **1–3**, **8**, and **10** were able to cross BBB but **4**–**7** and **9** were not^30^. The P-glycoprotein (P-gp) contributes to multidrug resistance to several drugs through the efflux of drugs back into the blood^31^. Compounds **3**–**5** and **10** may show practical resistance based on the predicted results. Radar map is an interpretation of the average values of six descriptors applied for the quick assessment of drug-likeness properties. These descriptors include the size of the molecule, lipophilicity, polarity, flexibility, saturation, and solubility (Figure S51). The synthesized derivatives were found to fall inside the pink area of their corresponding radar plots^12^. Interestingly, the bioactive compounds **1**, **2**, and **10** were located entirely inside this pink area which indicated that they have the properties of drugs.

## Conclusions

Diterpenes, including the labdane group, have been reported to possess both *in vitro* and *in vivo* anti-inflammatory activities. In this study, we prepared a series of semisynthetic derivatives based on the naturally occurring labdane diterpenoids namely, 13-epi-cupressic and acetyl-13-epi-cupressic acids obtained from the trunk of *Araucaria heterophylla* (Salisb.) Franco trees. The chemical reactions used in the preparation of eight derivatives (**3**–**10**) from the starting molecules were described, including esterification, amidation, and allylic hydroxylation reactions. The anti-inflammatory activity of compounds was evaluated in RAW264.7 cells *via* measuring the level of *COX-2* expression, and level of IL-6. The results showed that most of the prepared derivative exhibited significant (*P* < 0.05) anti-inflammatory activities as revealed from downregulation of *COX-2* expression, diminished IL-6 level of LPS-induced inflammation in RAW264.7 cells. An *in silico* docking experiment on phospholipase A2 (PLA2), a vital enzyme in starting the inflammatory cascade, revealed the importance of the carbonyl group at C-4, (free or substituted with aliphatic chains) and the hydrophobic diterpenoid skeleton. This study suggested 13-epi-cupressic acid as a scaffold for the synthesis of new anti-inflammatory agents. The current study proposed 13-epi-cupressic acid as a promising natural lead molecules for the discovery and development of new anti-inflammatory drugs.

## Supplementary Material

Supplemental Material
