# Supplementary material for: Design, biological evaluation, and molecular modelling insights of cupressic acid derivatives as promising anti-inflammatory agents
Source: J Enzyme Inhib Med Chem. 2023 Mar 13;38(1):2187327. doi: 10.1080/14756366.2023.2187327 (PMC10013213; doi:10.1080/14756366.2023.2187327)
Supplement: Supplemental Material [file IENZ_A_2187327_SM0675.pdf]

Supplementary Material

Design, Biological Evaluation, and Molecular Modelling Insights of Cupressic Acid Derivatives as Promising Anti-Inflammatory Agents

Amal F. Soliman[a*], Diaaeldin M. Elimam[b], Fardous F El-Senduny[c,d], Manal A. Alossaimi[e], Mubarak Alamri[e], Fatma M. Abdel Bar[f,g*]

[a] Department of Pharmacognosy, Faculty of Pharmacy, Mansoura University, Mansoura 35516, Egypt; [b] Department of Pharmacognosy, Faculty of Pharmacy, Kafrelsheikh University, Kafrelsheikh, 33516, Egypt; [c] Faculty of Science, Chemistry Department, Biochemistry Division, Mansoura University, Mansoura 35516, Egypt; [d] Department of Pathology & Laboratory Medicine, Sylvester Comprehensive Cancer Center, Miller School of Medicine, Miami, FL, 33136, United States; [e] Department of Pharmaceutical Chemistry, College of Pharmacy, Prince Sattam Bin Abdulaziz University, Al-Kharj 11942, Saudi Arabia; [f] Department of Pharmacognosy, College of Pharmacy, Prince Sattam Bin Abdulaziz University, Al-Kharj, 11942, Saudi Arabia; [g] Faculty of Pharmacy, Mansoura University, Mansoura 35516, Egypt

[*]Corresponding authors:

1) Amal F. Soliman: amalsoliman134@yahoo.com; amalsoliman134@mans.edu.eg

2) Fatma M. Abdel Bar: f.abdelbar@psau.edu.sa; fatma_maar@yahoo.com

Co-authors E-mails:

Diaaeldin M. Elimam: dr_deya@yahoo.com; Dr_deya@pharm.kfs.edu.eg

Fardous F. El-Senduny: fxe123@miami.edu, fkaneer@mans.edu.eg

Manal A. Alossaimi: m.alossaimi@psau.edu.sa

Mubarak Alamri: m.alamri@psau.edu.sa

ORCID

Amal F. Soliman: https://orcid.org/0000-0003-3320-0685

Diaaeldin M. Elimam: https://orcid.org/0000-0003-4661-6081

Fardous F. El-Senduny: https://orcid.org/0000-0002-4572-2061

Manal A. Alossaimi: https://orcid.org/0000-0001-8911-8109

Fatma M. Abdel Bar: https://orcid.org/0000-0003-0823-4015

Table of Contents	Page
**Table S1.** ^{13}C NMR spectral data of derivatives (**1-10**).	3
**Table S2.** ^{1}H NMR spectral data of derivatives (**1-10**).	4
**Table S3.** The physicochemical, ADME, and druglikeness properties of compounds (**1-10**) using the SwissADME online tool.	52
**Figure S1.** IR spectrum of compound **1**.	5
**Figure S2.** a) Full and b) selected expansions of ^{1}H NMR spectrum compound **1**.	6
**Figure S3.** ^{13}C NMR and APT of compound **1**.	7
**Figure S4.** Mass spectroscopy of compound **1**.	8
**Figure S5.** IR spectrum of compound **2**.	9
**Figure S6.** ^{1}H NMR spectrum of compound **2**.	9
**Figure S7.** DEPT spectrum of compound **2**.	10
**Figure S8.** Mass spectroscopy of compound **2**.	11
**Figure S9.** ^{1}H NMR spectrum of compound **3**.	12
**Figure S10.** APT spectrum of compound **3**.	13
**Figure S11.** Mass spectroscopy of compound **3**.	14
**Figure S12.** IR spectrum of compound **4**.	14
**Figure S13.** ^{1}H NMR spectrum of compound **4**.	15
**Figure S14.** ^{13}C NMR of compound **4**.	16
**Figure S15.** APT spectrum of compound **4**.	17
**Figure S16.** Mass spectroscopy of compound **4**.	18
**Figure S17.** ^{1}H NMR spectrum of compound **5**.	19
**Figure S18.** APT spectrum of compound **5**.	20
**Figure S19.** Mass spectroscopy of compound **5**.	21
**Figure S20.** IR spectrum of compound **6**.	22
**Figure S21.** ^{1}H NMR spectrum of compound **6**.	23
**Figure S22.** APT spectrum of compound **6**.	24
**Figure S23.** Mass spectroscopy of compound **6**.	24
**Figure S24.** IR spectrum of compound **7**.	25
**Figure S25.** ^{1}H NMR spectrum of compound **7**.	26
**Figure S26.** ^{1}H NMR expansion of compound **7**.	27
**Figure S27.** APT spectrum of compound **7**.	28
**Figure S28.** APT spectrum expansion of compound **7**.	29
**Figure S29.** Mass spectroscopy of compound **7**.	30
**Figure S30.** IR spectrum of compound **8**.	31
**Figure S31.** a) Full and b) selected expansions of ^{1}H NMR spectrum of compound **8**.	32
**Figure S32.** APT spectrum of compound **8**.	33
**Figure S33.** Mass spectroscopy of compound **8**.	34
**Figure S34.** IR spectrum of compound **9**.	35
**Figure S35.** ^{1}H NMR spectrum of compound **9**.	36
**Figure S36.** APT spectrum of compound **9**.	37
**Figure S37.** Mass spectroscopy of compound **9**.	38
**Figure S38.** IR spectrum of compound **10**.	39
**Figure S39.** ^{1}H NMR spectrum of compound **10**.	40
**Figure S40.** ^{13}C NMR spectrum of compound **10**.	41
**Figure S41.** APT spectrum of compound **10**.	42
**Figure S42.** HSQC spectrum of compound **10**.	43
**Figure S43.** HMBC spectrum of compound **10**.	43
**Figure S44.** NOESY spectrum of compound **10**.	44
**Figure S45.** Mass spectrum of compound **10**.	45
**Figure S46.** Structures of the prepared cupressic acid derivatives (**1-10**).	46
**Figure S47.** 2D binding modes of interaction of the inhibitor, **BR4** (6-phenyl-4(*R*)-(7-phenyl-heptanoylamino)-hexanoic acid); a) Co-crystallised and (b) docked structure within the active site of phospholipase A2 (PDB code: 1KQU) indicated method validation.	47
**Figure S48.** 3D binding modes of interaction of; a) compound **1**, (b) compound **2**, (c) compound **5**, and (d) compound **10** with the active site of phospholipase A2 (PDB code: 1KQU).	48
**Figure S49.** 2D and 3D binding modes of interaction of; (a, b) Dexamethasone, (c, d) compound **3**, (e, f) compound **6** with the active site of phospholipase A2 (PDB code: 1KQU).	49
**Figure S50.** 2D and 3D binding modes of interaction of; (a, b) Compound **7**, (c, d) compound **8**, (e, f) compound **9** with the active site of phospholipase A2 (PDB code: 1KQU).	50
**Figure S51.** Bioavailability radar figures of the prepared analogues **1-10** (a-j) respectively, using SwissADME online tool (Swiss Institute of Bioinformatics; http://www.sib.swiss)	51

Table S1. ^{13}C NMR spectral data of derivatives (**1-10**) at [a]100, [b]125, or [c]150 MHz. * In $CDCl_3$ or ** In CD_3OD.

	**1***[a]	**2***[a]	**3****[c]	**4***[b]	**5***[a]	**6***[a]	**7***[a]	**8***[a]	**9***[a]	**10***[c]
**C**	**DEPT**	**DEPT**	**APT**	**DEPT**	**APT**	**APT**	**APT**	**APT**	**APT**	**APT**
1	39.2 CH_2	38.0 CH_2	39.1 CH_2	39.6 CH_2	39.1 CH_2	38.8 CH_2	39.2 CH_2	39.4 CH_2	39.1 CH_2	40.3 CH_2
2	19.9 CH_2	19.9 CH_2	21.3 CH_2	20.2 CH_2	19.9 CH_2	20.0 CH_2	19.9 CH_2	20.7 CH_2	20.0 CH_2	20.2 CH_2
3	38.0 CH_2	36.9 CH_2	37.8 CH_2	38.4 CH_2	38.4 CH_2	38.4 CH_2	38.2 CH_2	38.5 CH_2	38.2 CH_2	39.4 CH_2
4	44.2 C	44.2 C , 44.2	45.3 C	43.1 C, 44.2	44.5 C, 44.2	44.5 C	44.3 C	44.4 C	44.4 C	44.8 C
5	56.4 CH	56.4 CH	58.2 CH	56.5 CH	56.3 CH	56.6 CH	56.4 CH	56.6 CH	56.5 CH	49.7 CH
6	26.0 CH_2	26.0 CH_2	24.6 CH_2	26.4 CH_2	26.0 CH_2	27.9 CH_2	26.2 CH_2	26.3 CH_2	26.7 CH_2	33.9 CH_2
7	38.4 CH_2	38.7 CH_2	38.2 CH_2	38.7 CH_2	38.7 CH_2	39.2 CH_2	38.8 CH	39.1 CH_2	38.7 CH_2	74.8 CH
8	148.0 C	148.0 C	148.9 C	147.7 C	147.9 C	148.2 C	147.8 C	148.0 C	148.0 C	150.9 C
9	56.7 CH	56.6 CH	58.3 CH	56.3 CH	56.5 CH	56.5 CH	56.5 CH	56.8 CH	56.5 CH	51.6 CH
10	40.7 C	39.2 C	40.1 C	39.5 C	40.5 C	40.4 C	40.5 C	40.5 C	40.5 C	42.1C
11	17.9 CH_2	17.6 CH_2	18.9 CH_2	23.3 CH_2	17.9 CH_2	18.0 CH_2	17.9 CH_2	18.0 CH_2	18.0 CH_2	17.6 CH_2
12	41.5 CH_2	40.6 CH_2	40.5 CH_2	134.0 CH	41.4 CH_2	41.5 CH_2	41.4 CH_2	41.5 CH_2	41.4 CH_2	41.6 CH_2
13	73.6 C	83.3 C	84.9 C	133.5 C	73.5 C	73.4 C	73.4 C	73.4 C	73.5 C	74.2 C
14	145.0 CH	141.9 CH	143.2 CH	141.5 CH	145.2 CH	147.9 CH	145.2 CH	145.3 CH	145.2 CH	146.6 CH
15	111.5 CH_2	113.0 CH_2	113.5 CH_2	109.8 CH_2	111.6 CH_2	111.5 CH_2	111.5 CH_2	111.5 CH_2	111.6 CH_2	111.9 CH_2
16	29.2 CH_3	23.5 CH_3	22.9 CH_3	11.9 CH_3	28.9 CH_3	22.8 CH_3	28.9 CH_3	29.5 CH_3	29.7 CH_3	29.3 CH_3
17	106.6 CH_2	106.6 CH_2	106.8 CH_2	107.7 CH_2	106.7 CH_2	106.8 CH_2	106.6 CH_2	106.7 CH_2	106.7 CH_2	109.7 CH_2
18	27.6 CH_3	28.9 CH_3	30.4 CH_3	30.2 CH_3	27.6 CH_3	27.8 CH_3	27.5 CH_3	27.6 CH_3	27.5 CH_3	27.5 CH_3
19	183.0 C	183.2 C	171.9 C	176.5 C	176.6 C	176.5 C	177.3 C	177.2 C	177.1 C	181.5 C
20	12.7 CH_3	12.7 CH_3	12.5 CH_3	13.0 CH_3	12.8 CH_3	12.9 CH_3	12.7 CH_3	12.7 CH_3	12.7 CH_3	12.4 CH_3
OCO<u>CH</u>$_3$		22.1 CH_3	20.9 CH_3							
O<u>C</u>OCH_3		169.8 C	169.3 C							
Substituents at C19 1’			42.8 CH_2	40.6 CH_2	60.8 CH_2	61.2 CH_2	64.2 CH_2	70.5 CH_2	66.0 CH_2	
2’			61.5 CH_2	35.3 CH_2	167.6 C	167.8 C	28.3 CH_2	27.6 CH		
3’					60.0 CH_2	60.4 CH_2	25.7 CH_2	19.3 CH_3		
4’					167.4 C	14.13 CH_3	31.3 CH_2	19.4 CH_3		
5’							22.5 CH_2			
6’							14.0 CH_3			
Aromatic ring 1’’				138.9 C					136.0 C	
2’’				128.6 CH					128.0 CH	
3’’									128.5 CH	
4’’				126.4 CH					126.9 CH	
5’’				128.6 CH					128.1 CH	
6’’									127.4 CH	

Table S2. ^{1}H NMR spectral data of derivatives (**1-10**) at [a]400, [b]500, or [c]600 MHz. * In $CDCl_3$ or ** In CD_3OD.

^{1}H NMR		1*[a]	2*[a]	3**[c]	4*[b]	5*[a]	6*[a]	7*[a]	8*[a]	9*[a]	10*[c]
1		1.10m<br>1.87m	1.16m<br>1.77m	1.16m<br>1.60m	1.09m<br>overlapped<br>1.72m	1.13m<br>1.74m	1.16m<br>1.86m	1.10m<br>1.78m	1.15m<br>1.91m	1.11m<br>1.76m	1.15m<br>1.83m
2		1.55m<br>1.90m	1.53m<br>1.92m	1.56m<br>1.88m	1.51m<br>1.90m	1.50m<br>1.89m	1.47<br>overlapped<br>1.94m	1.51m<br>1.90m	1.58m<br>1.91m	1.55m<br>1.91m	1.50m<br>1.89m
3		0.9m<br>2.18 br.d<br>(12)	0.78m<br>2.11 br.d<br>(12)	0.85m<br>2.19 br.d<br>(12)	0.88m<br>2.10 br.d (12)	0.85m<br>2.18 br.d<br>(12)	0.84m<br>2.30 br.d (12)	0.98m<br>2.18 br.d<br>(12)	0.98m<br>2.28 br.d<br>(12)	2.21 br.d<br>(12)	2.11 br.d<br>(12)
4		---------	---------	---------	---------	---------	---------	---------	---------	---------	---------
5		1.78 t	1.80 t	1.90 t	1.75 t	1.78 m	1.86 m	1.85 t	1.90 m	1.84 t	1.81 t<br>overlapped
6		1.07m<br>1.40m	1.16m<br>overlapped<br>1.45<br>overlapped	1.17<br>Overlapped<br>1.50<br>overlapped	1.17<br>overlapped<br>1.51<br>overlapped	1.16m<br>1.44m	1.16m<br>1.40m	1.18 m<br>overlapped<br>1.45m	1.1-1.5<br>overlapped	1.11-1.5<br>overlapped	1.84m<br>2.08m
7		1.91<br>overlapped<br>2.01m	1.92<br>overlapped<br>2.08m	1.98m<br>2.01m	1.90m<br>2.00m	1.88m	1.98m<br>2.10m	1.90m	1.91m<br>2.08m	1.91m<br>2.10m	4.29 t (3)
8		--------	--------	--------	--------	--------	--------	--------	--------	--------	--------
9		2.20m	2.32m	2.19m	2.30m	2.32m	2.32m	2.38m	2.25m	2.39m	2.12m
10		--------	--------	--------	--------	--------	--------	--------	--------	--------	--------
11		1.38m<br>1.58m	1.45m<br>1.77m	1.45-170<br>overlapped	2.33 m<br>1.86m	1.28m<br>1.54m	1.39m<br>1.62m	1.29m<br>1.67m	1.29m<br>1.61m	1.31m<br>1.56m	1.36m<br>1.61m
12		1.32m<br>1.74m	1.53 m<br>1.78 m	1.50m<br>1.88m	5.36 t	1.24m<br>1.74m	1.24m<br>1.80m	1.24m<br>1.74m	1.33m<br>1.93m	1.31m<br>1.76m	1.28m<br>1.73m
13		--------	--------	--------	--------	--------	--------	--------	--------	--------	--------
14		5.95 dd<br>(12,16)	5.89 dd<br>(12,16)	5.95 dd<br>(12,16)	6.33 dd<br>(12,17)	5.84 dd<br>(12,16)	5.90 dd (10.8,<br>17.4)	5.83 dd<br>(10.8, 17.4)	5.95 dd<br>(12,16)	5.95 dd<br>(12,16)	5.90 dd<br>(10.8, 17.4)
15		5.08 d<br>(16), 5.24<br>d (12)	5.02 d (16)<br>5.04 d (12)	5.08 d<br>(16), 5.14<br>d (12)	5.04 d (17)<br>5.20 d (12)	4.99 d (16)<br>5.10 d (12)	5.10 dd (1.2,<br>17.4)<br>5.25 dd (1.2,<br>10.8)	4.95 dd<br>(1.8, 17.4)<br>5.10 dd<br>(1.8, 10.8)	5.08 d (16),<br>5.24 d (12)	5.08 d (16),<br>5.24 d (12)	5.01 dd<br>(1.8, 17.4)<br>5.18 dd<br>(1.8, 10.8)
16		1.30 s	1.45 s	1.30 s	1.75 s	1.45 s	1.17 s	1.17 s	1.30 s	1.30 s	1.17 s
17		4.56 s<br>4.87 s	4.44 s<br>4.77 s	4.56 s<br>4.87 s	4.43 s<br>4.80 s	4.45 s<br>4.77 s	4.63 brs<br>4.93 brs	4.45 brs<br>4.76 brs	4.56 s<br>4.87 s	4.56 s<br>4.87 s	4.16 brs<br>4.53 brs
18		1.26 s	1.16 s	1.26 s	1.09 s	1.16 s	1.19 s	1.23 s	1.26 s	1.26 s	1.23 s
19		--------	--------	--------	--------	--------	---------	------------	--------	--------	------------
20		0.63 s	0.53 s	0.63 s	0.53 s	0.53 s	0.60 s	0.45 s	0.63 s	0.63 s	0.60 s
OCOCH3			1.96 s	1.96 s							
Substituents at C19	1'			3.26 m	3.48 m	4.54 d (16)<br>4.68 d (16)	4.65 d (15.7)<br>4.52 d (15.7)	3.92 m	3.78 dd (6.2,<br>10.6)<br>3.89 dd (6.2,<br>10.6)	4.67 s	
	2'			3.56 t	2.80 t	-------------	--------------	1.65 m	1.91 m		
	3'					4.61 d (2)	4.25 dd (7.2,<br>14.3)		0.97 d (2.4)		
	4'					------------	1.20 t<br>(overlapped)	1.28-1.57 m	0.99 d (2.4)		
	5'										
	6'							0.81 t (6.9)			
Aromatic ring	2''- 6''				7.21-7.46 m					7.34-7.37 m	

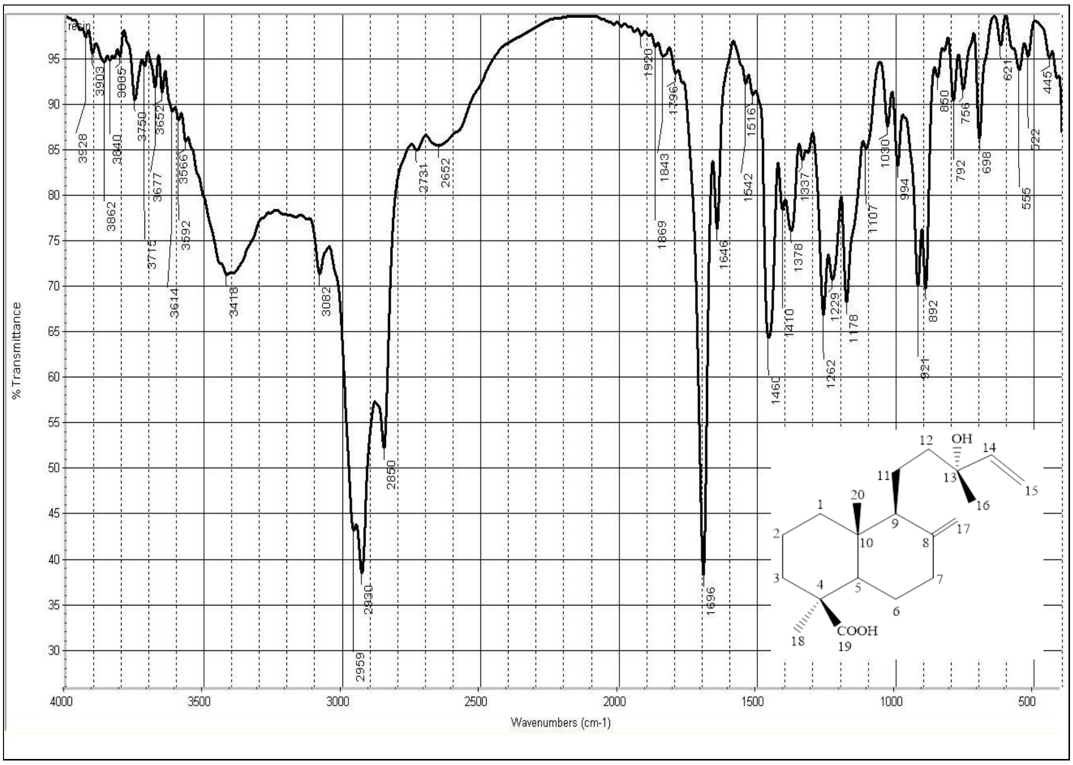

Figure S1. IR spectrum of compound **1**.

a)

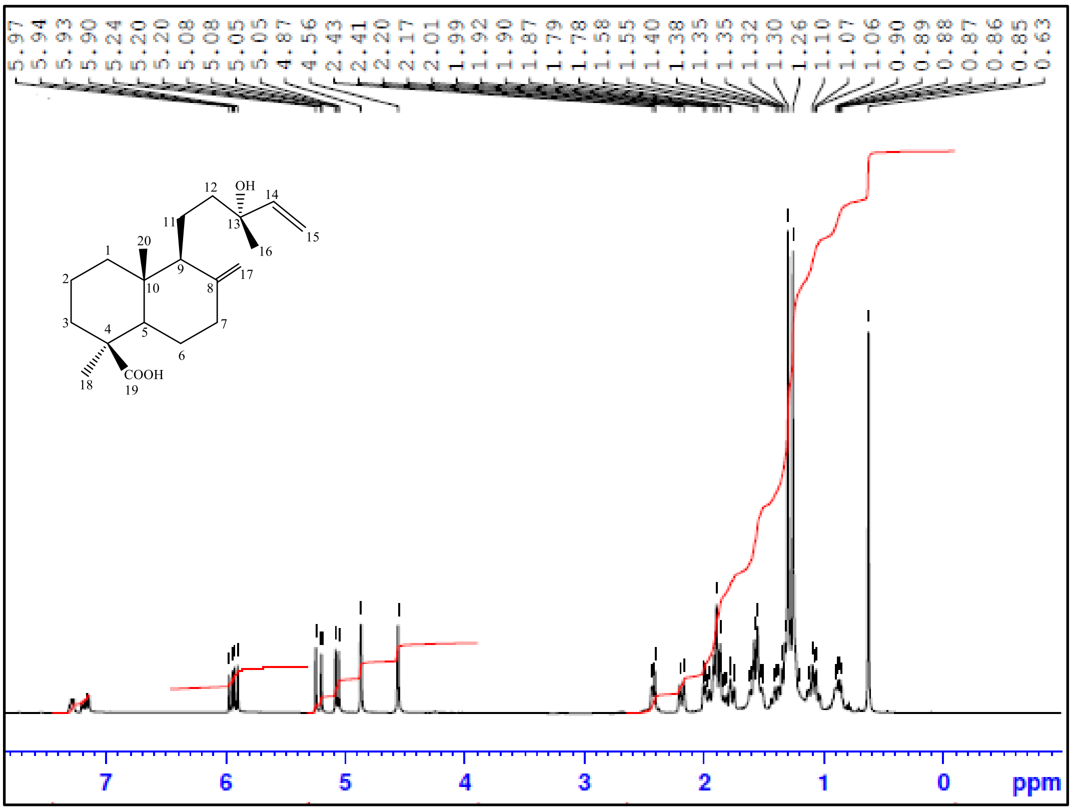

b)

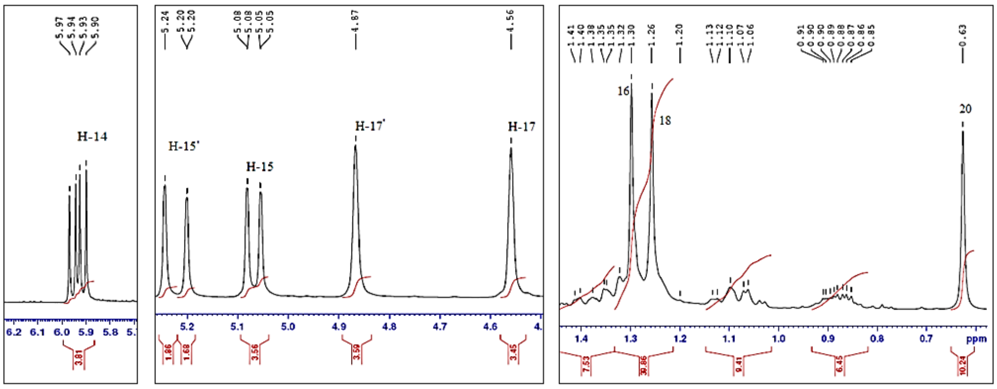

Figure S2. a) Full and b) selected expansions of ^{1}H NMR spectrum compound **1**.

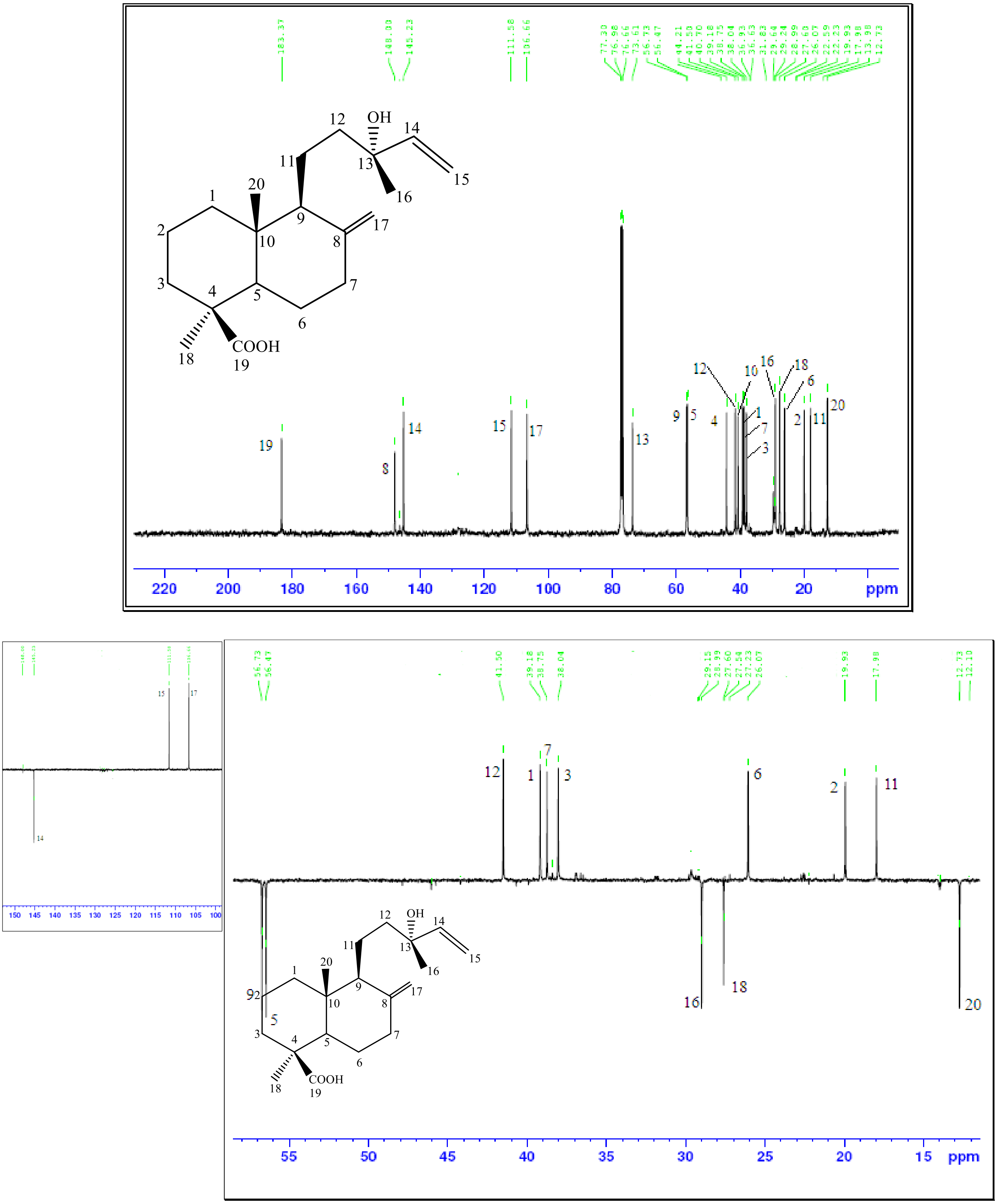

Figure S3. ^{13}C NMR and APT of compound **1**.

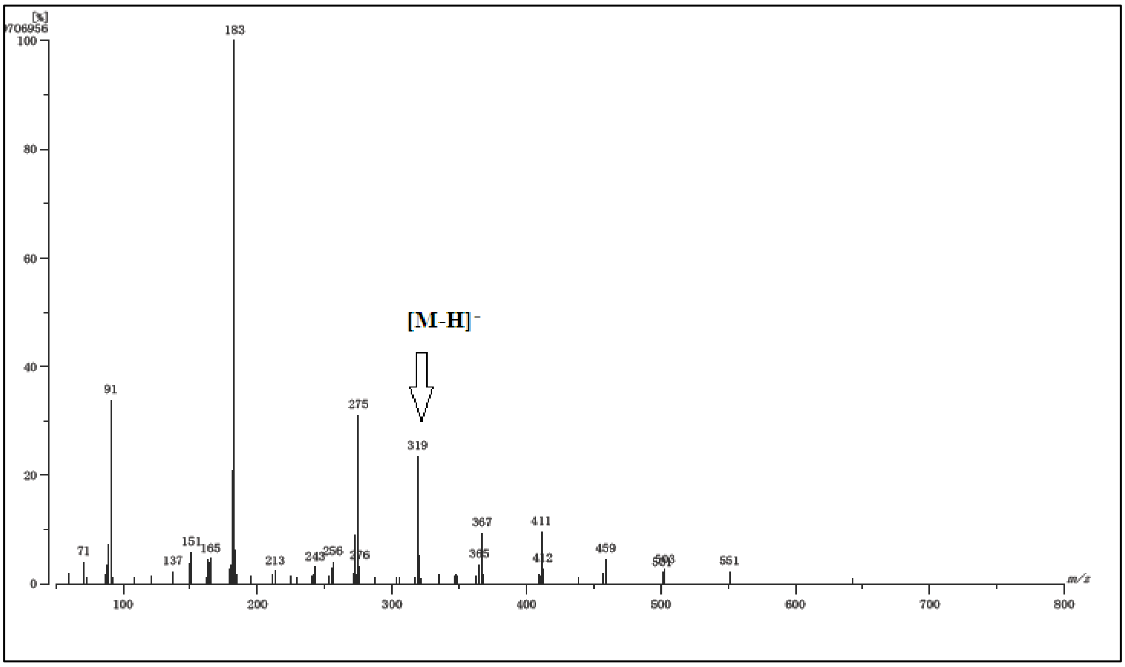

Figure S4. Mass spectroscopy of compound **1**.

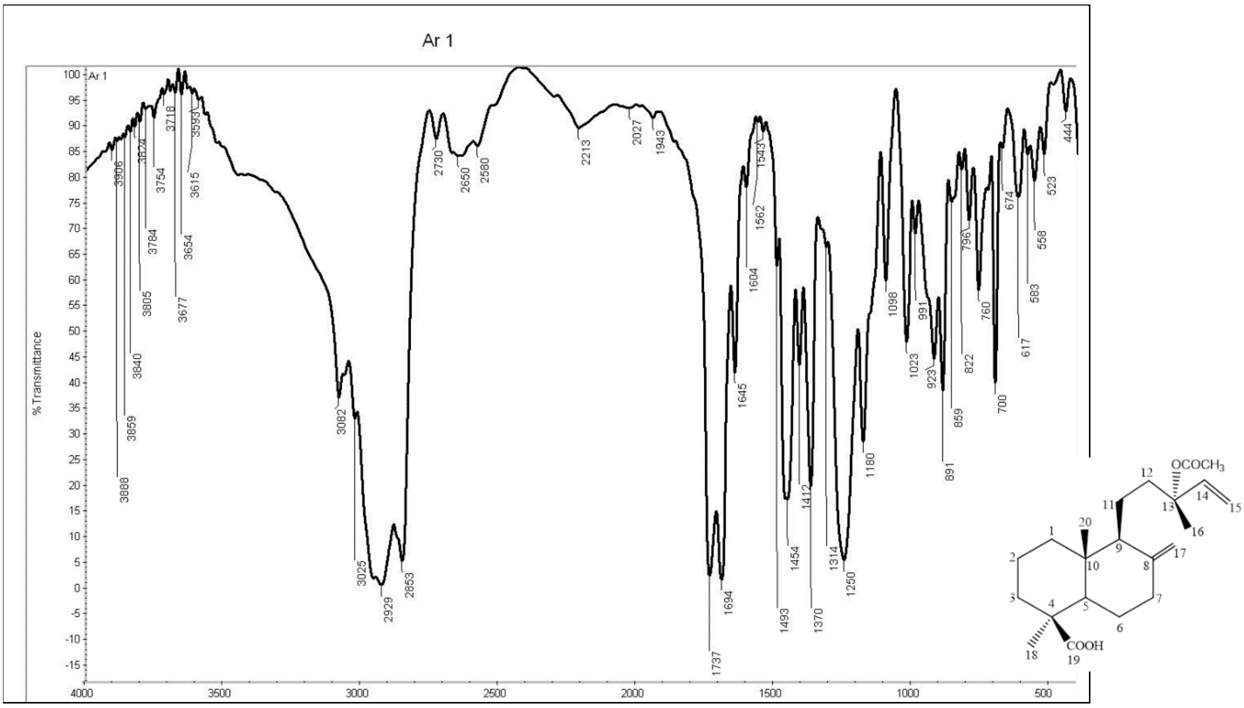

Figure S5. IR spectrum of compound **2**.

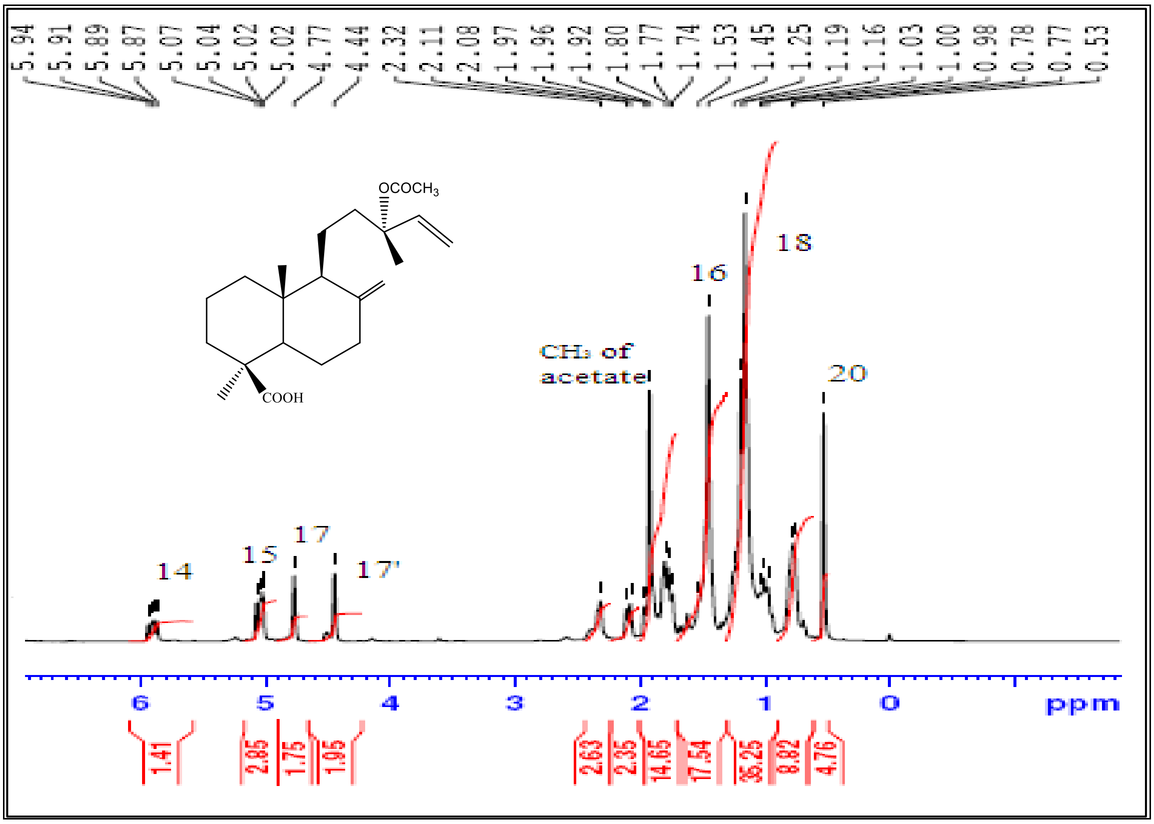

Figure S6. ^{1}H NMR spectrum of compound **2**.

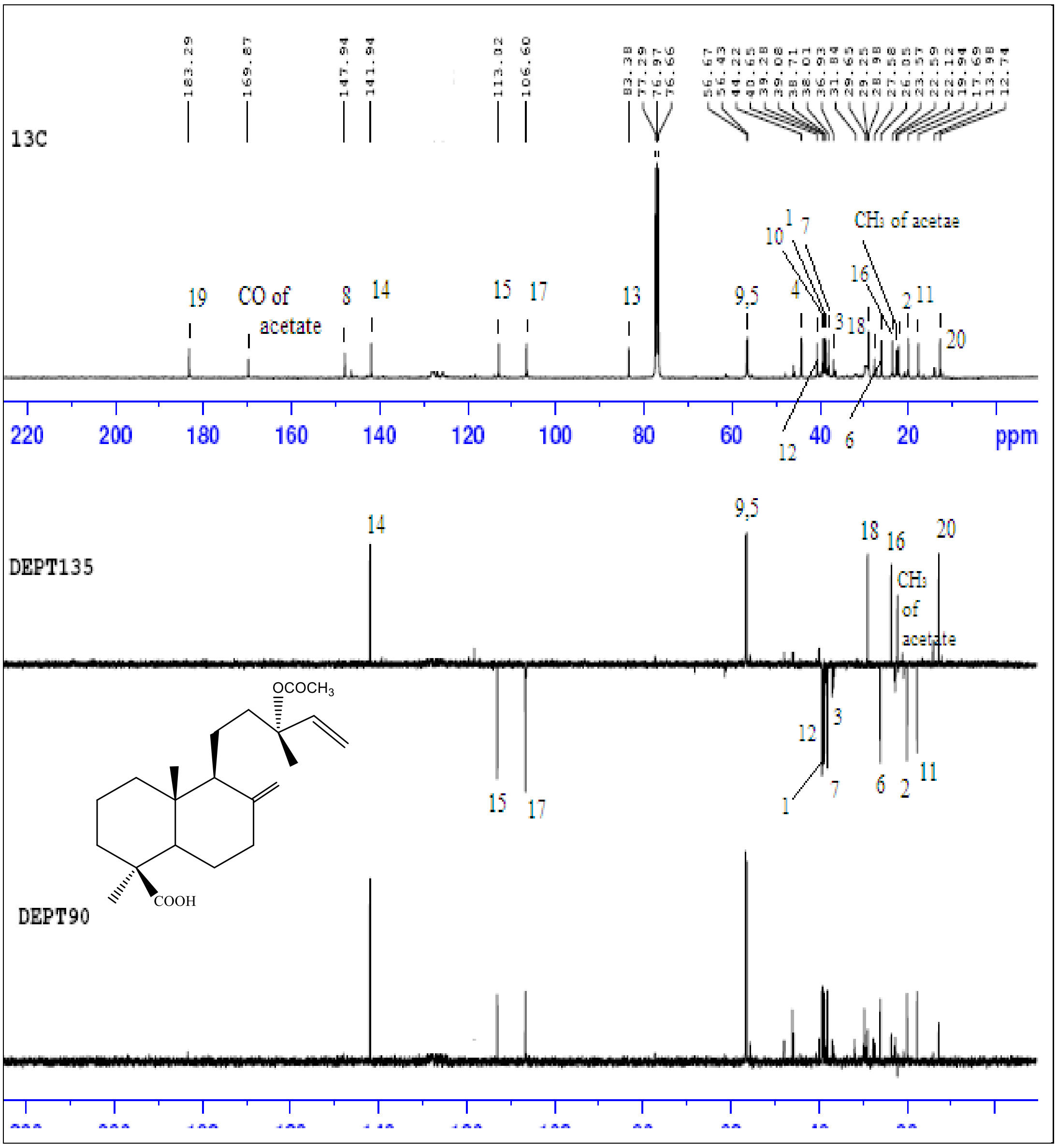

Figure S7. DEPT spectrum of compound **2**.

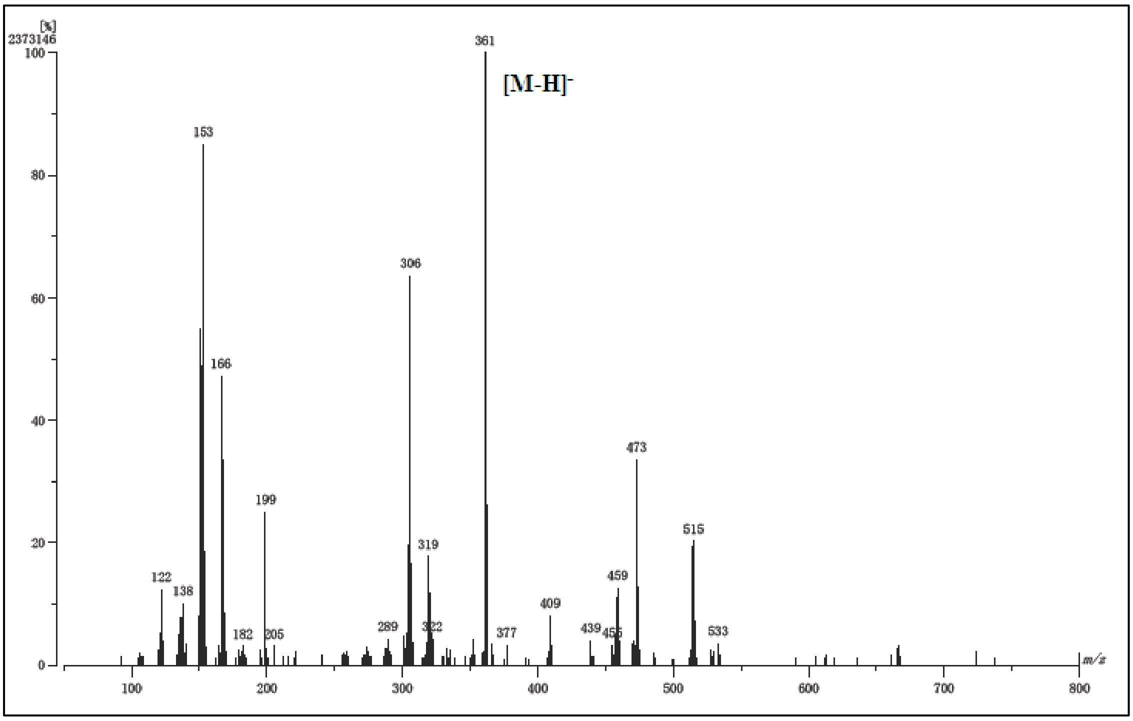

Figure S8. Mass spectroscopy of compound **2**.

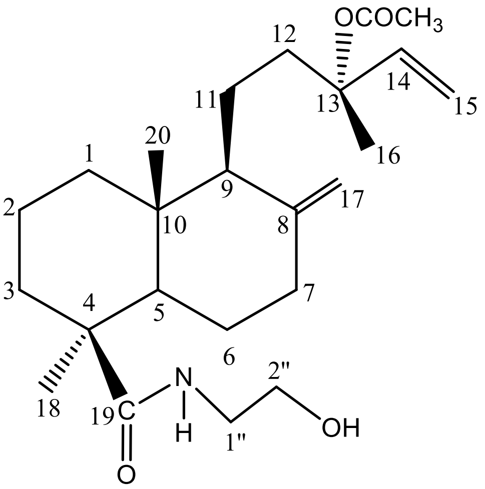

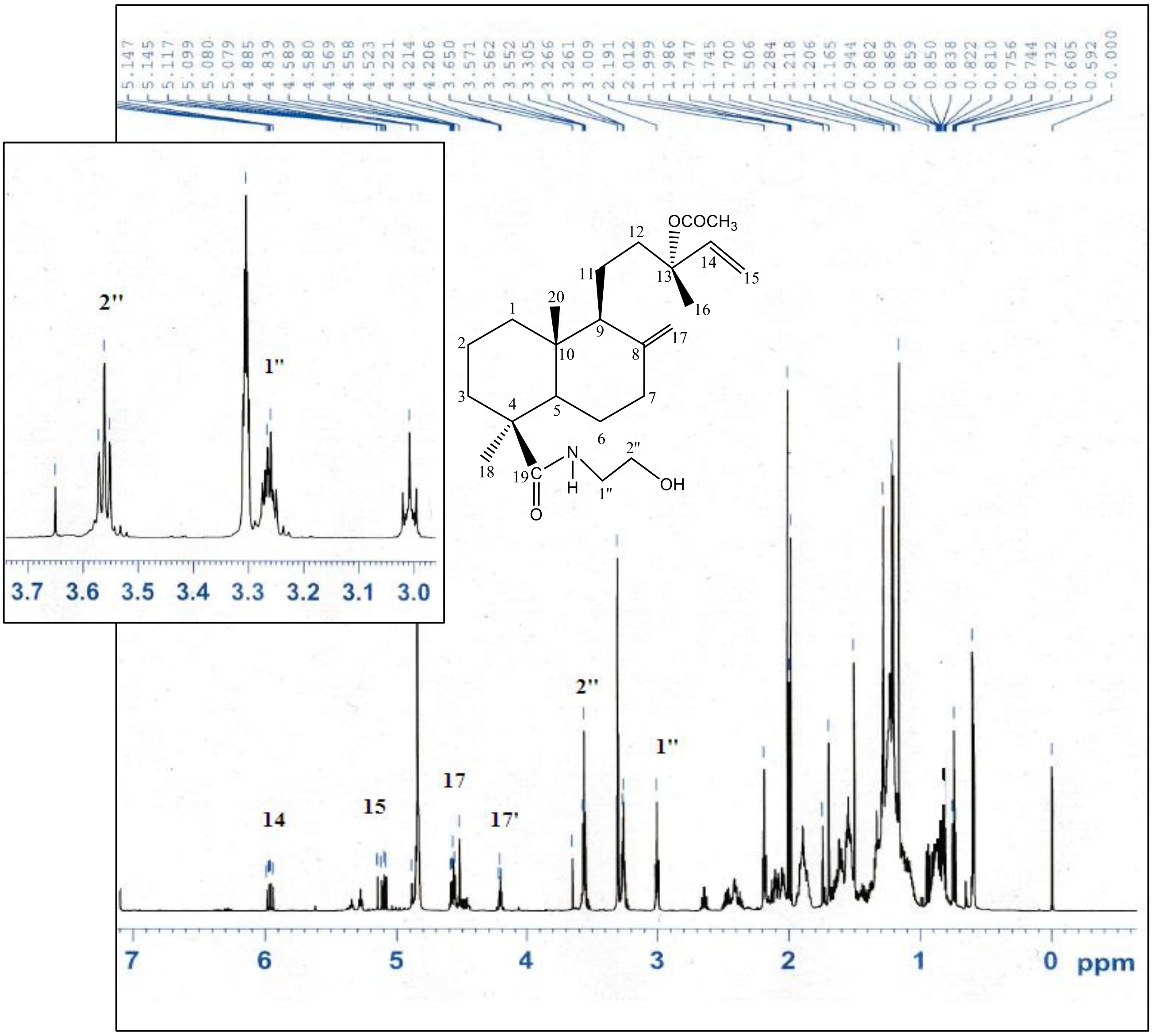

Figure S9. ^{1}H NMR spectrum of compound **3**.

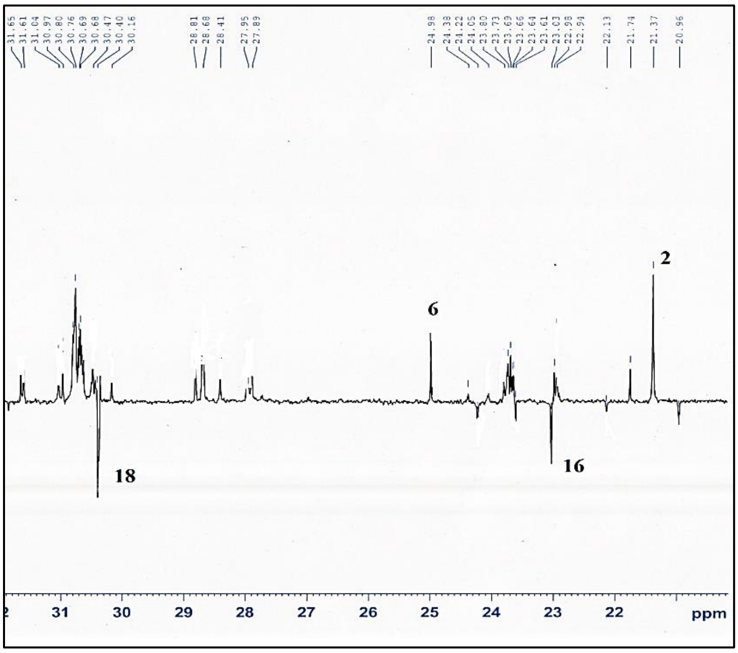

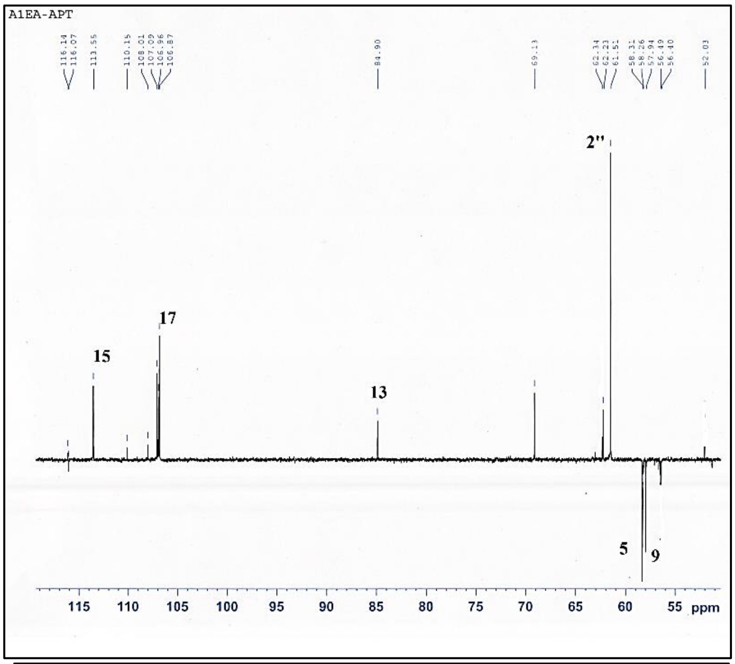

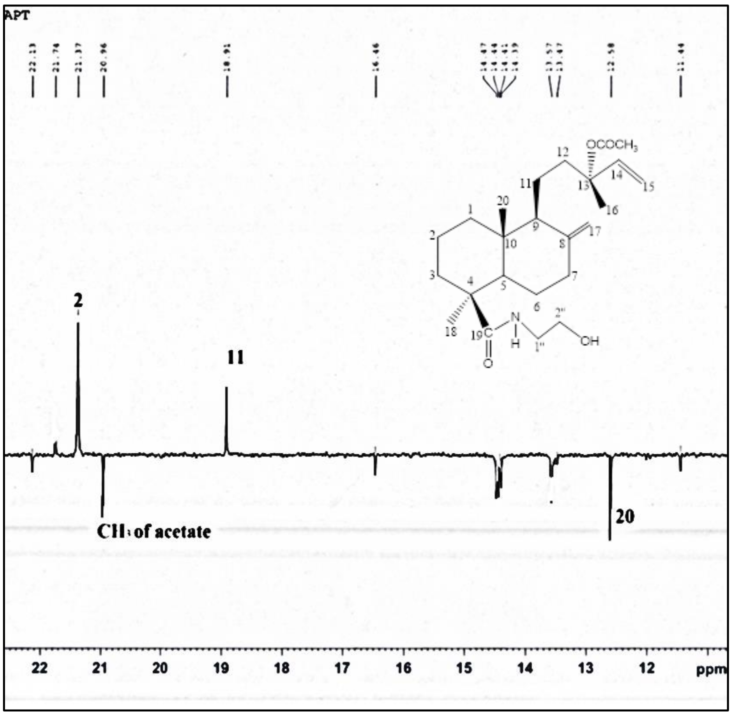

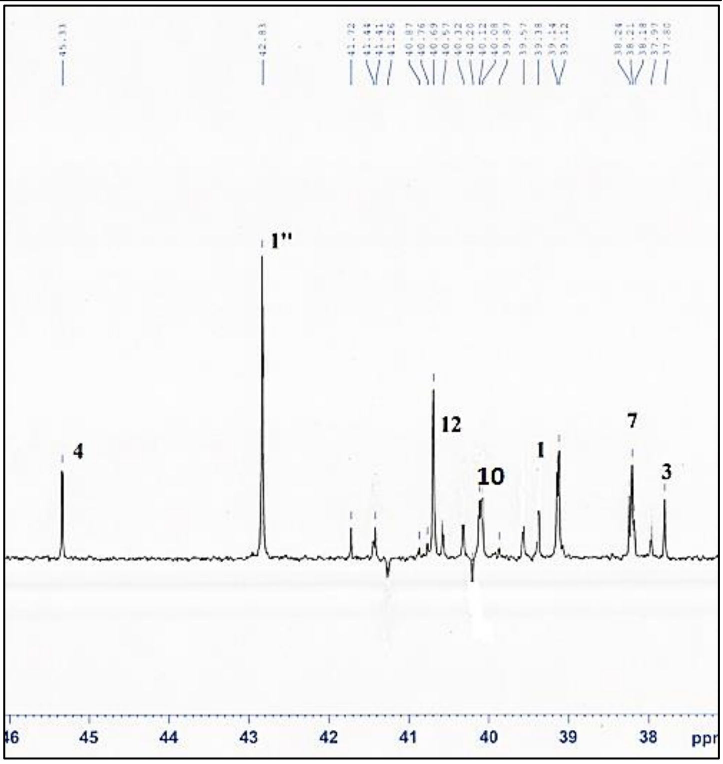

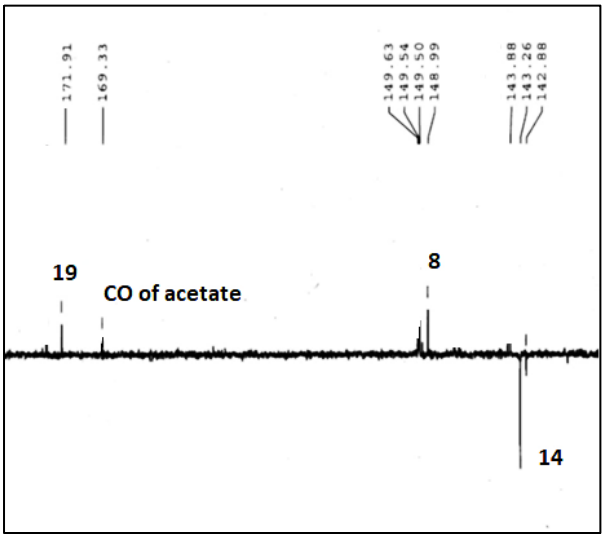

Figure S10. APT spectrum of compound **3**.

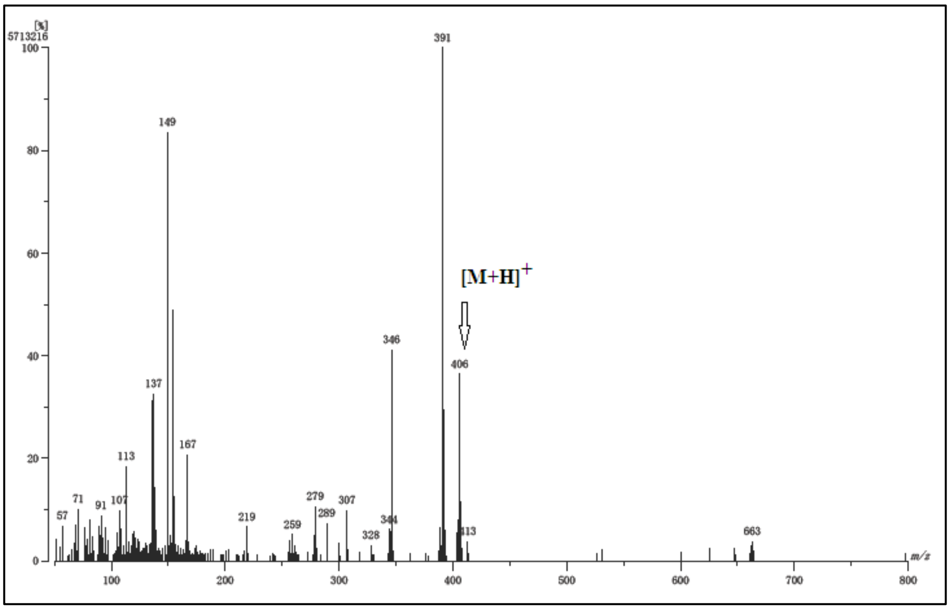

Figure S11. Mass spectroscopy of compound **3**.

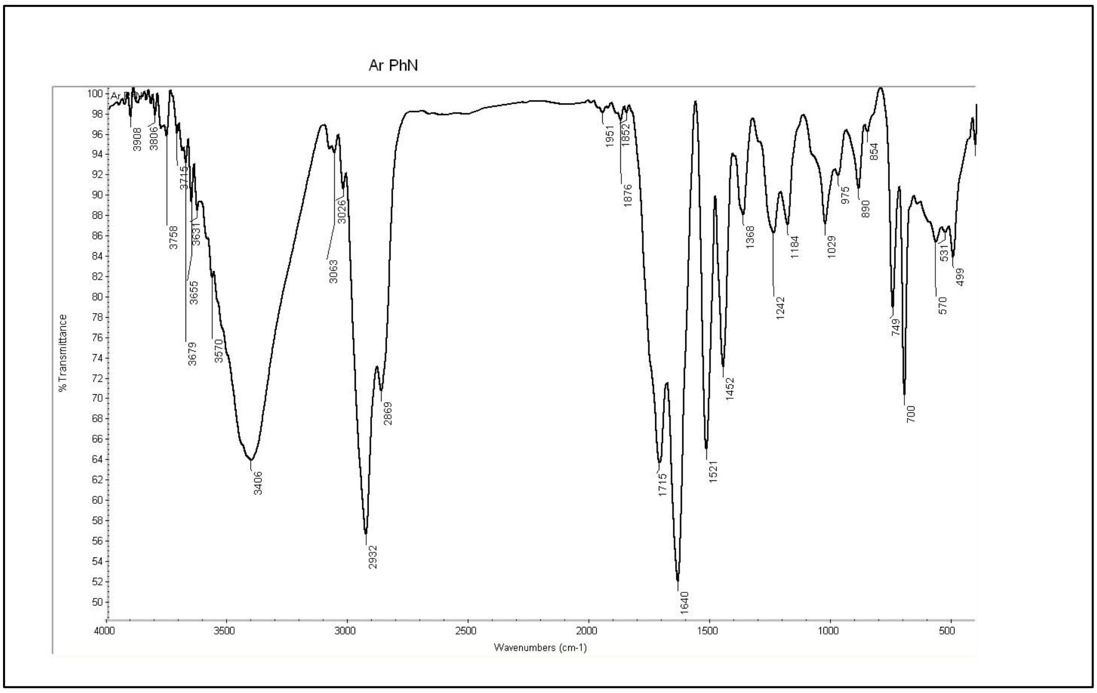

Figure S12. IR spectrum of compound **4**.

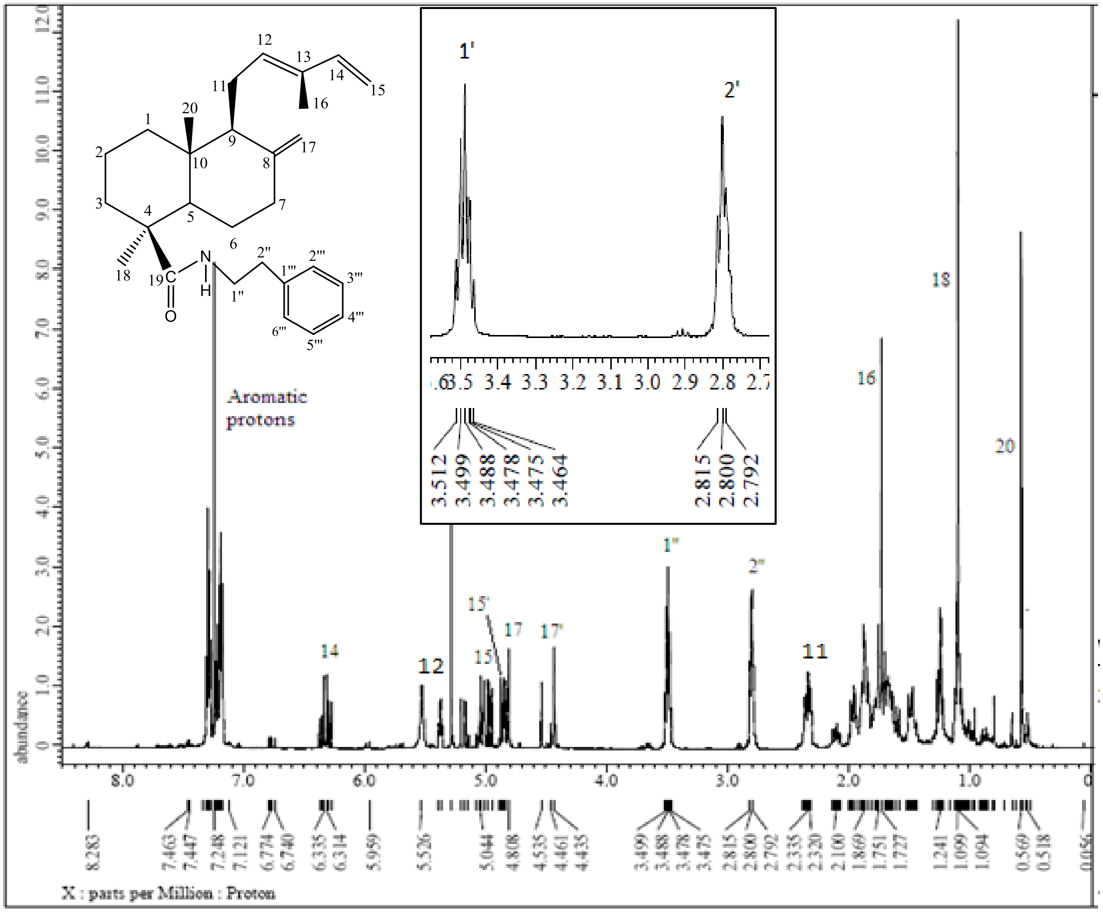

Figure S13. ^{1}H NMR spectrum of compound **4**.

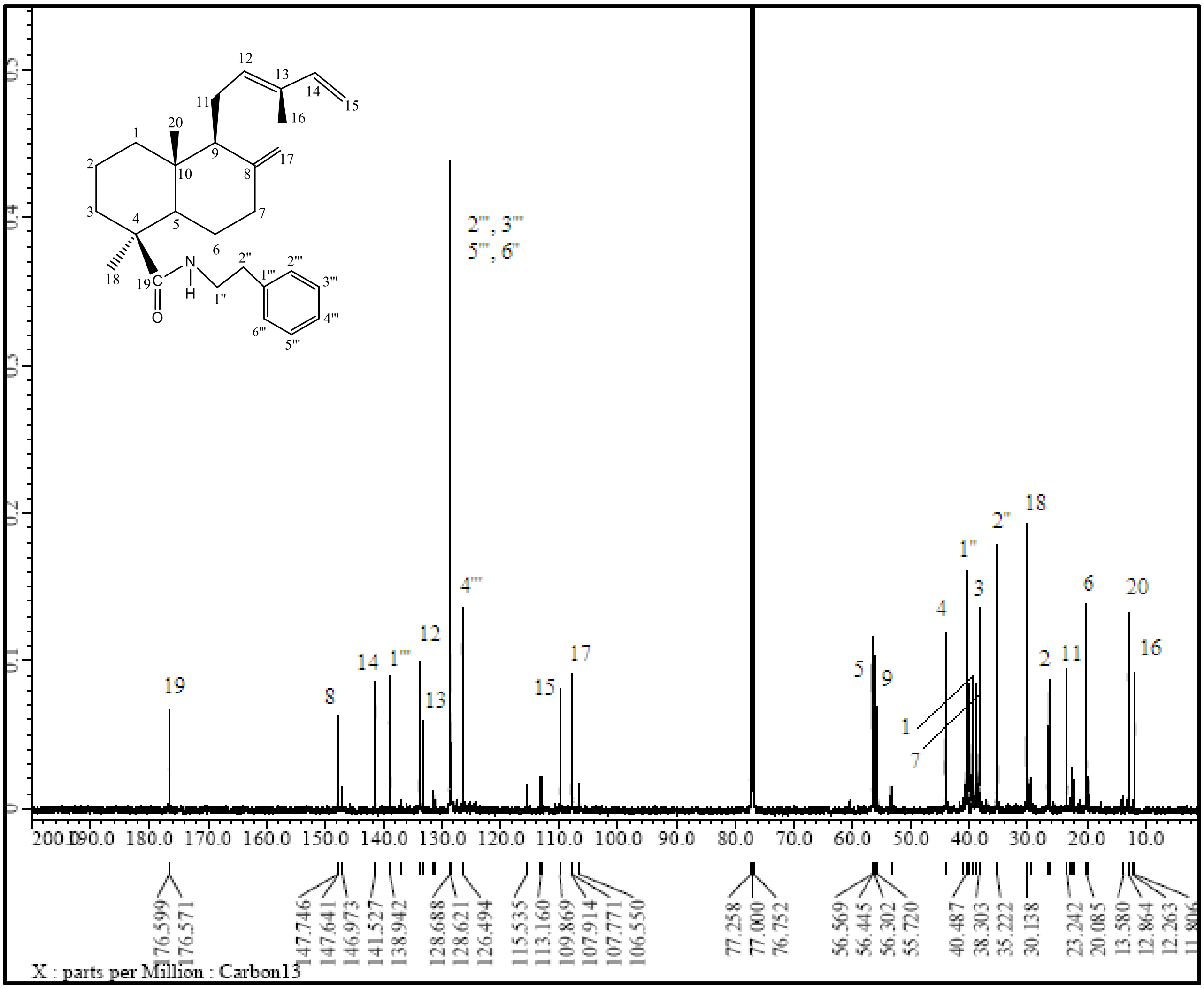

Figure S14. ^{13}C NMR of compound **4**.

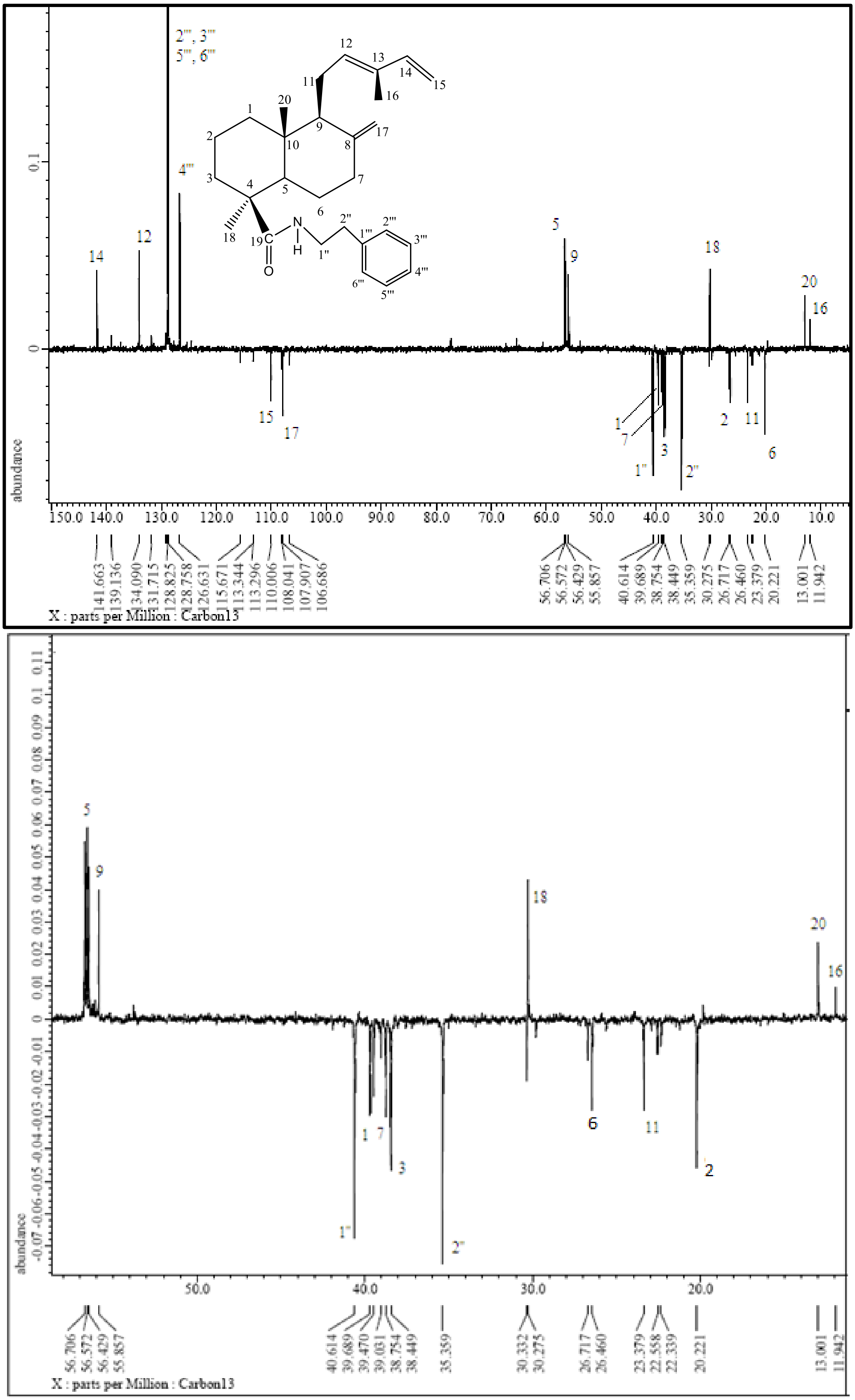

Figure S15. APT spectrum of compound **4**.

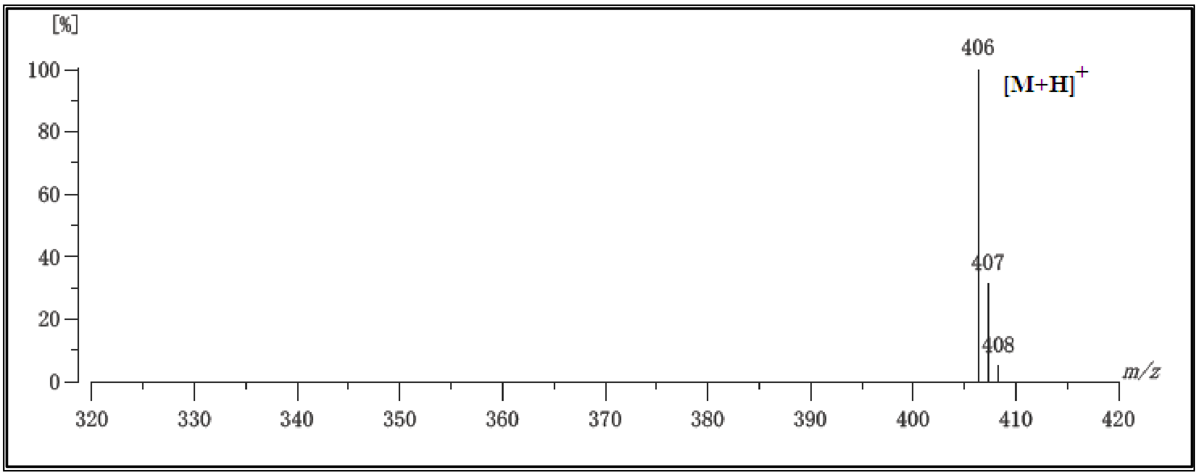

Figure S16. Mass spectroscopy of compound **4**.

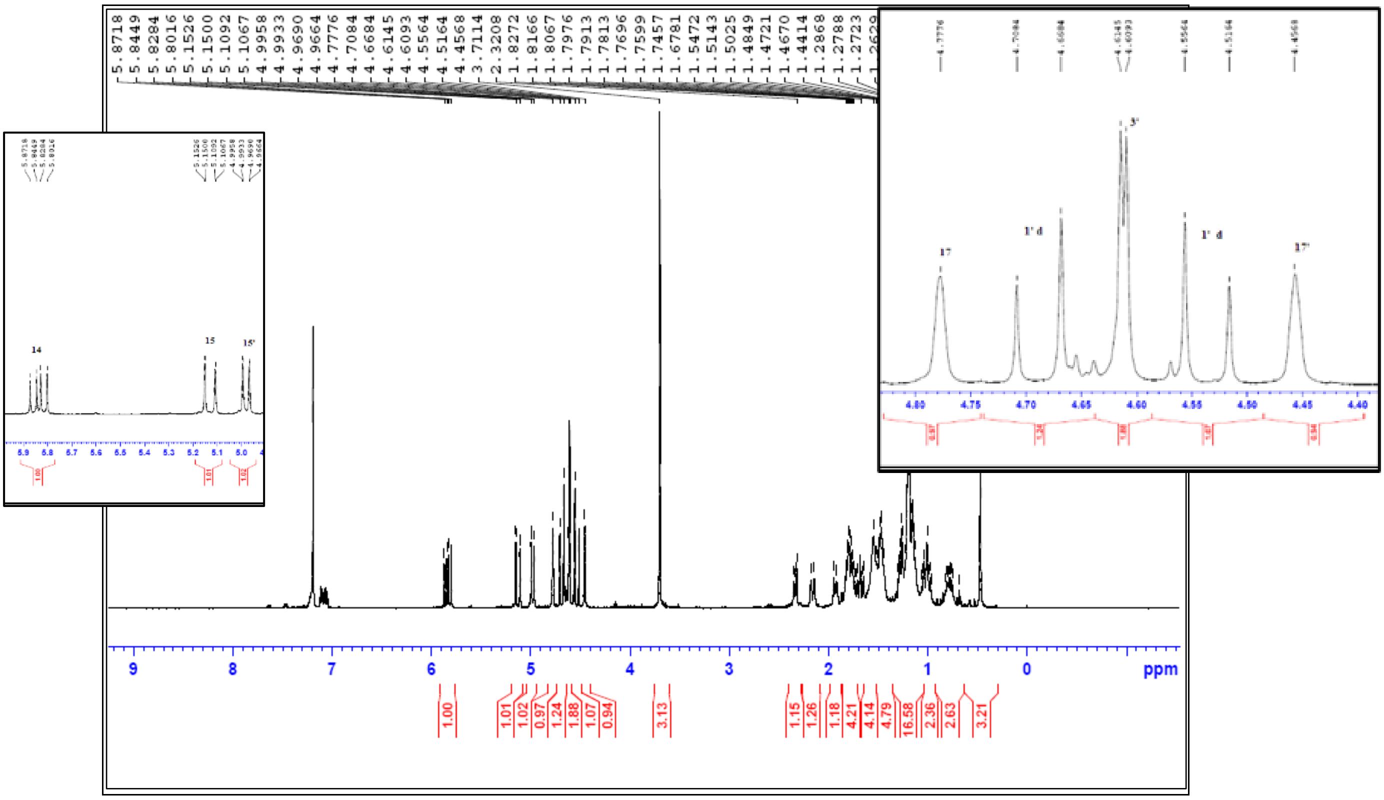

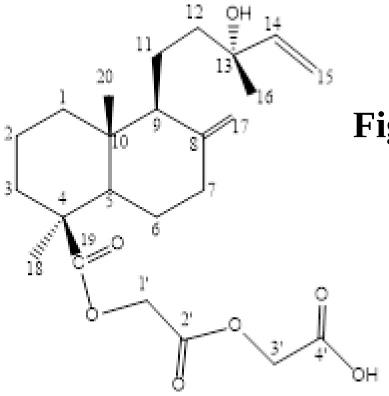

Figure S17. ^{1}H NMR spectrum of compound **5**.

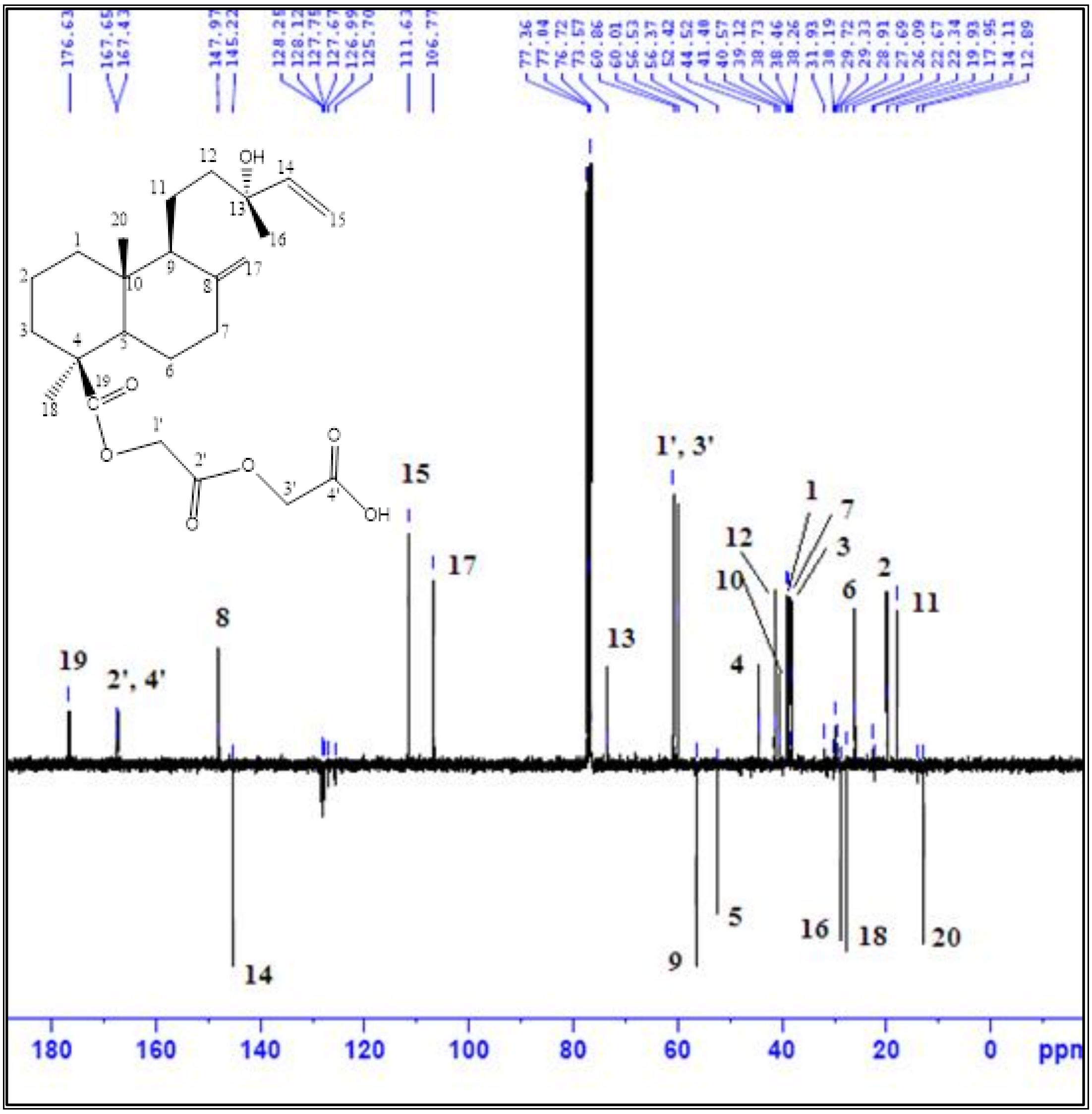

Figure S18. APT spectrum of compound **5**.

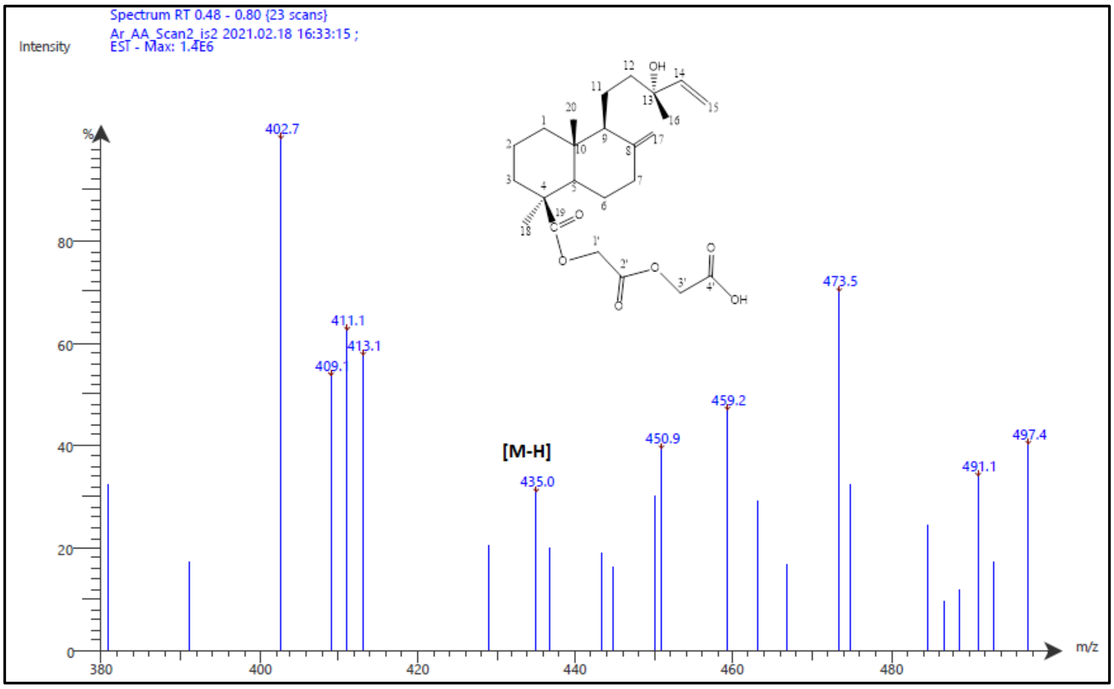

Figure S19. Mass spectroscopy of compound **5**.

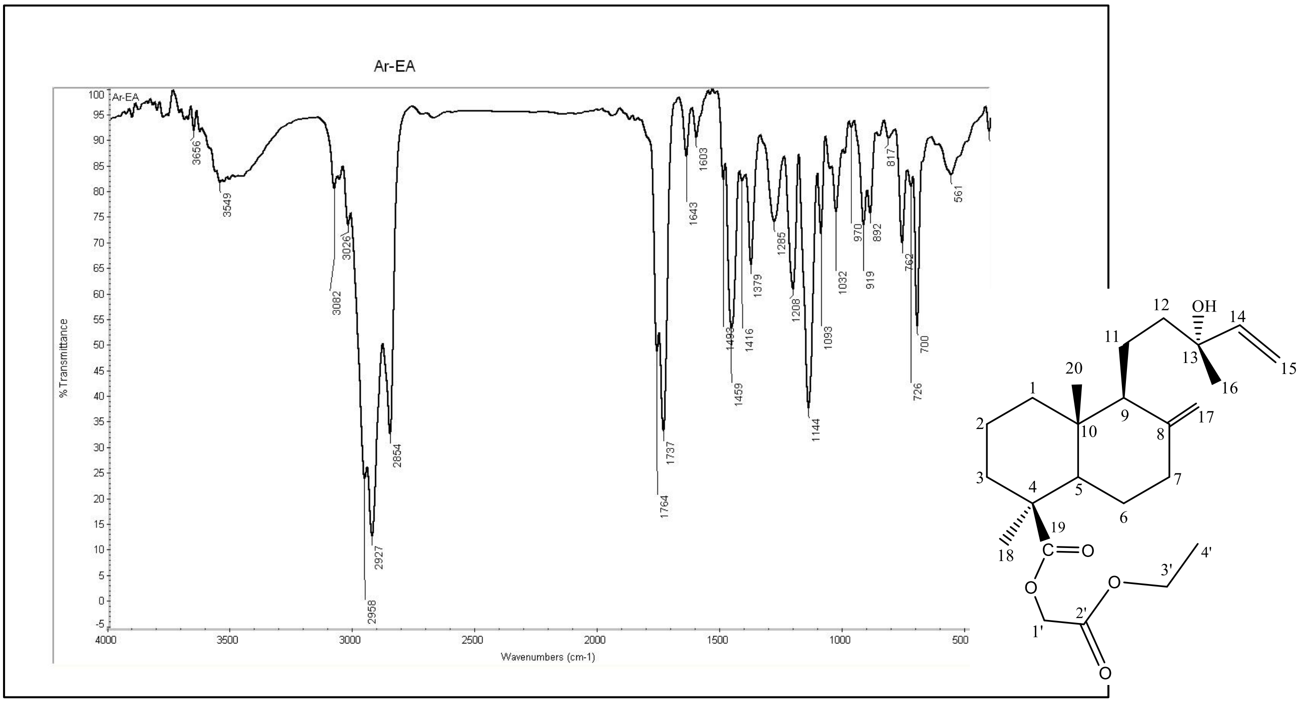

Figure S20. IR spectrum of compound **6**.

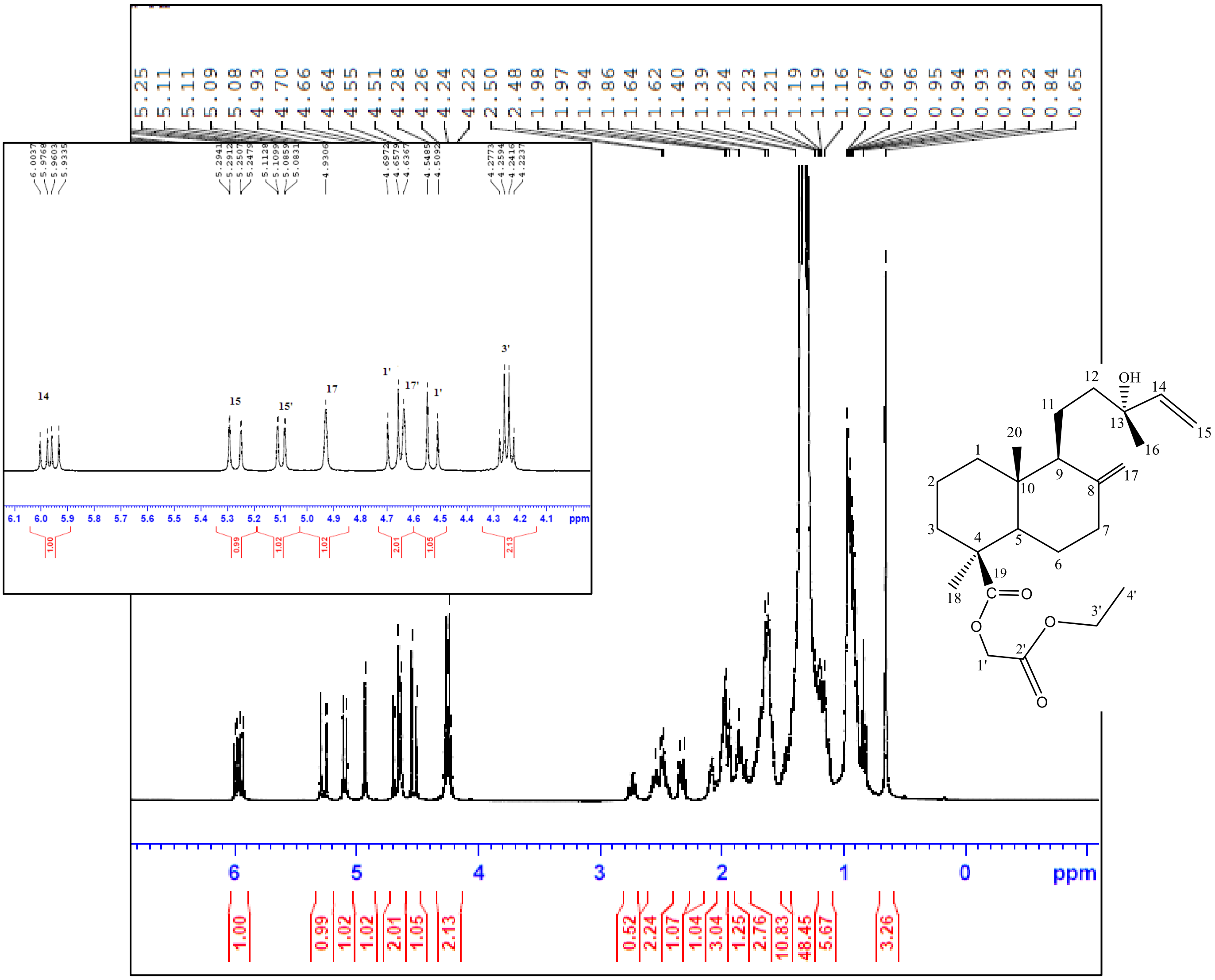

Figure S21. ^{1}H NMR spectrum of compound **6**.

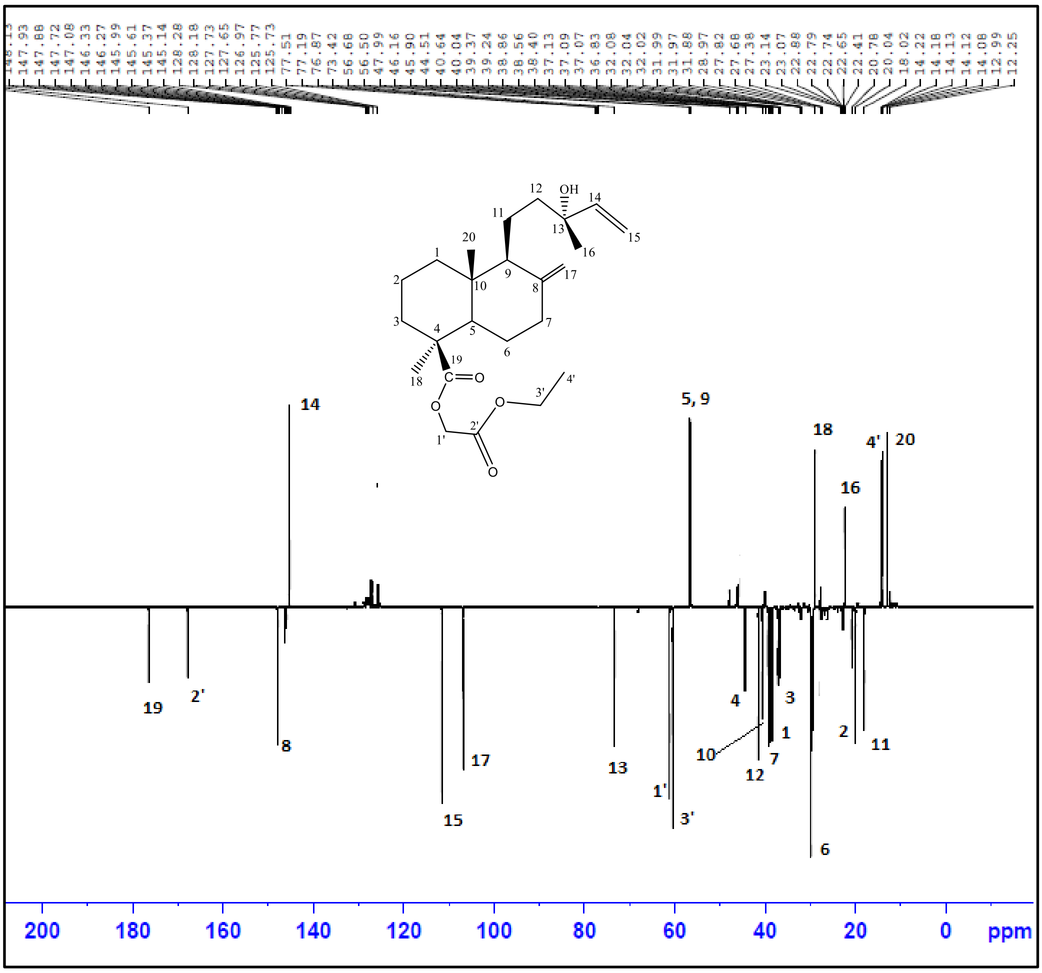

Figure S22. APT spectrum of compound **6**.

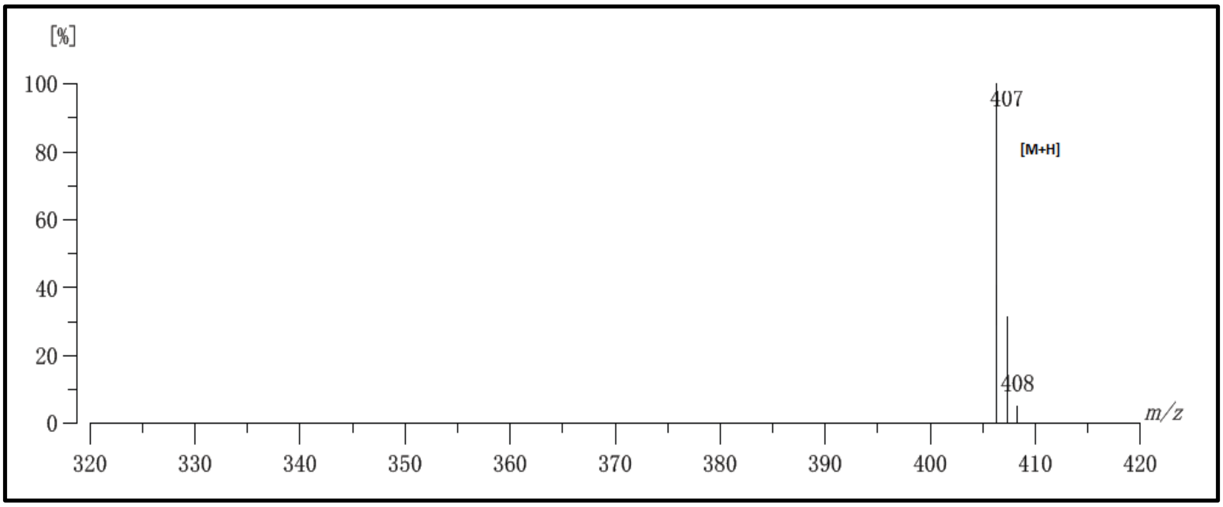

Figure S23. Mass spectroscopy of compound **6**.

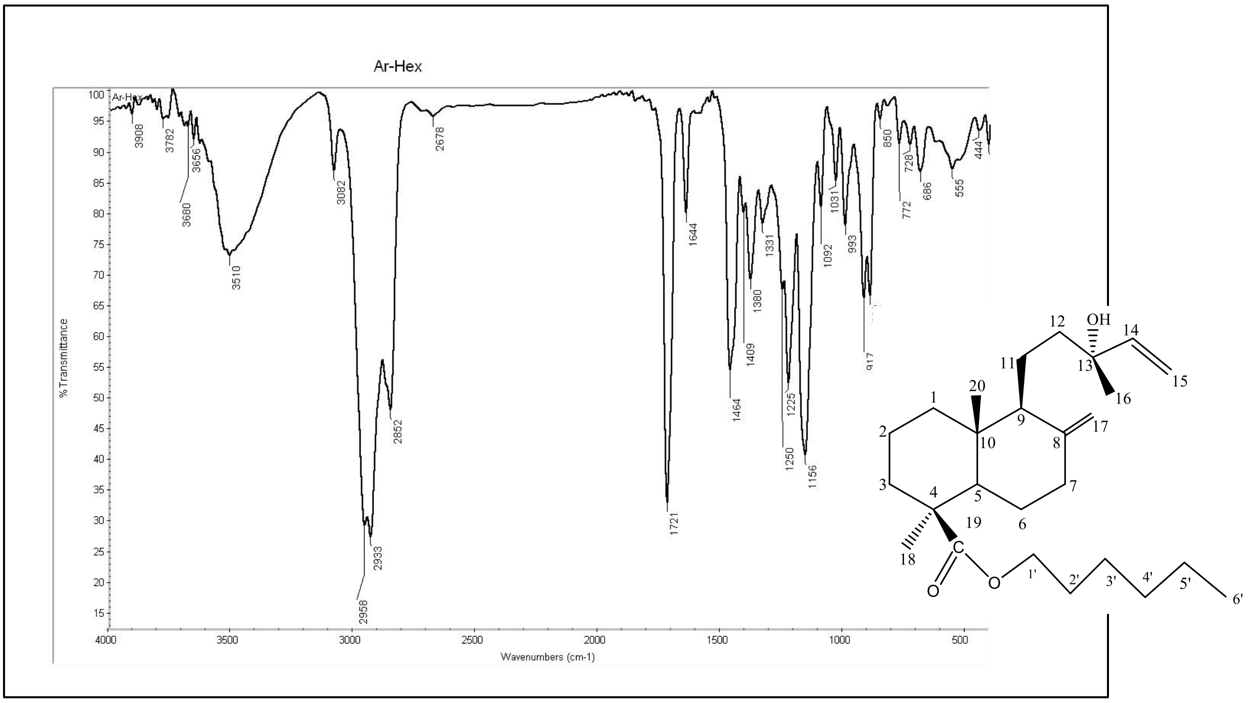

Figure S24. IR spectrum of compound **7**.

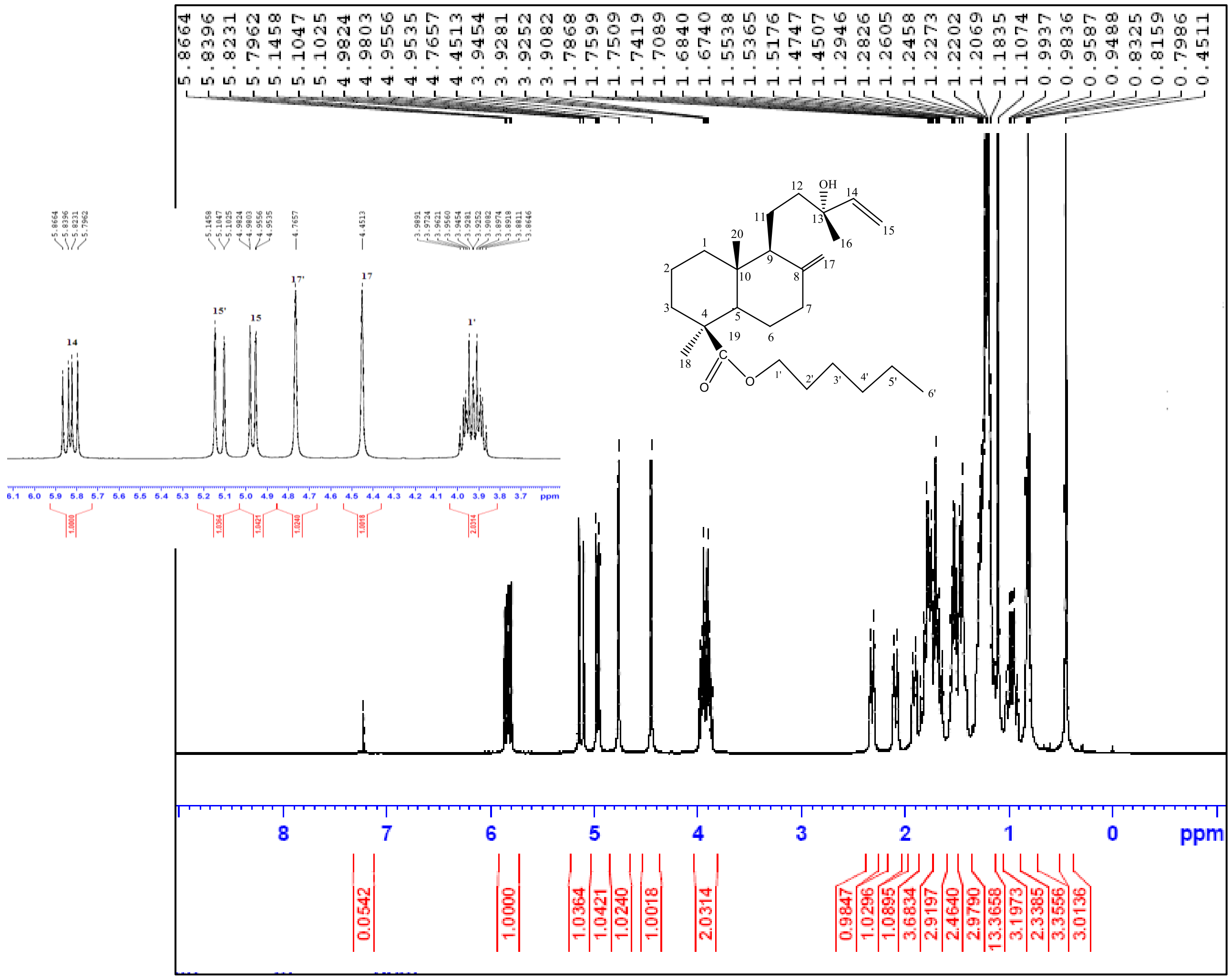

Figure S25. ^{1}H NMR spectrum of compound **7**.

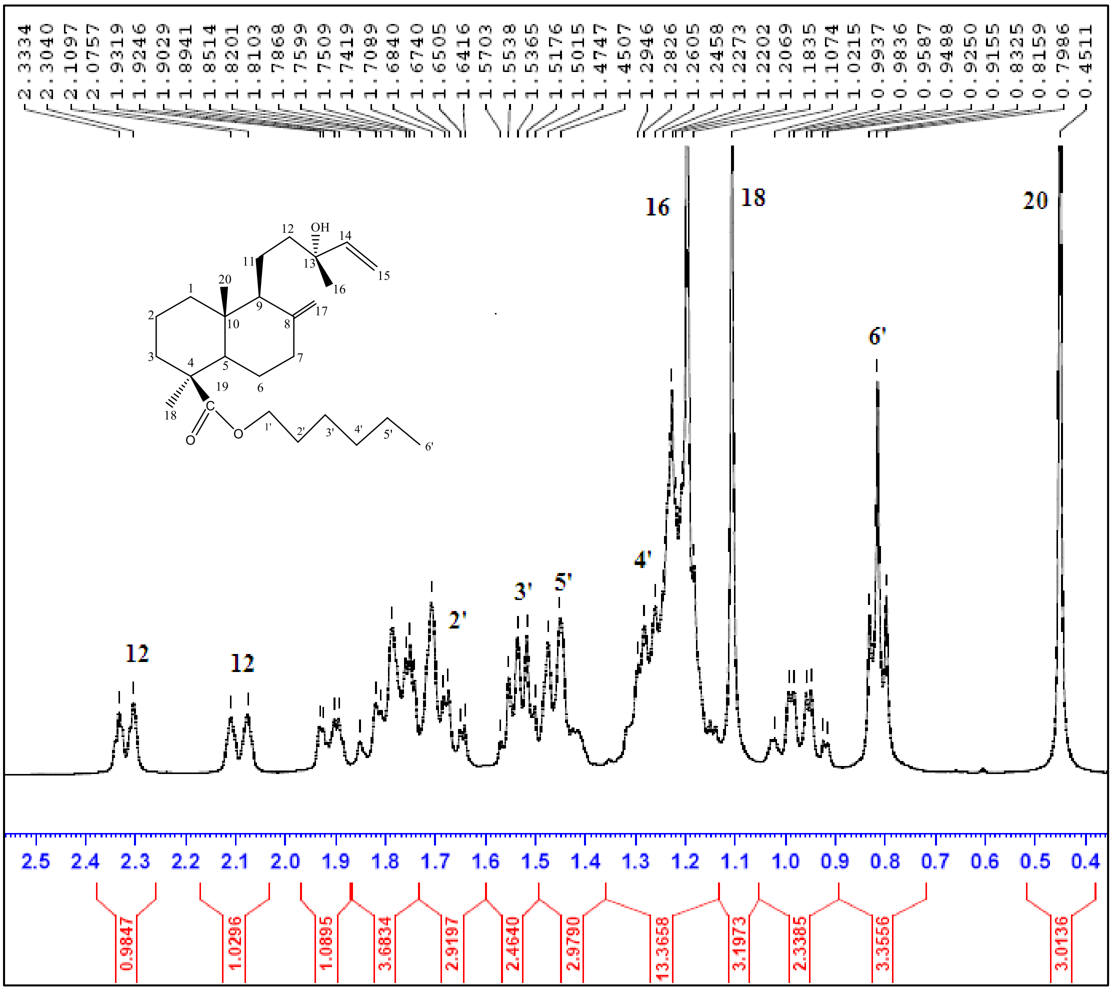

Figure S26. ^{1}H NMR expansion of compound **7**.

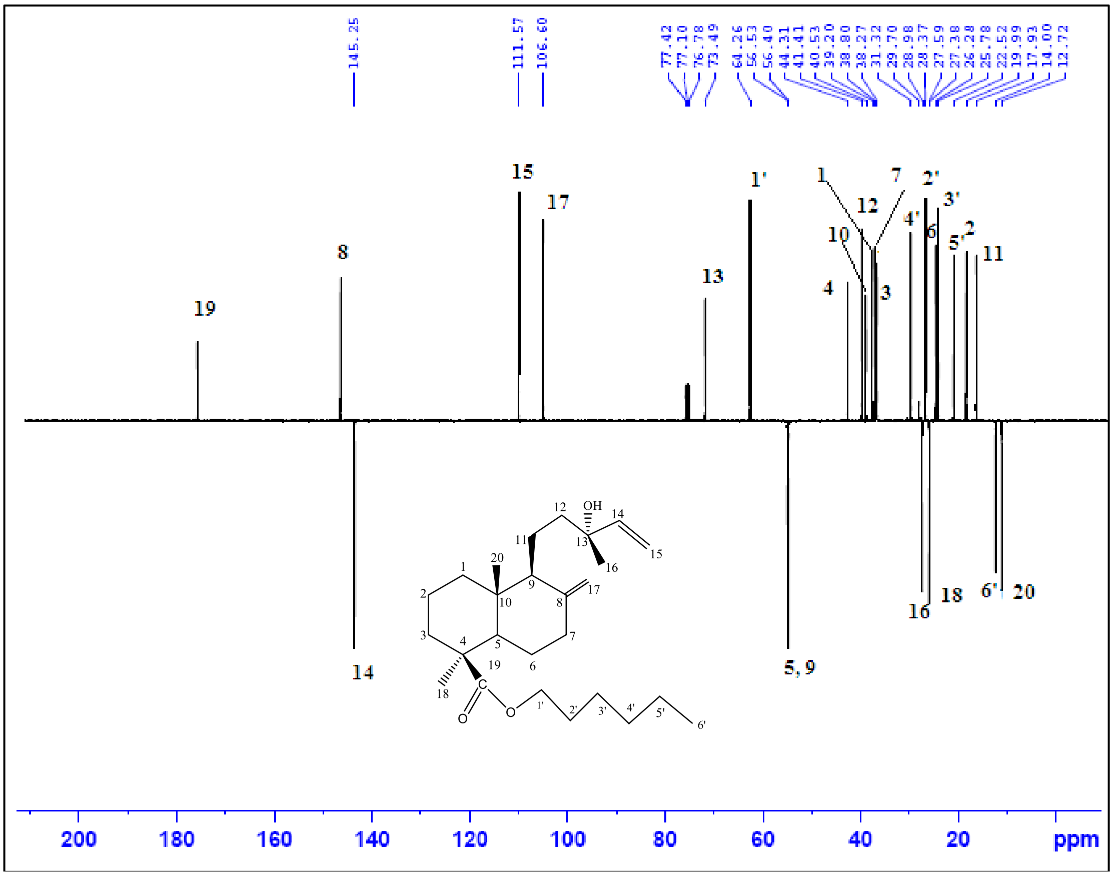

Figure S27. APT spectrum of compound **7**.

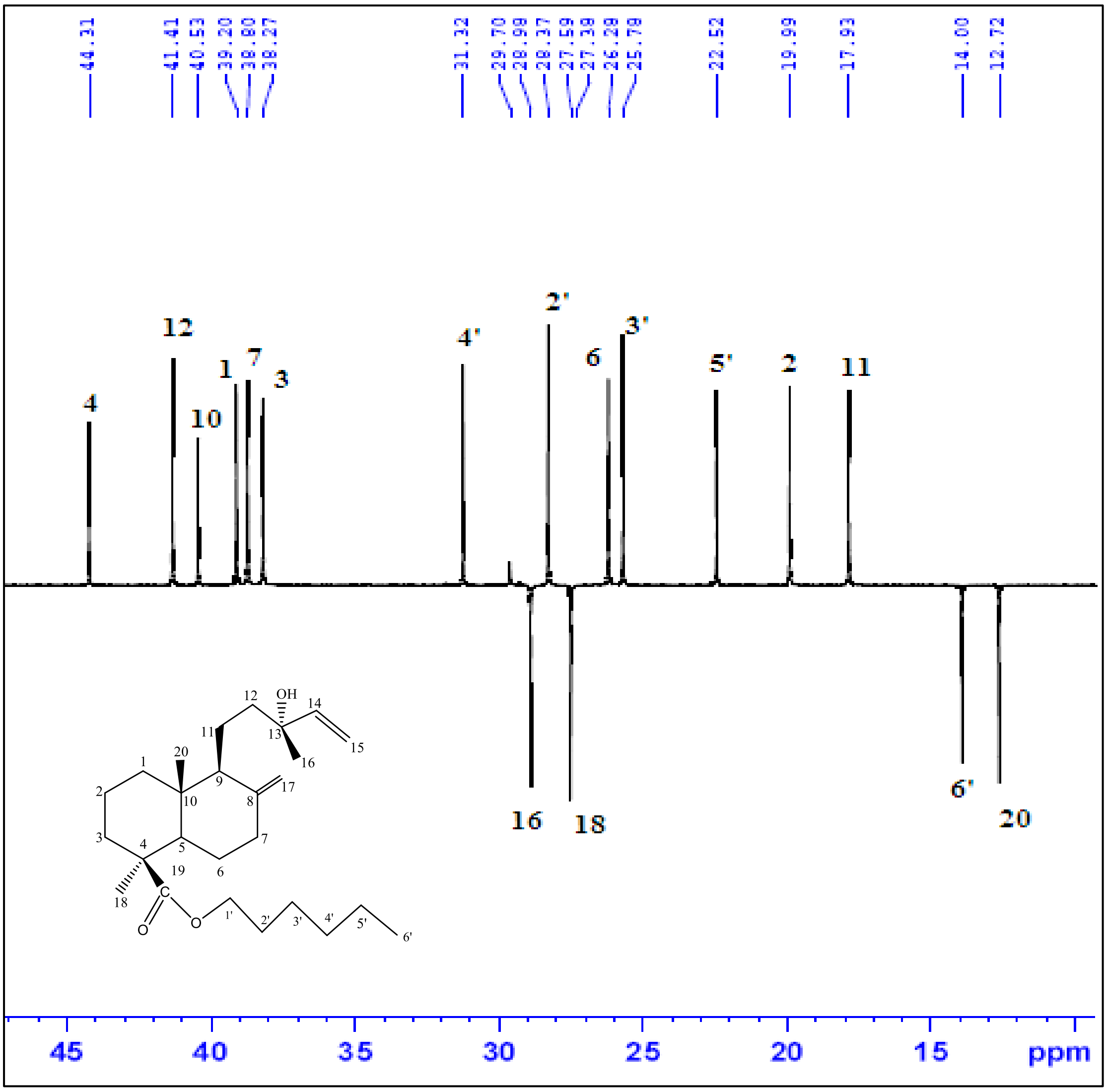

Figure S28. APT spectrum expansion of compound **7**.

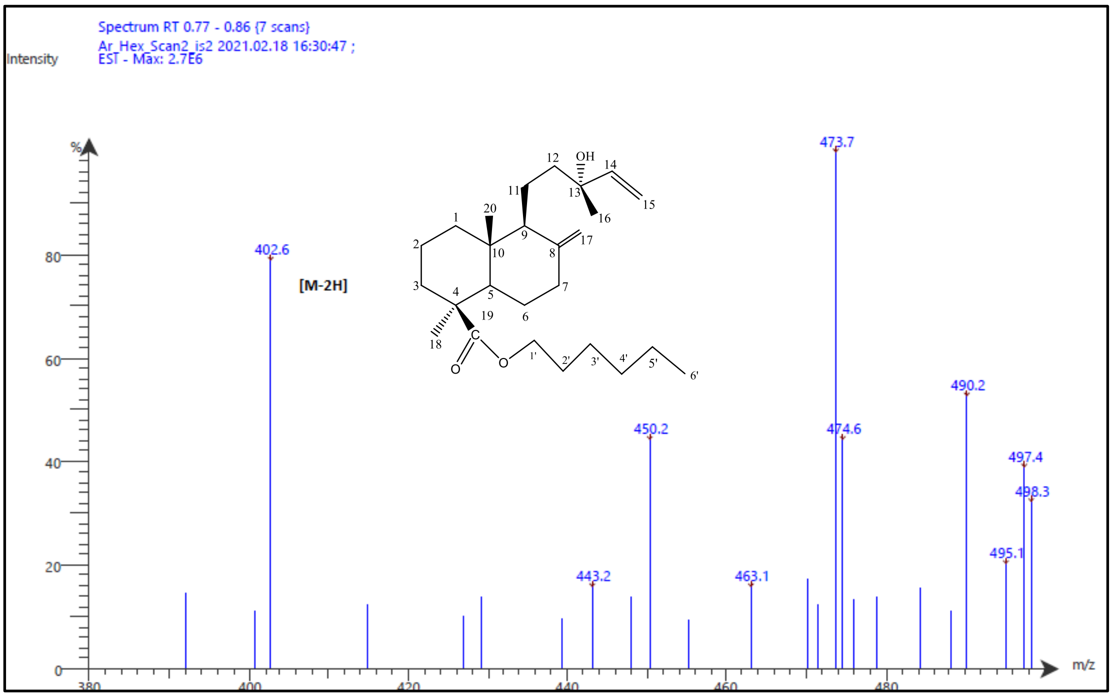

Figure S29. Mass spectroscopy of compound **7**.

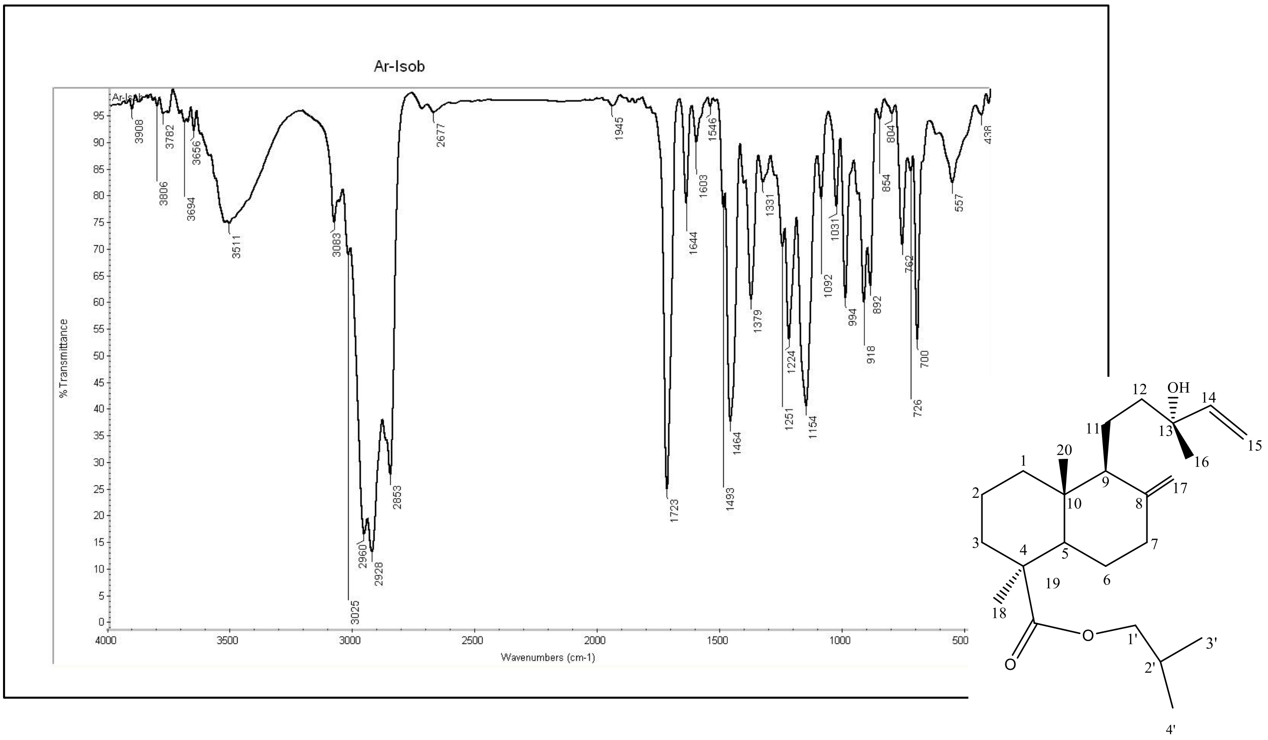

Figure S30. IR spectrum of compound **8**.

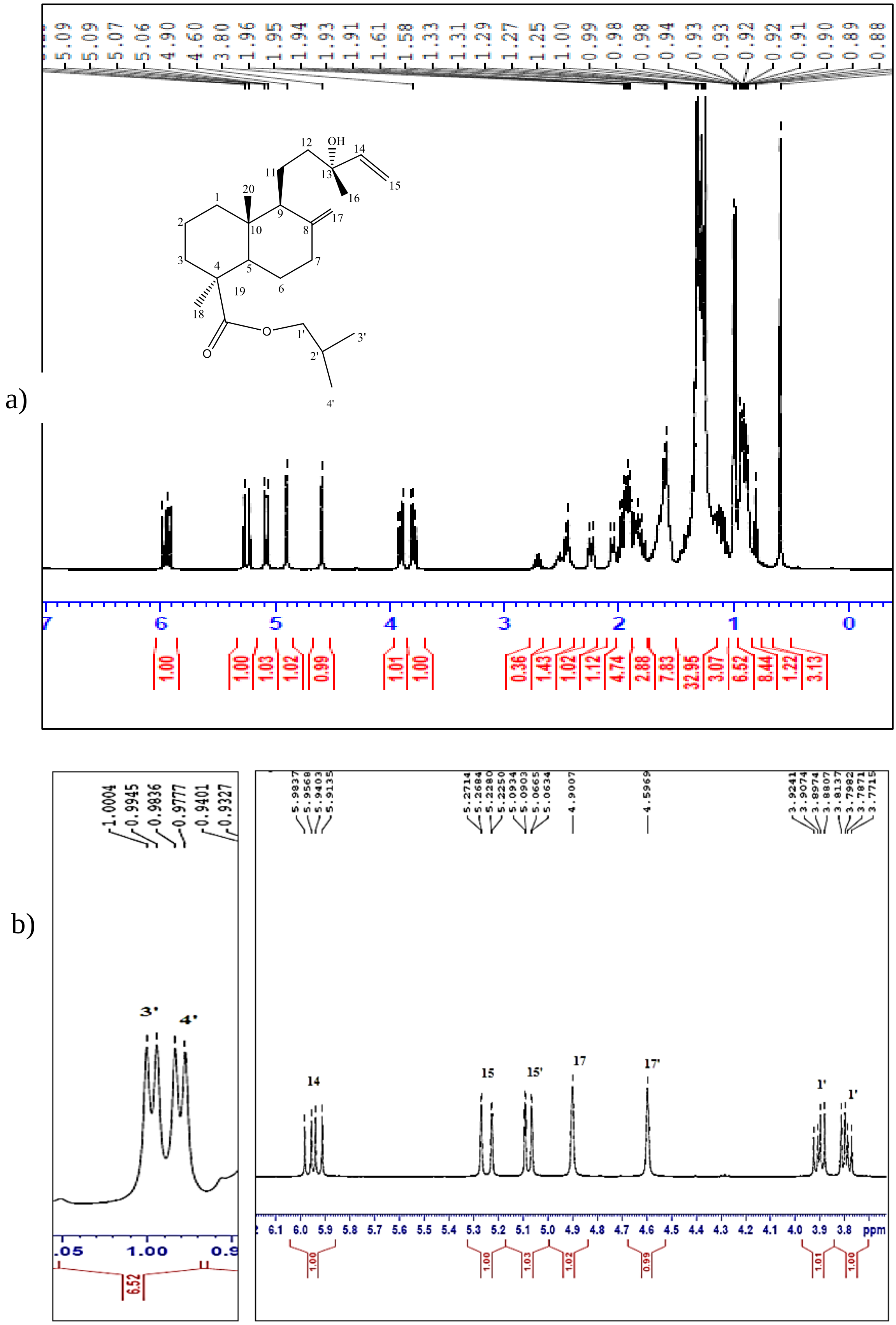

Figure S31. a) Full and b) selected expansions of ^{1}H NMR spectrum of compound **8**.

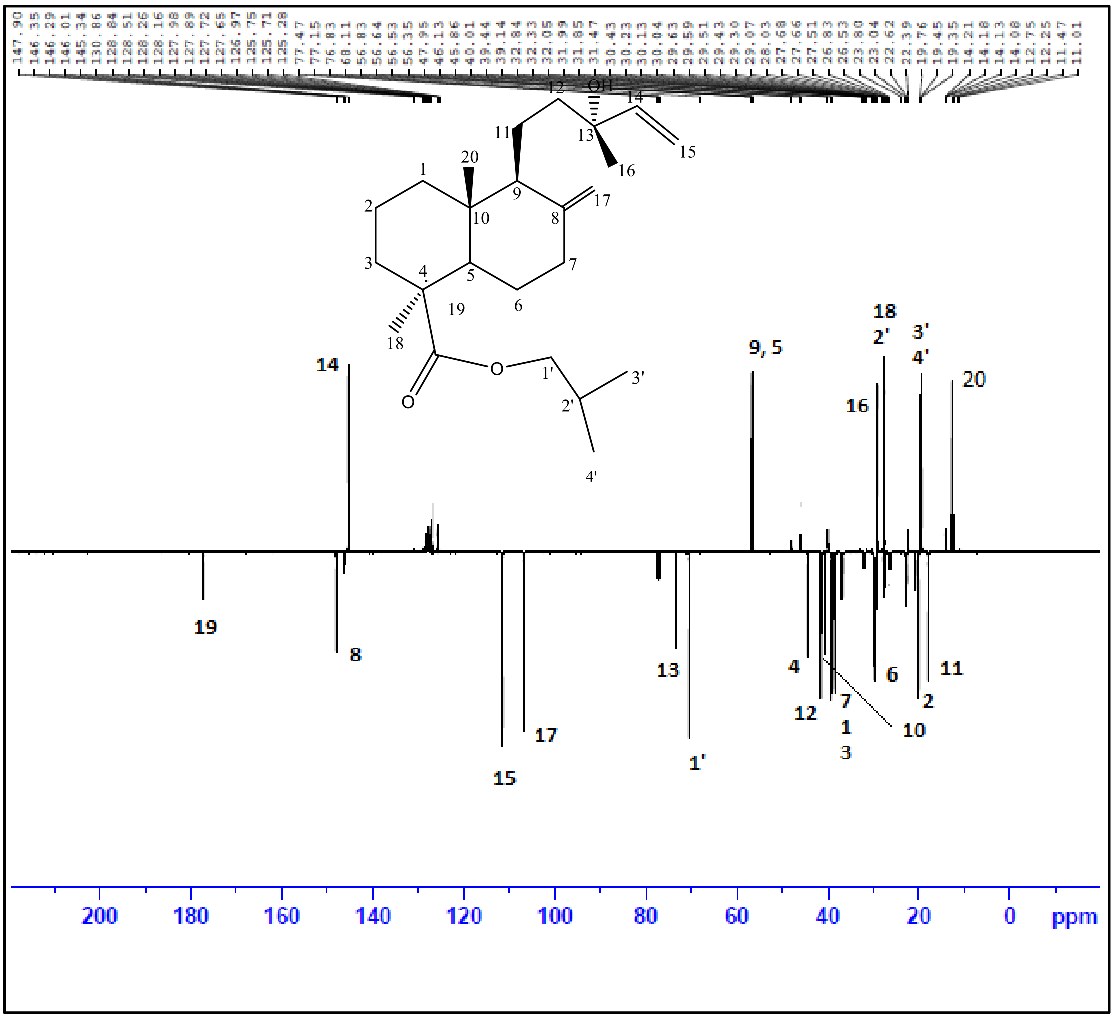

Figure S32. APT spectrum of compound **8**.

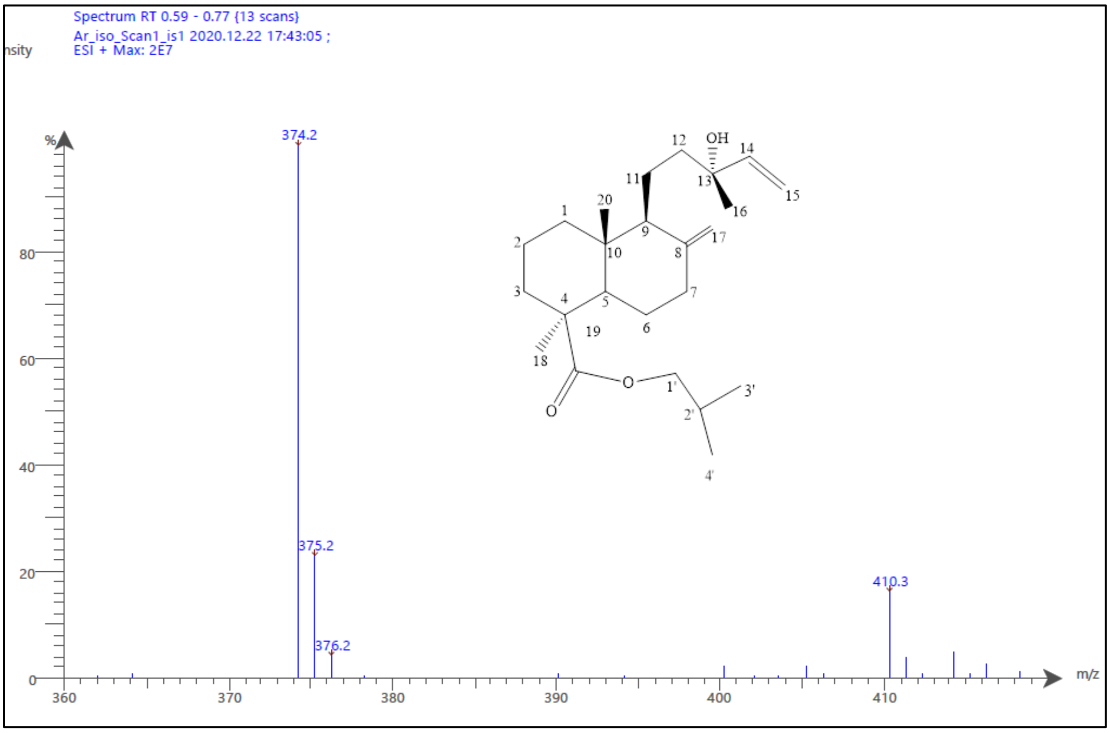

Figure S33. Mass spectroscopy of compound **8**.

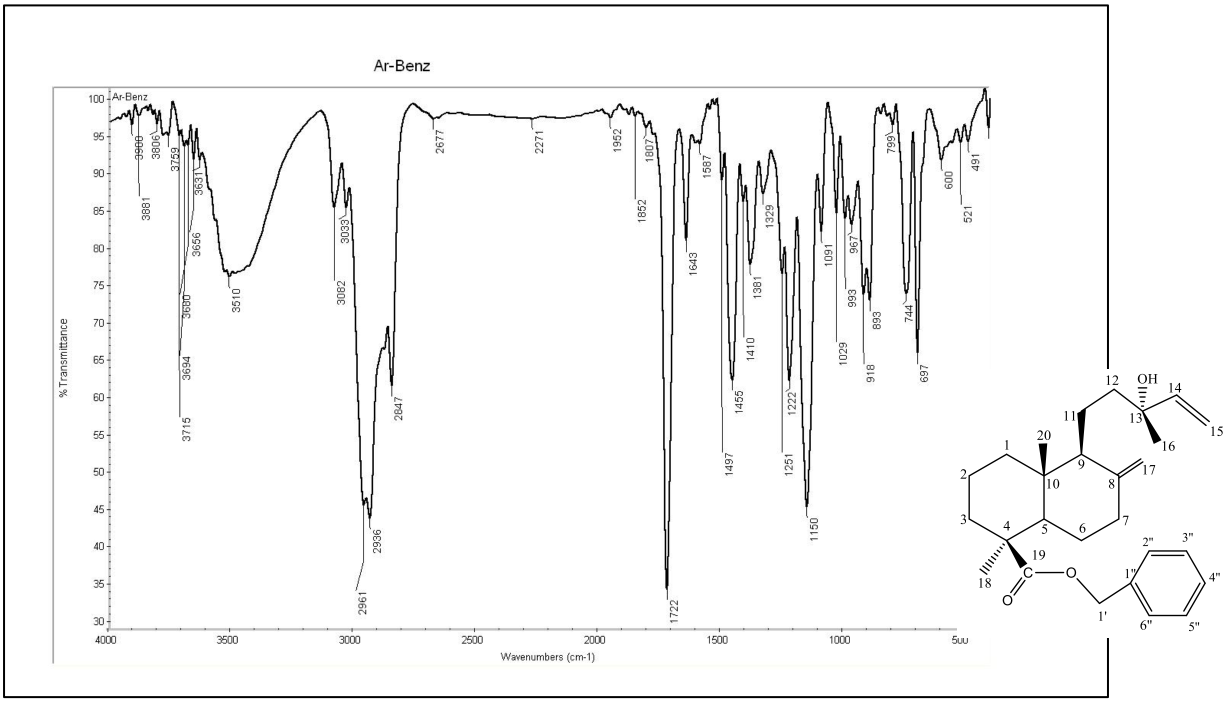

Figure S34. IR spectrum of compound **9**.

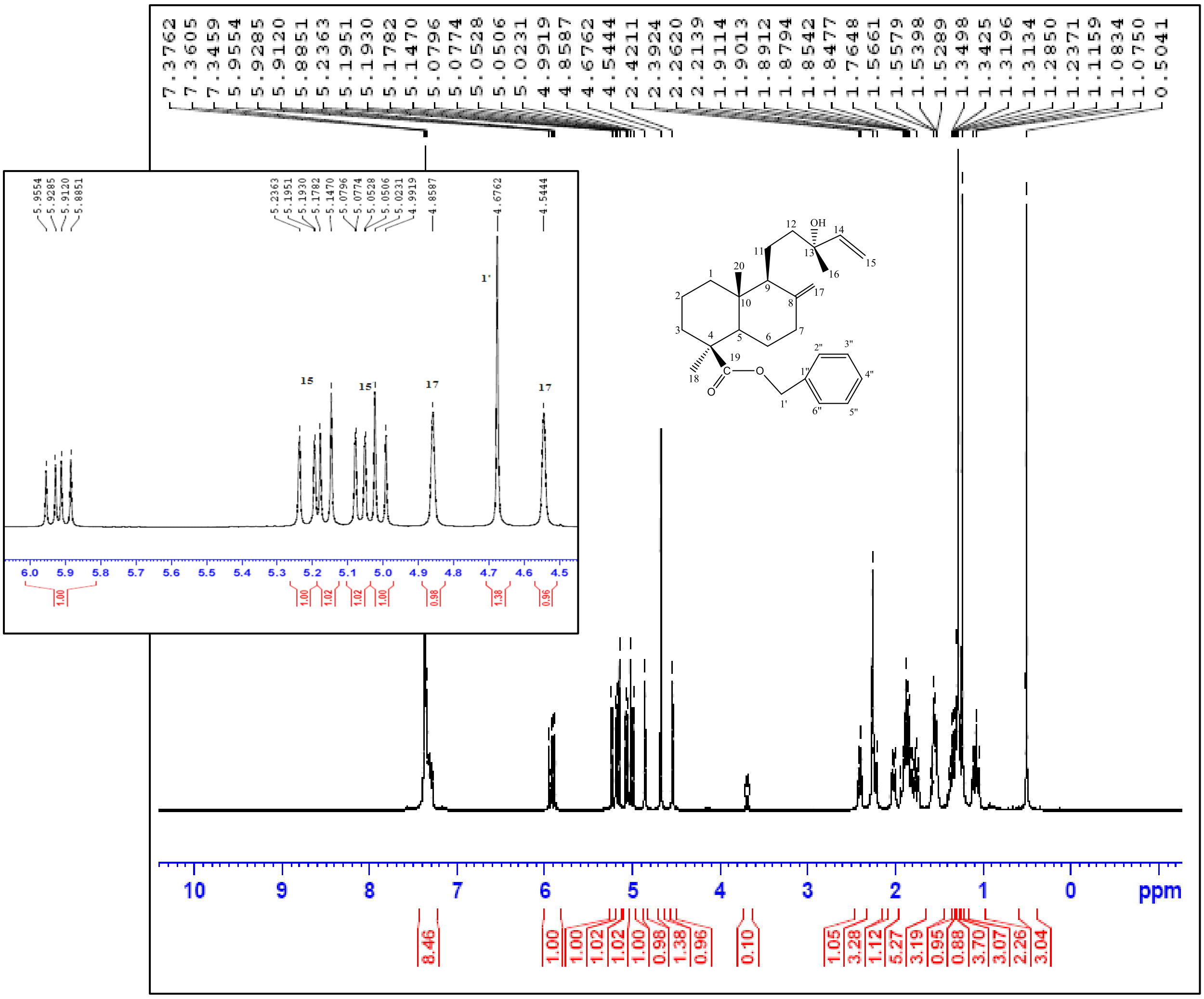

Figure S35. ^{1}H NMR spectrum of compound **9**.

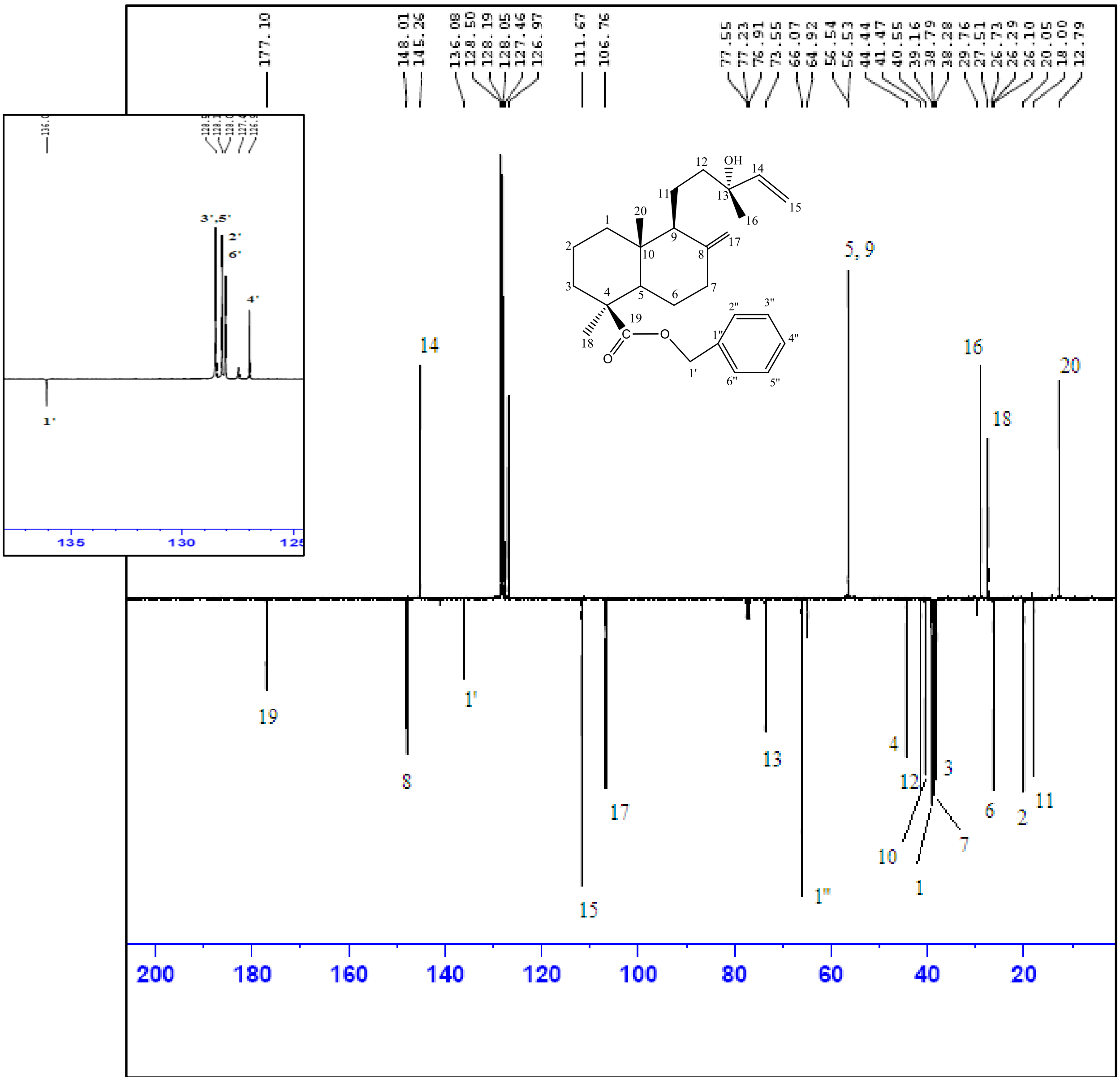

Figure S36. APT spectrum of compound **9**.

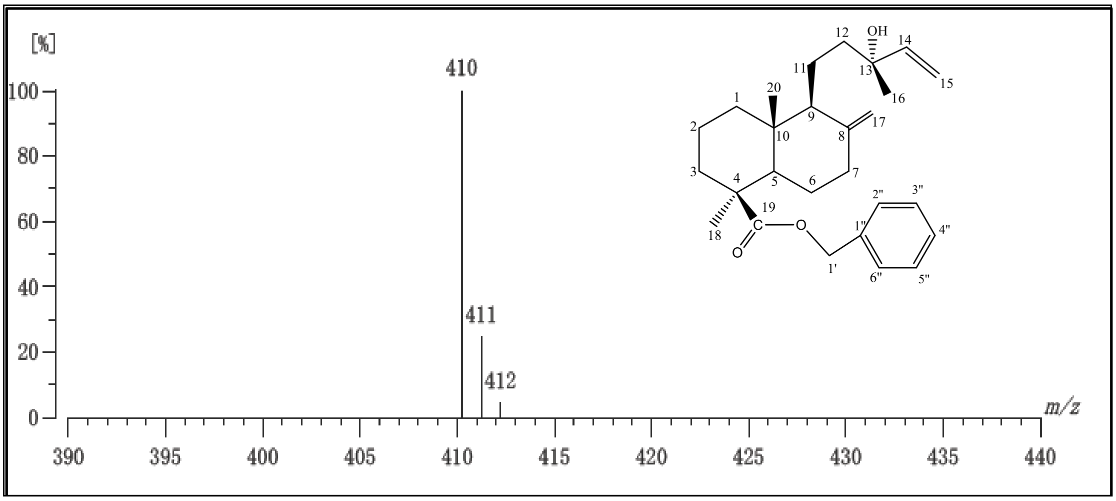

Figure S37. Mass spectroscopy of compound **9**.

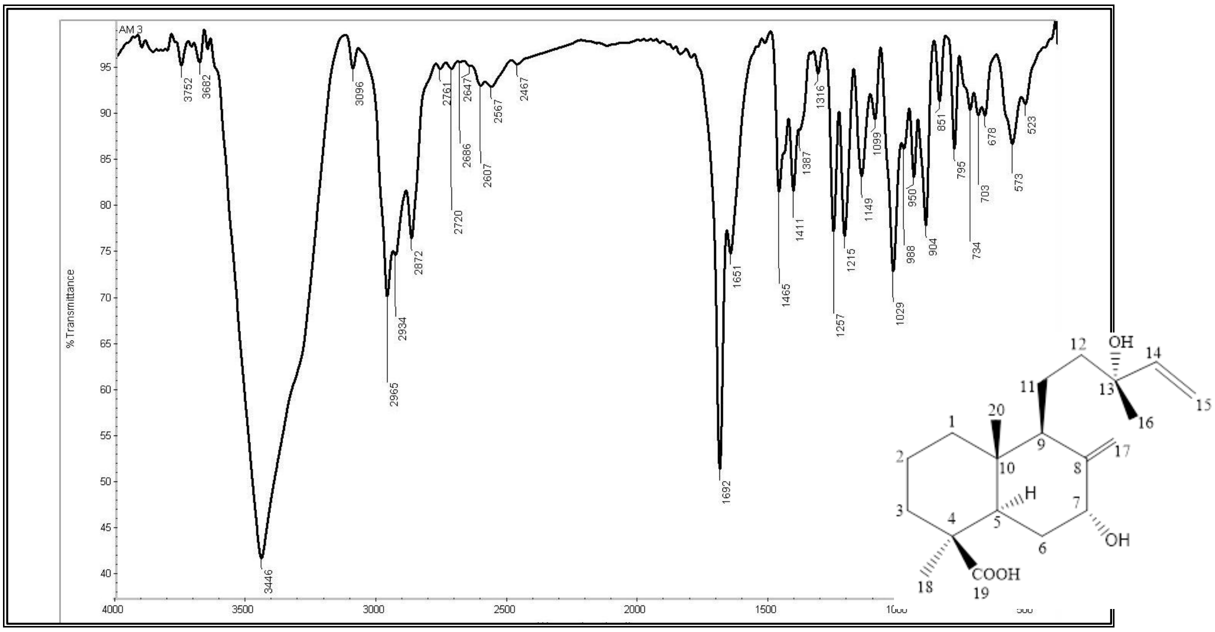

Figure S38. IR spectrum of compound **10**.

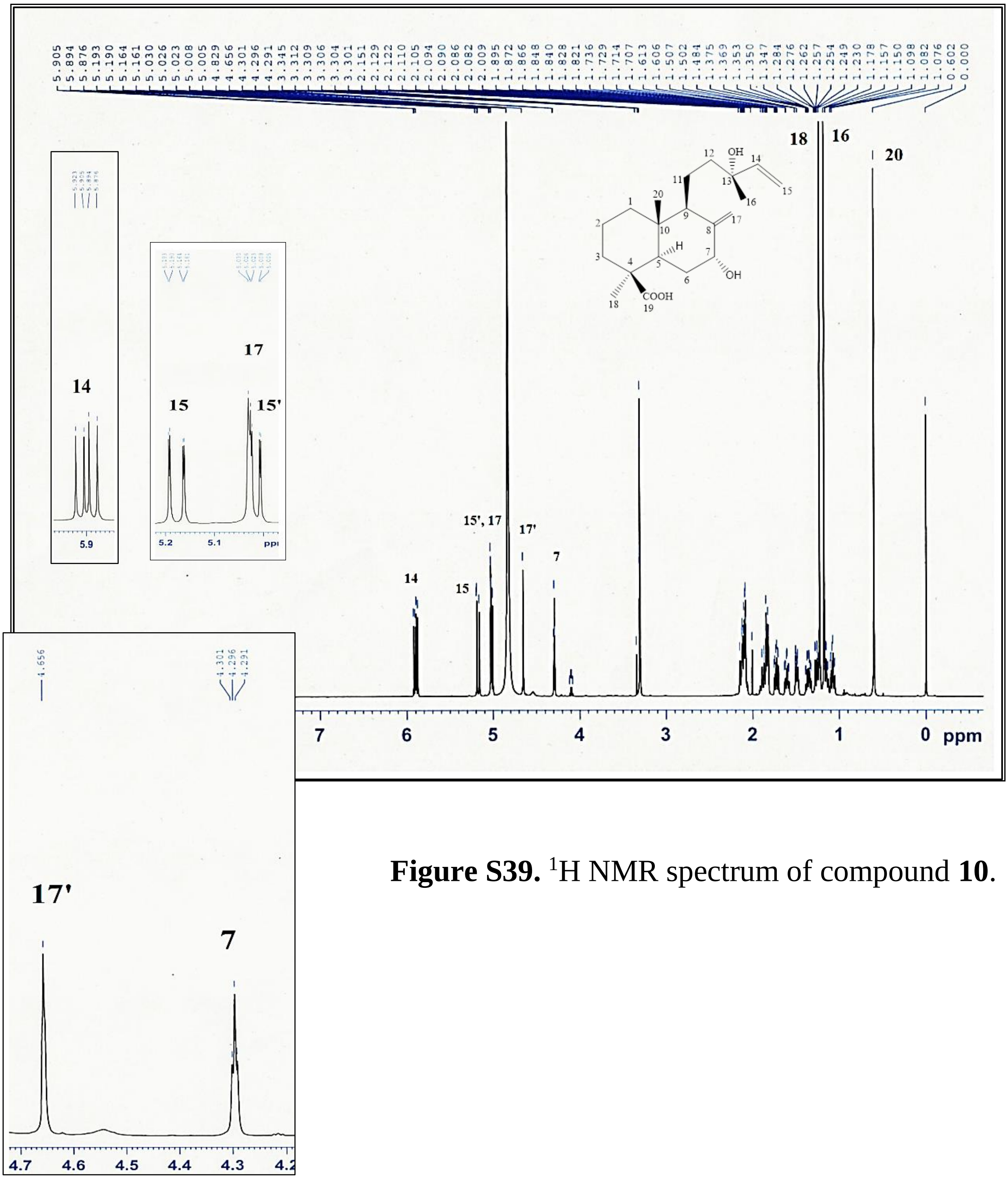

Figure S39. ^{1}H NMR spectrum of compound **10**.

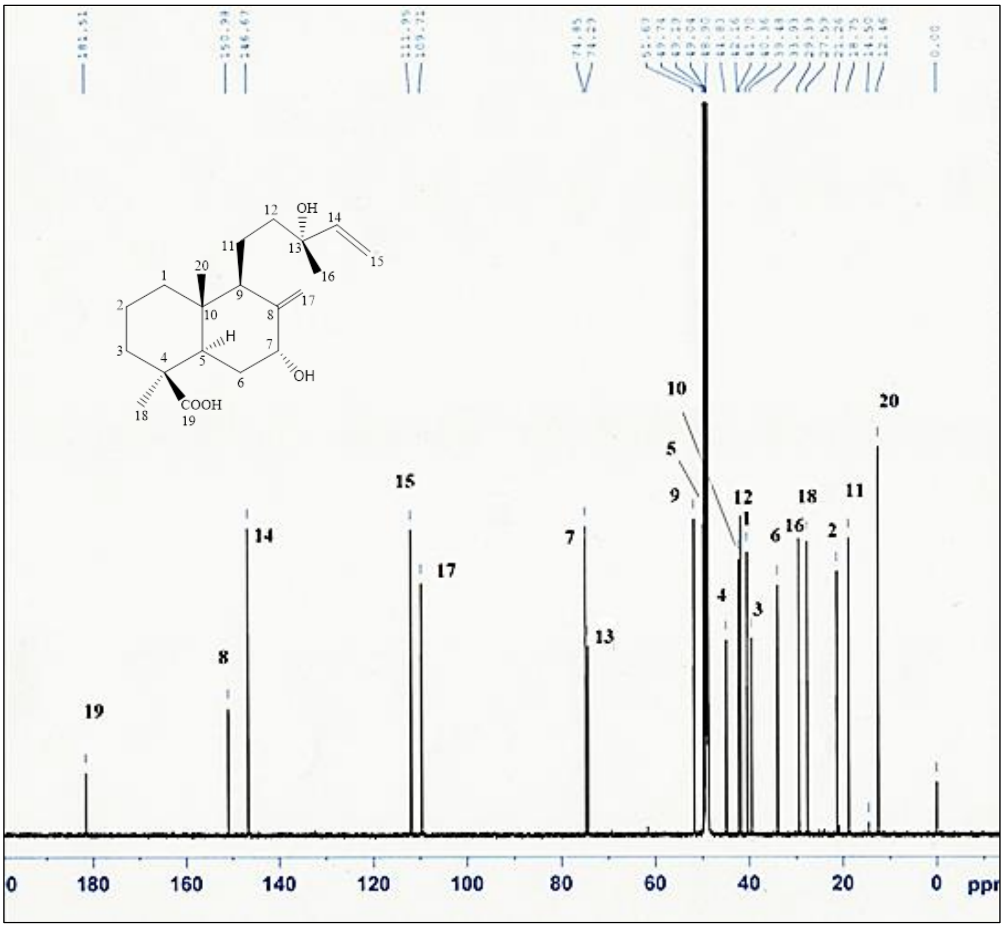

Figure S40. ^{13}C NMR spectrum of compound **10**.

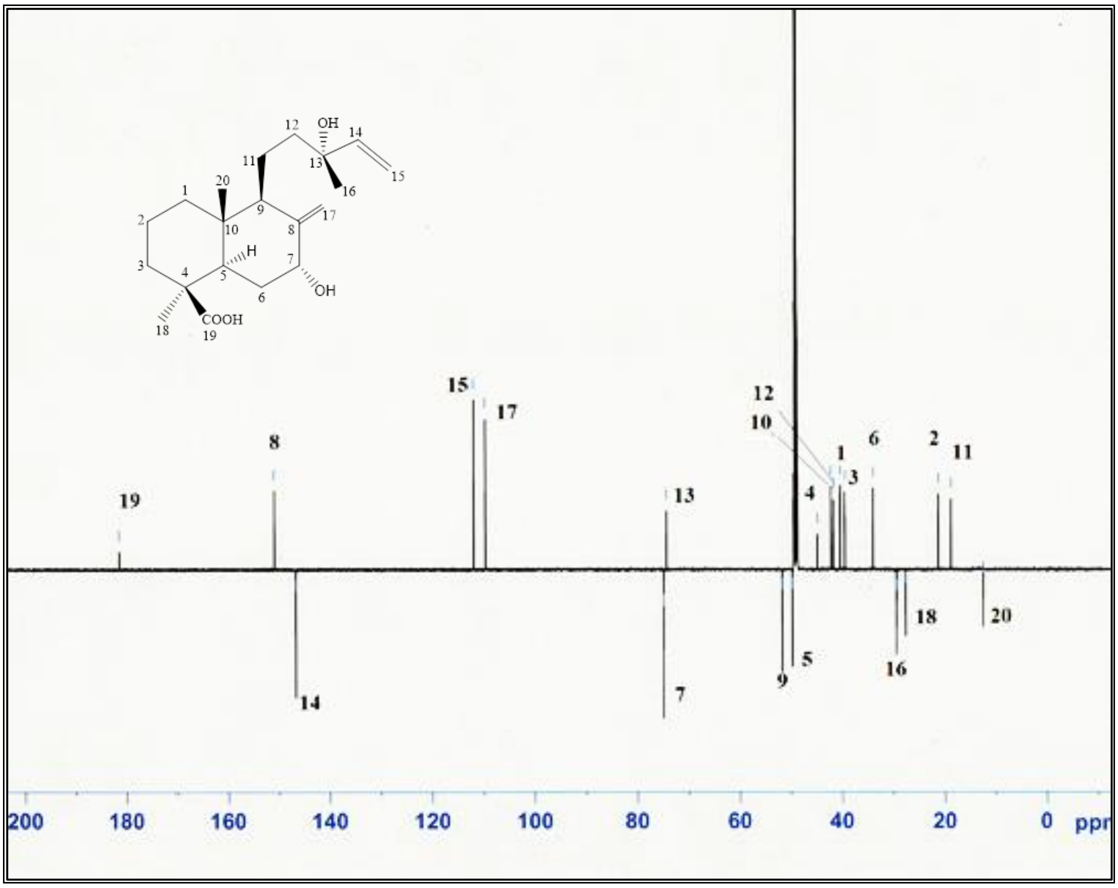

Figure S41. APT spectrum of compound **10**.

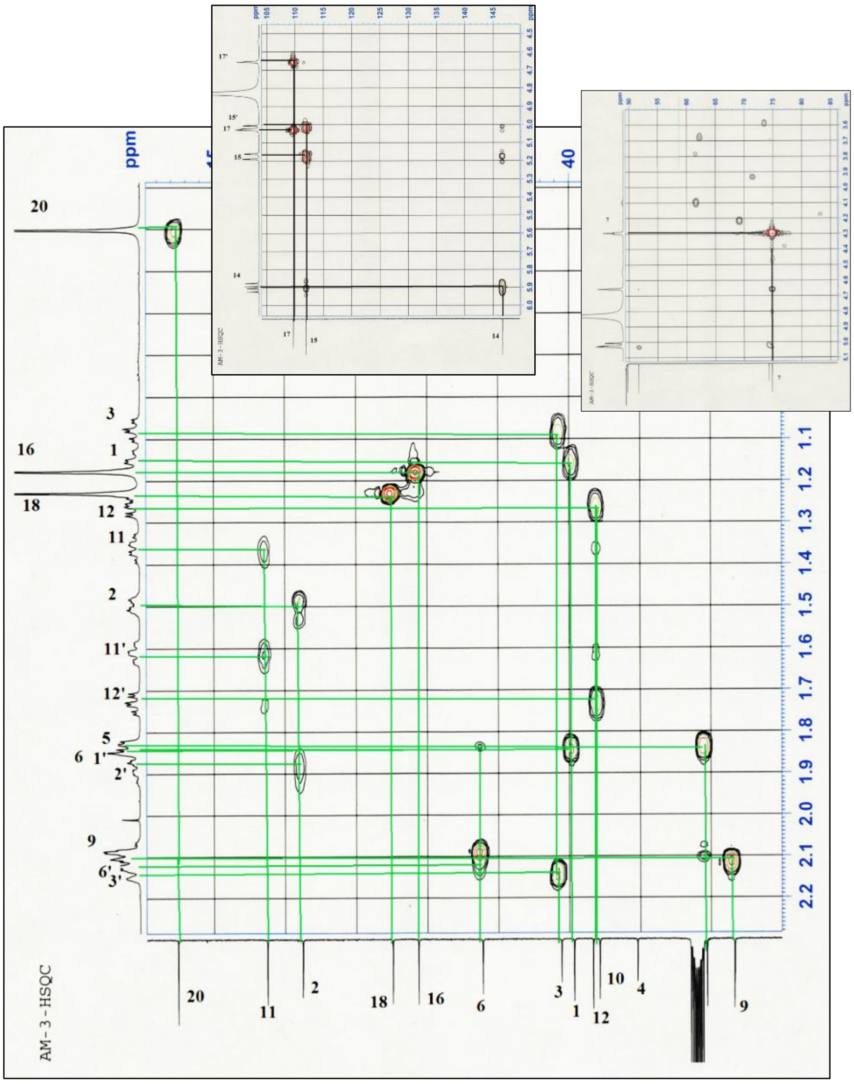

Figure S42. HSQC spectrum of compound **10**.

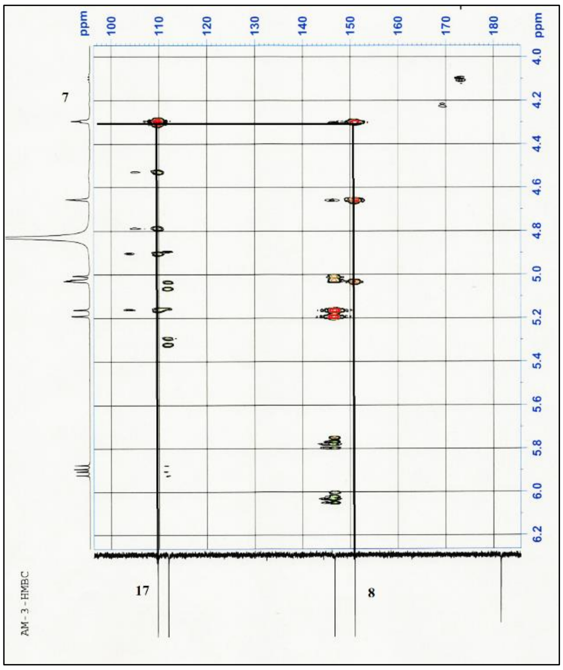

Figure S43. HMBC spectrum of compound **10**.

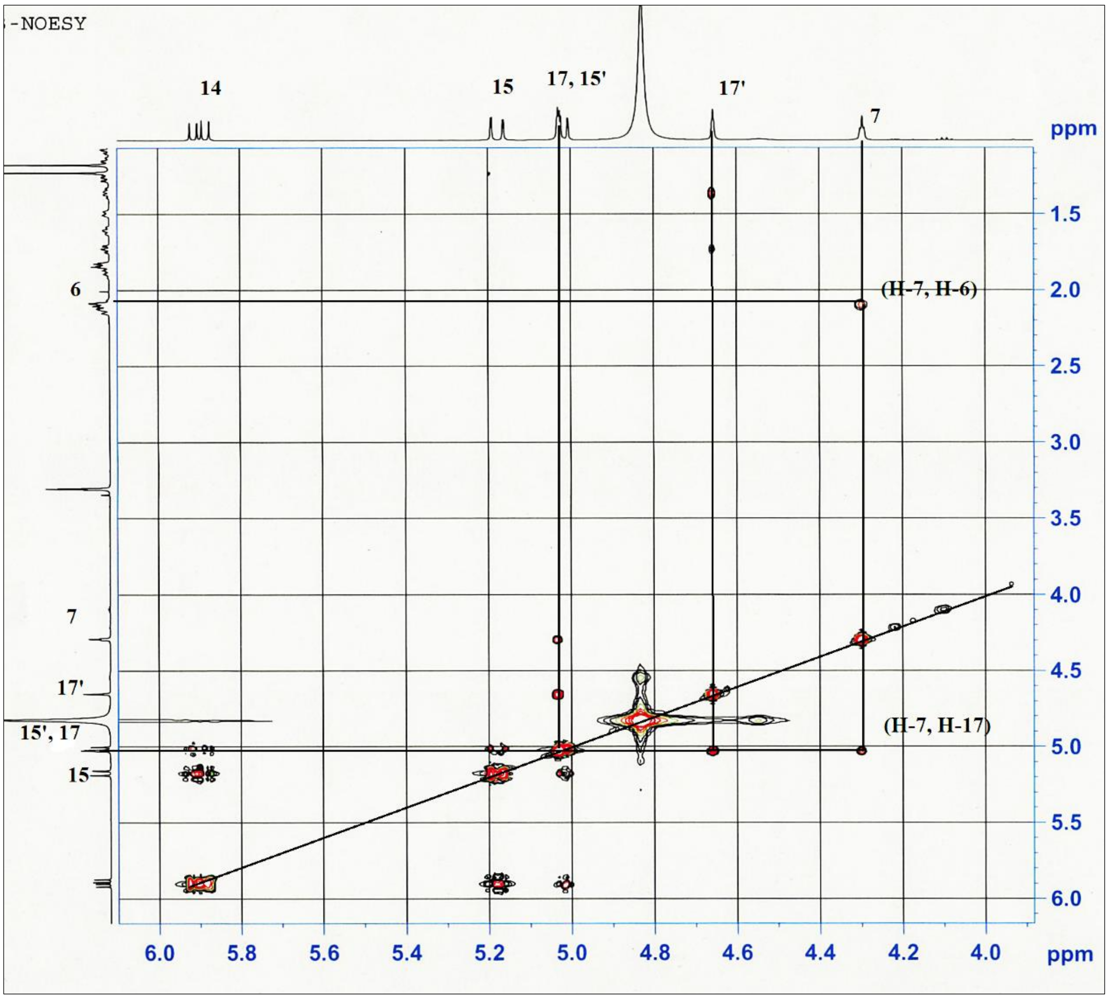

Figure S44. NOESY spectrum of compound **10**.

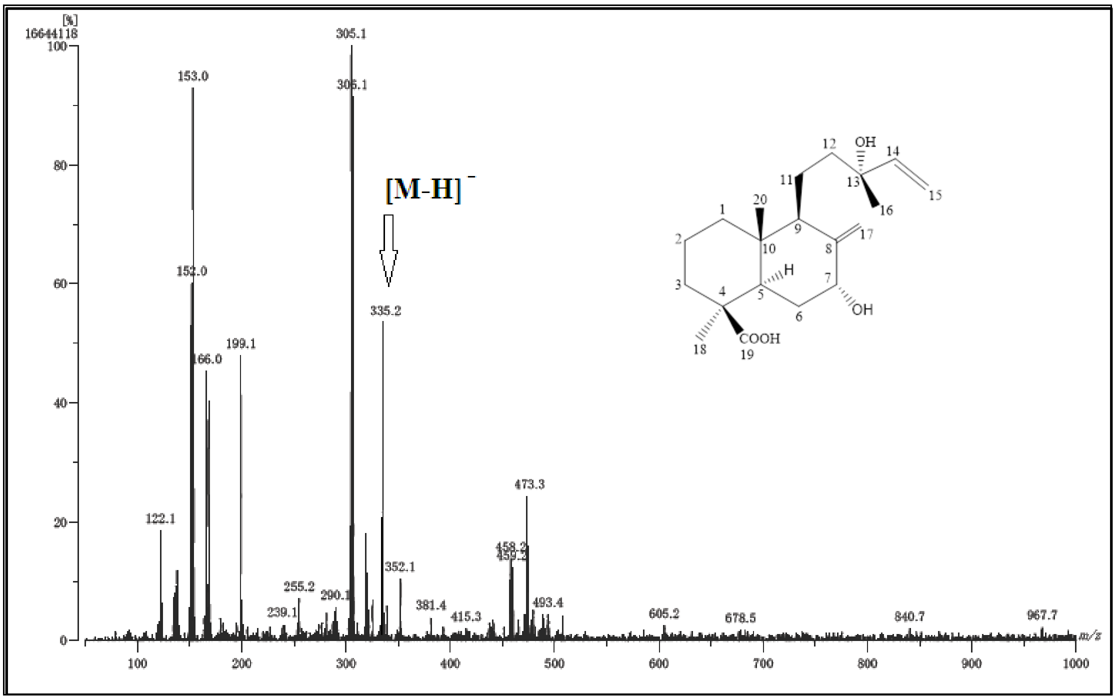

Figure S45. Mass spectrum of compound **10**.

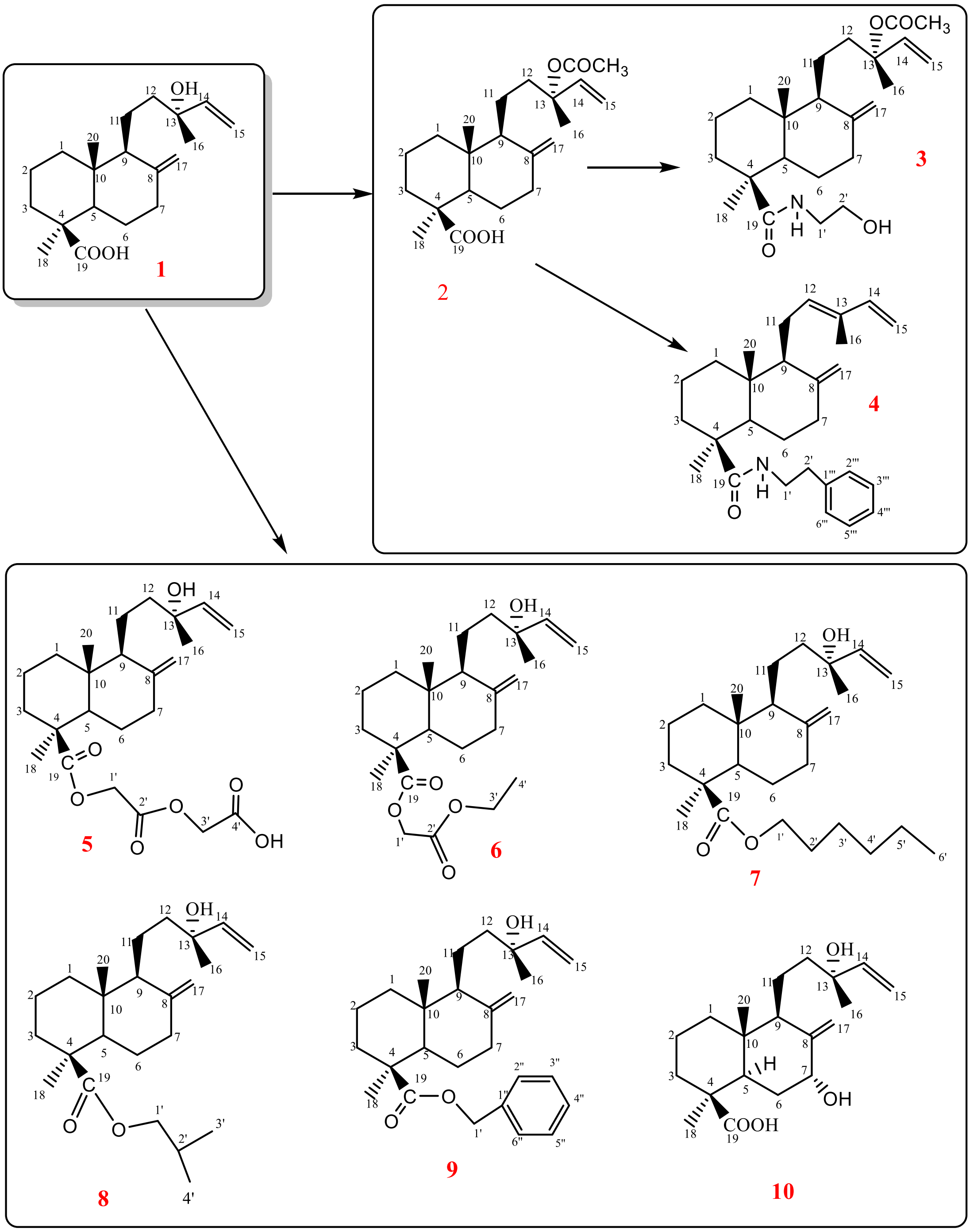

Figure S46. Structures of the prepared cupressic acid derivatives (**1-10**).

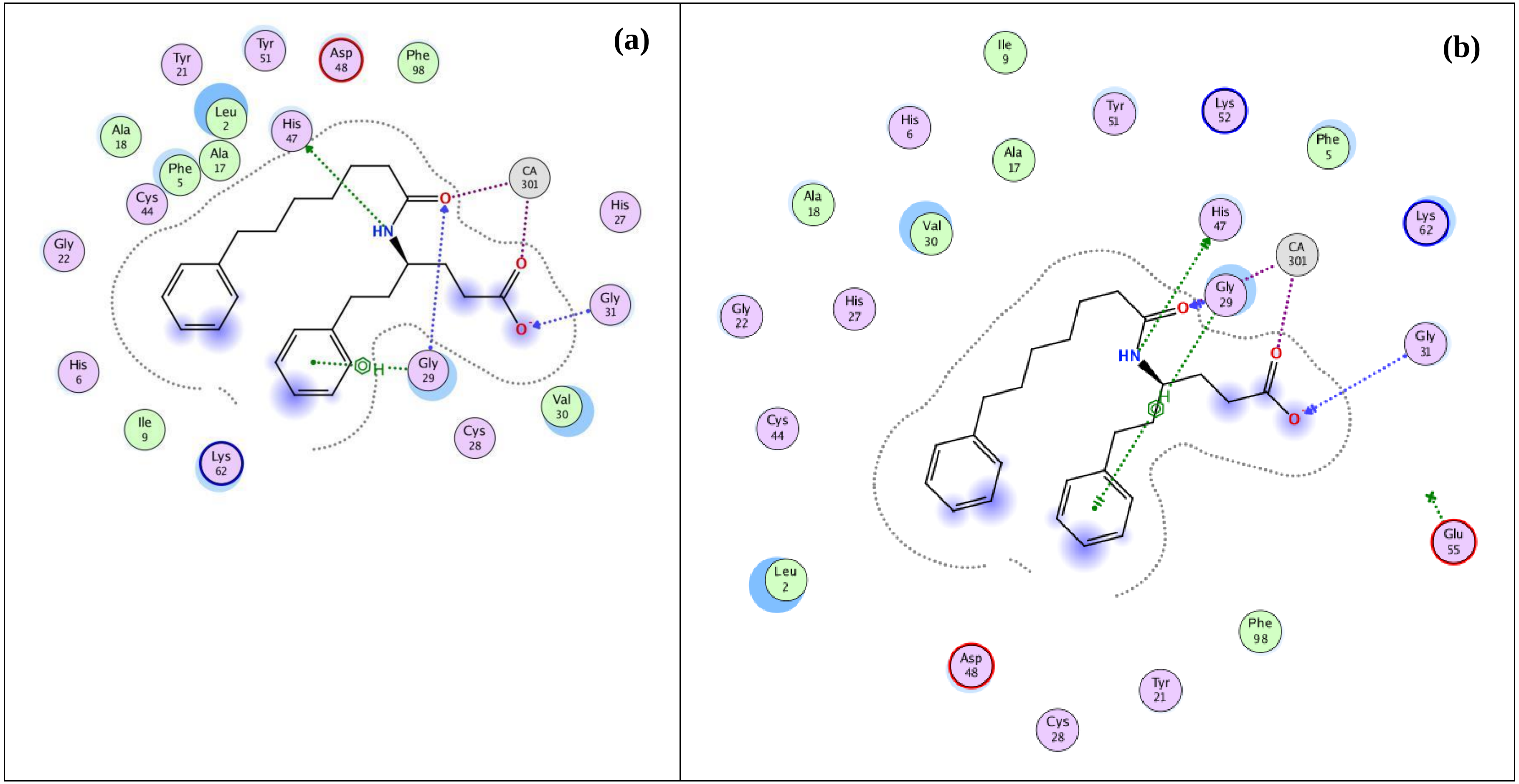

Figure S47. 2D binding modes of interaction of the inhibitor, **BR4** (6-phenyl-4(*R*)-(7-phenyl-heptanoylamino)-hexanoic acid); a) Co-crystallised and (b) docked structure within the active site of phospholipase A2 (PDB code: 1KQU) indicated method validation.

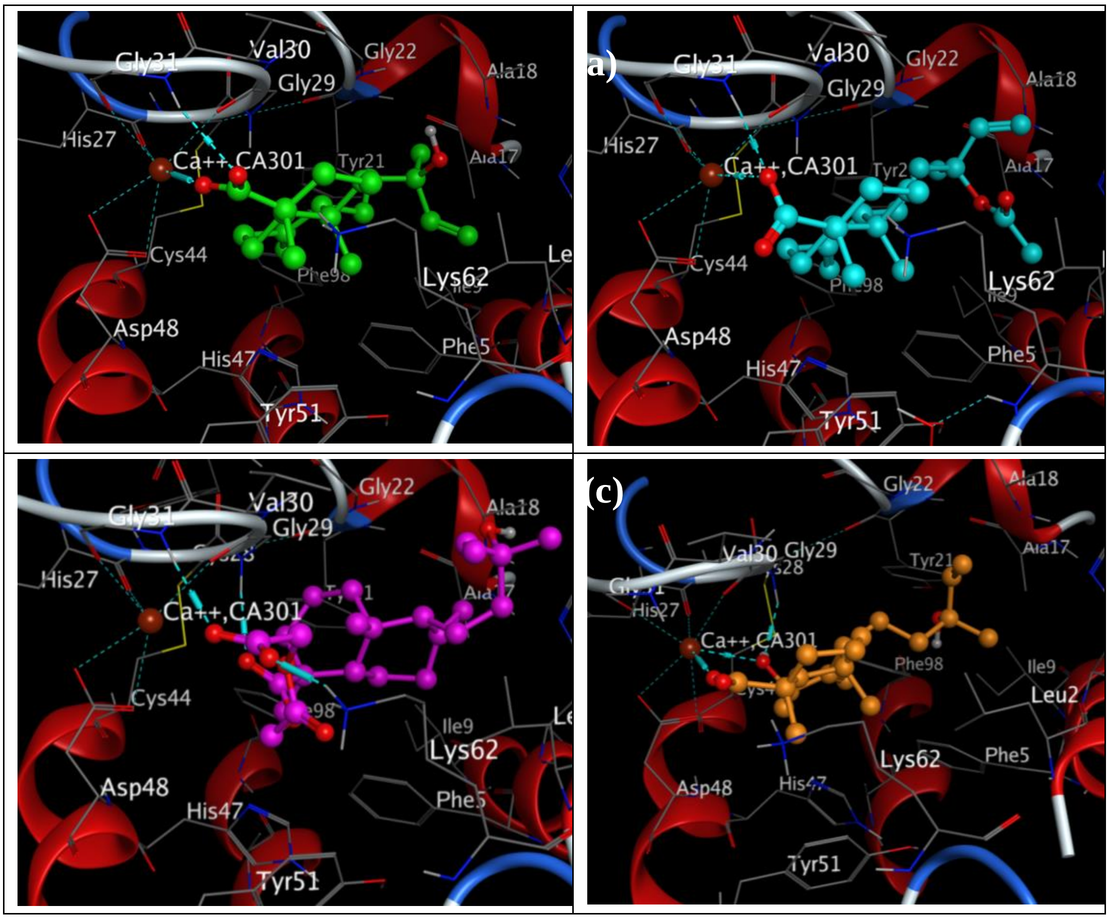

Figure S48. 3D binding modes of interaction of; a) compound **1**, (b) compound **2**, (c) compound **5**, and (d) compound **10** with the active site of phospholipase A2 (PDB code: 1KQU).

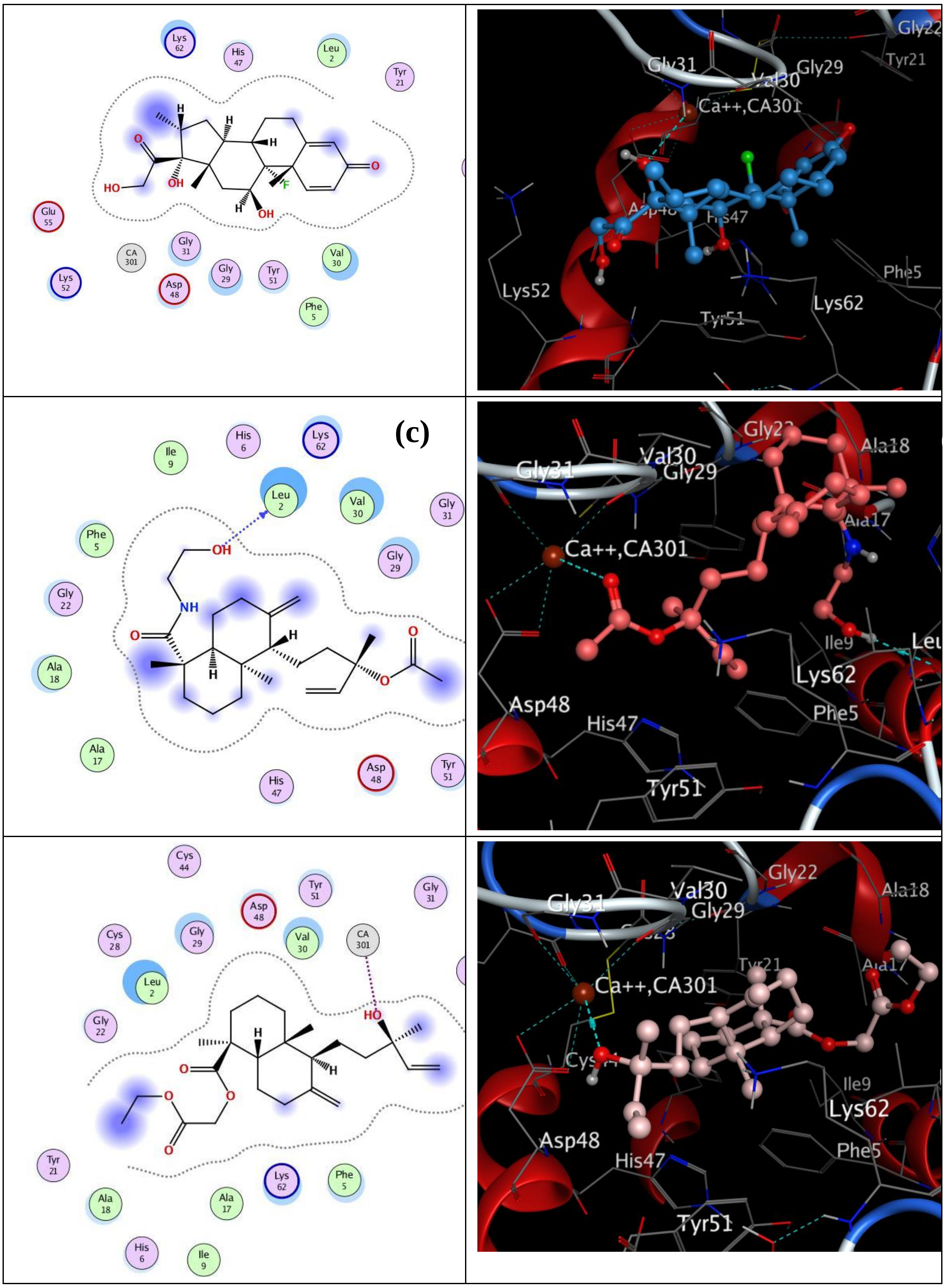

Figure S49. 2D and 3D binding modes of interaction of; (a, b) Dexamethasone, (c, d) compound **3**, (e, f) compound **6** with the active site of phospholipase A2 (PDB code: 1KQU).

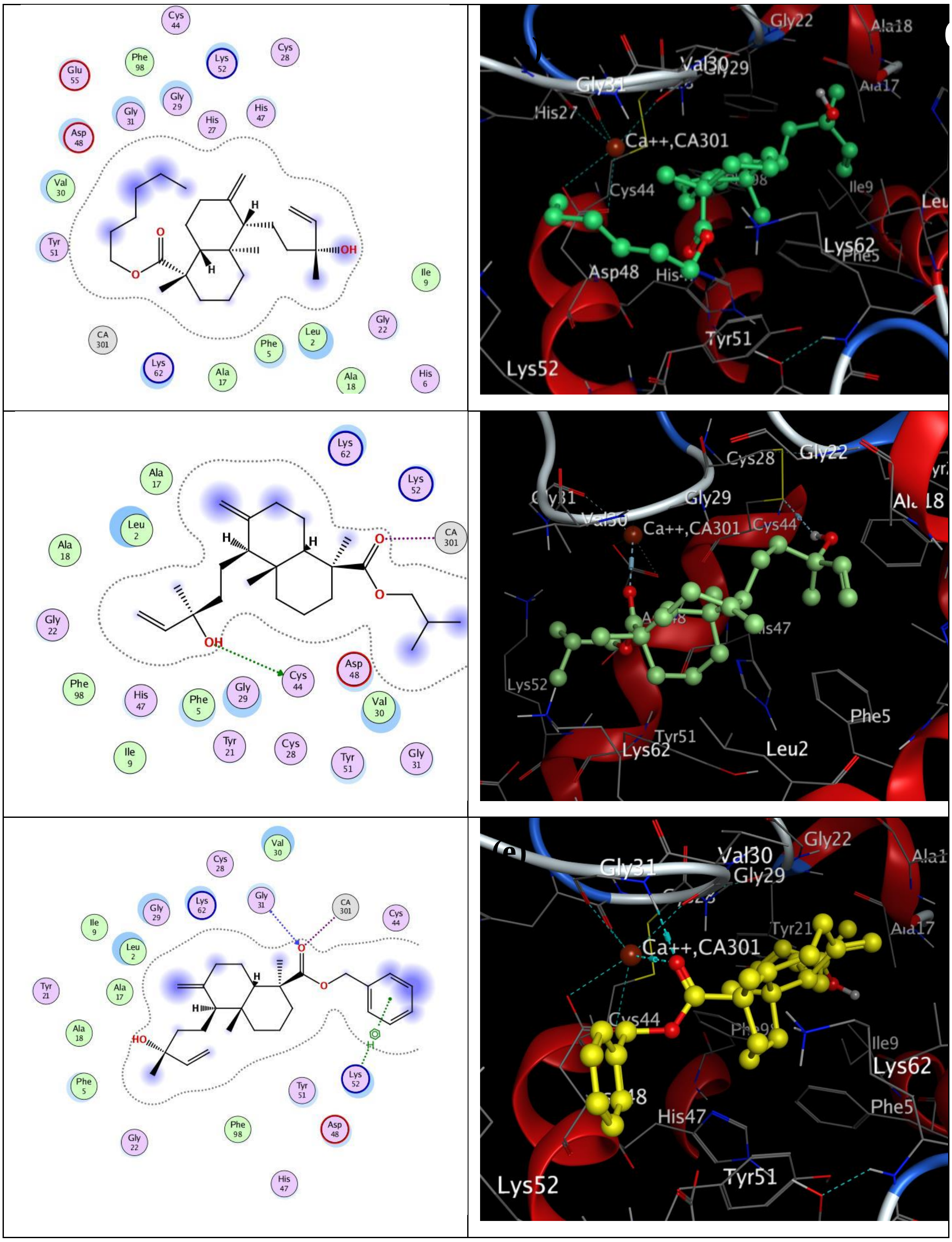

Figure S50. 2D and 3D binding modes of interaction of; (a, b) Compound **7**, (c, d) compound **8**, (e, f) compound **9** with the active site of phospholipase A2 (PDB code: 1KQU).

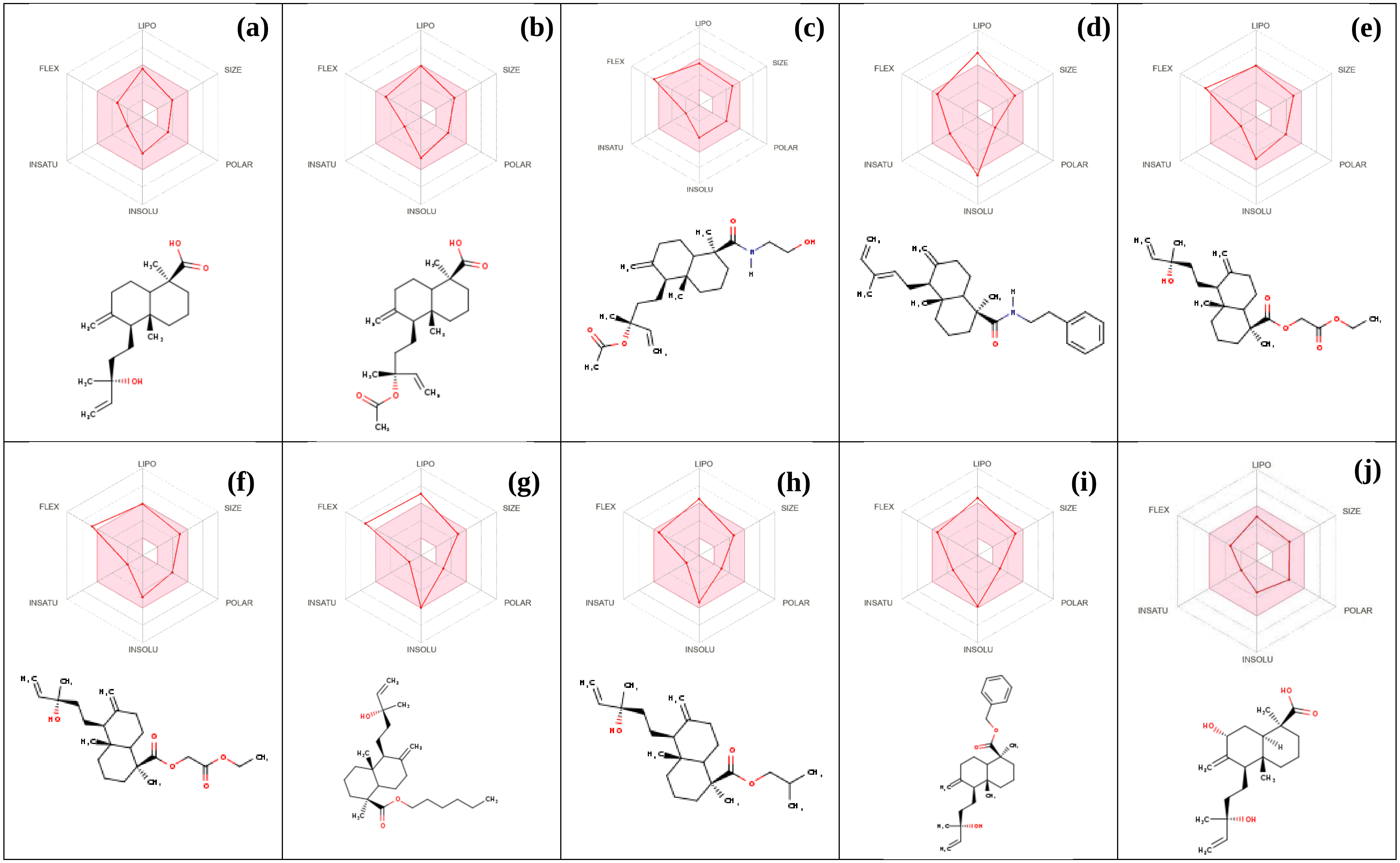

Figure S51. Bioavailability radar figures of the prepared analogues **1-10** (a-j) respectively, using SwissADME online tool (Swiss Institute of Bioinformatics; http://www.sib.swiss) [1].

Table S3. The physicochemical, ADME, and drug likeness properties of compounds (**1-10**) using the Swiss ADME online tool.

**Compound ID**	**1**	**2**	**3**	**4**	**5**	**6**	**7**	**8**	**9**	**10**
**Molecular weight**	320.47	362.5	405.57	405.62	436.54	406.56	404.63	376.57	410.59	336.47
**H-bond (donors/acceptors)**	2/3	1/4	2/4	1/1	2/7	1/5	1/3	1/3	1/3	3/4
**Rotatable bonds**	5	7	10	8	11	10	11	8	8	5
**Lipophilicity (MLOGP)**	3.66	3.97	3.16	5.47	2.68	3.57	4.94	4.53	4.88	2.81
**Water solubility (ESOL)**	Moderately soluble	Moderately soluble	Moderately soluble	Poorly soluble	Moderately soluble	Moderately soluble	Moderately soluble	Moderately soluble	Moderately soluble	Soluble
**TPSA**	57.53	63.6	75.63	29.1	110.13	72.83	46.53	46.53	46.53	77.76
**GIT absorption**	High	High	High	Low	High	High	High	High	High	High
**BBB permeability**	Yes	Yes	Yes	No	No	No	No	Yes	No	Yes
**P-gp substrate**	No	No	Yes	Yes	Yes	No	No	No	No	Yes
**Bioavailability score**	0.85	0.85	0.55	0.55	0.56	0.55	0.55	0.55	0.55	0.56
**CYP450-1A2**	No	No	No	No	No	No	No	No	No	No
**PAINS**	0	0	0	0	0	0	0	0	0	0
**Lipinski; violations**	Yes; 0 violation	Yes; 0 violation	Yes; 0 violation	Yes; 1 violation: MLOGP>4.15	Yes; 0 violation	Yes; 0 violation	Yes; 1 violation: MLOGP>4.15	Yes; 1 violation: MLOGP>4.15	Yes; 1 violation: MLOGP>4.15	Yes; 0 violation
**Synthetic Accessibility**	4.06	4.34	4.44	4.41	5.02	4.94	4.86	4.63	4.53	4.36

References

1. Daina, A.; Michielin, O.; Zoete, V. SwissADME: a free web tool to evaluate pharmacokinetics, drug-likeness and medicinal chemistry friendliness of small molecules. *Scientific Reports* **2017**, *7*, 42717, doi:10.1038/srep42717.
